# Supplementary material for: Diffusion of Lexical Change in Social Media
Source: PLoS One. 2014 Nov 19;9(11):e113114. doi: 10.1371/journal.pone.0113114 (PMC4237389; doi:10.1371/journal.pone.0113114)
Supplement: File S1 — Appendix S1-S3, Table S1 and Software S1. Appendix S1. Term list. List of all words considered in our main analysis. Appendix S2. Term examples. Examples for each term considered in our analysis. Appendix S3. Data Procedures. Description of the procedures used for data processing, including Twitter data acquisition, geocoding, content filtering, word filtering, and text processing. Table S1. Term annotations. Tab-separated file describing annotations of each term as entities, foreign-language, or acceptable for analysis. Software S1. Preprocessing software. Source code for data preprocessing. (ZIP) [file pone.0113114.s001.zip › supp_for_upload/Appendix_S1_Term_List.pdf]

Supplementary Information: Word list

hheeyy	u'd	warms	twerk	qo	it-	leavn
niiccee	iwanna	heats	tango	kum	thiis	droppin
oommgg	imaa	wlk	dougie	kome	dhis	chargin
yyaayy	u'll	plow	pik	waiit	thys	skippin
bomb.com	culd	re-up	mute	elses	dhat	leavin
=d	cld	swerve	twit	happn	thiz	guarding
=p	cud	beez	twitt	i`m	whr	stealin
=o	shud	hangout	twitvid	iim	wut	sendin
gooaall	shuld	livee	shovel	u're	waht	pushin
gooll	shld	workin	fite	something's	wher	usin
shee	mite	wrking	knit	iits	whut	catchin
uve	wud	wrkn	dribble	itz	weneva	breakin
u've	wuld	wrkin	fling	nothing's	wha	bringin
jut	wld	workn	subtweet	everything's	yy	cuttin
jux	iont	sittin	fuxx	thatz	veryy	pickin
jus	dn't	standin	hitt	datz	absolutly	carryin
jsut	wldnt	stayn	bodied	thas	completly	clockin
iaint	wuldnt	stayin	toot	dass	awkwardly	keepn
ain't	shudnt	layin	mow	whas	legitimately	holdin
aint	wudnt	sittn	go2	watz	uber	throwin
ainn	shldnt	chattin	kall	wuts	f*ckin	feedin
aiint	cudnt	dealin	sext	wutz	fuggin	showin
couldve	kant	steppin	textt	whts	f-ing	puttn
wulda	culdnt	otp	fugg	wats	fukkin	hittn
shulda	cldnt	relaxin	gtf	whatss	fuccin	shuttin
wouldve	hafta	checkn	fcuk	whatz	effn	addin
shouldve	qotta	cashin	wink	wutchu	mf'n	scratchin
wudda	knoo	cuddling	smooches	watchu	flippin	switchin
shudda	noe	checkin	shrugs	nobody's	f'n	choppin
wuda	warmers	flirtin	sniffles	smellin	f'in	tearin
shuda	excite	grillin	faints	feeln	effin	givin
mustve	beleive	mackin	kanyeshrug	feelin	s0	rubbin
nevr	gaf	arguin	squee	soundin	liike	puttin
neva	4get	mobbin	pouts	dressin	liek	settin
eva	rememba	chillaxin	sideeye	wus	lk	lickin
evr	memba	wakin	winks	waz	boutt	postin
evar	luvv	gearing	shruggs	wass	havin	hittin
onli	luv	waken	grins	wuz	havn	touchin
evn	nominate	snuggled	shrug	iz	beinq	tackling
eem	h8	turnt	ahem	izz	get'n	installing
realli	thnk	fukked	mjb	;s	gettn	takin
alrdy	thght	racked	sadface	buhh	follown	expectin
cuda	4got	chk	pause	pero	followin	wearin
allready	thk	checc	cosign	esp	callin	takn
alreadyy	quess	shouts	syh	i.e.	treatin	despicable
offically	gotchuu	matata	fwm	i.e	helpin	buyin
finaly	swea	d/l	holla	iwant	lettn	makin
ion	hav	preorder	hmu	gimmie	ignorin	makn
uma	qot	occupy	co-sign	whys	judgin	watchinq
casually	favorited	pre-order	laff	imiss	lettin	watch'n
deadass	hadd	nano	likey	ilovee	unfollowin	watchn
obvi	pre-ordered	sync	muero	sumtimes	testin	watchin
alwayz	copped	conditioned	clinch	evrytime	killn	hearin
alwys	preordered	shun	reinstall	iguess	answerin	findin
definitly	aced	twug	install	mayb	teachin	likin
defiantly	missd	slander	decorate	altho	stalkin	surfin
definetly	askd	scramble	transform	methinks	calln	blastin
definately	tol	punt	tackle	becuase	askn	luvin
qonna	bbm'd	flee	discover	b/c	askin	enjoyin
bouta	calld	draw	violate	kause	bbm'n	readin
fina	startd	sweep	deactivate	wheneva	telln	downloadin
gne	sed	spill	followback	becuz	stoppin	rockn
trynaa	knos	sabotage	qet	bcuz	losin	celebratin
fenna	knws	reboot	takee	eventhough	changin	draggin
letss	luvs	wobble	makee	what're	updating	grabbin
letz	forgives	swim	giv	iif	payin	coppin

Word list: Page 1 of 6. All 2,603 words used in our main analysis. They are ordered by the hierarchical word clusters of [1] (http://www.ark.cs.cmu.edu/TweetNLP/) which tends to group words with similar syntactic or semantic properties. The lowercased forms are shown, which sometimes is not the most common form; for example, ":d" is usually written as ":D".

smashin	some1	swagged	bak	dancin	editing	aqain
duin	evryone	dunked	bakk	ringin	blogging	l8r
doinn	evry1	lookd	baq	sleepn	unpacking	lata
doiin	every1	swam	bacc	beastin	syncing	sumtime
doin	evrybdy	sacked	bac	snitchin	scanning	nomo
doinq	everyonee	bullied	riqht	cryin	grading	2u
eatin	evrybody	benched	righ	hollin	decorating	tbh
grilling	oomf	nominated	riite	flopping	knitting	ftl
cookn	oomfs	ranked	rite	breathin	coding	afterall
eattin	meeka	snowed	rght	twerkin	designing	mehn
bakin	no1	installed	ritee	choosin	pitching	nshit
cookin	whoeva	launched	rii	laffin	flooding	jor
drinkn	waitn	unplugged	ryte	twerking	marvins	neways
cravin	waitin	leaked	schemin	laughn	marvin's	leh
drinkin	rootin	hosted	hydrated	laughin	openin	anywayz
mert	feenin	stung	storming	stylin	mowing	nemore
mehh	searchin	playd	rainin	swaggin	shoveling	neway
mhe	lookn	hoed	poppin	trickin	cleanin	2me
urself	look'n	subtweeted	snowing	performin	stocking	lah
yaself	commin	sampled	poppington	pumpin	cooling	4me
yurself	comin	wantd	happenin	flexin	finishin	2m
hym	comn	4ward	goodie	starin	signin	yest
ypu	cummin	starvin	poppn	hooping	fillin	4u
yall	upgrading	singlee	snowin	spinnin	wrapping	lastnite
ya'll	startn	outtie	popin	blazin	washin	evryday
y'all	startin	preggers	hailing	jerkin	cashing	errday
yeen	plannin	siick	wronq	tweetin	passin	rn
iget	omw	sauced	premieres	planking	peeling	tmo
yhuu	headin	faded	rox	cuffin	drivin	yesturday
yhu	enroute	preg	suxx	packin	travelling	doee
y0u	s\o	sunburned	sux	studyin	dunking	thoe
juu	s/o	sunburnt	snows	trolling	walkn	doe
yoy	shoutouts	tite	wrks	writin	beaming	thoo
iht	s|o	odee	tackles	subtweeting	walkin	thoee
iit	s/0	maad	scrolls	twittering	shuffling	thoughh
yurs	qoinq	od	presents	protesting	up-	now-
urs	fixin	madd	mower	grindin	uhp	ther
evrything	crackn	embarassed	toasty	bbming	uprt	heaa
everythin	jumpin	appalled	cozy	chokin	owt	4eva
everythng	shakin	dissapointed	warm	fightin	0ut	4ever
nuttn	rushin	suprised	rigged	texting	out-	sumwhere
nutn	crackin	butthurt	postponed	fasting	ova	forsure
nuffin	goiin	dgaf	trendin	twitting	ovr	manually
nuttin	movin	xcited	undefeated	recordin	ovaa	4sure
nutin	qoin	talm	deadd	graduating	ovah	lyrically
nuthin	stickin	talk'n	saucer	sexin	arnd	eitha
nothn	listenin	tlkn	2go	spellin	outsidee	,.
nuthing	willin	bitching	bk	studying	w/u	;;
nuthn	sposed	talkn	tangled	blockin	w/me	);
nthn	refering	braggin	doobies	singin	2gether	,?
nun	cooled	talkinq	sweatin	mixin	2getha	??..
sumtin	mowed	complainin	slackin	sharin	2morro	.?
sumthn	deactivated	speakin	chirping	slammin	2morrow	.??
sumthing	spendin	thinkin	thuggin	twatchin	tomar	?!..
somethn	wastin	thnkn	partyin	datin	2mrw	.!!
smthn	violated	talmbout	lien	cuffing	2mor	,!
summin	dvr'd	jokin	wildin	typin	2mrrw	.!
sumthin	muted	forgettin	knockin	smackin	2moro	!!..
suttin	graduated	claimin	buzzin	subtweetin	2maro	ndd
anythin	bugged	dunno	cheatin	scheming	toma	&'
nething	swept	wonderin	frontin	hidin	2morow	&&'
nebody	walkd	debatin	buggin	flexing	2day	(&
any1	rained	pondering	dyin	twatching	2nite	be4
ne1	fouled	guessin	speedin	learnin	tonite	b4
sumone	biked	hopin	preachin	uploading	tnite	witout
sum1	ducked	knowin	coughin	writting	2night	aftr

Word list: Page 2 of 6.

afta
t0
0f
4the
w/a
w/my
w/the
w/this
widd
w|
unda
wiit
frum
frm
4rm
n2
in2
btwn
durin
w/in
0n
iin
i'n
tge
thaa
dha
onna
inda
2my
yhur
urr
beyonces
yurr
ure
nicki's
somebodys
google's
rihanna's
beyonce's
hiz
year's
blog
headlines
downloads
chronicle
q&a
index
cc
fw
sn
psa
attn
sidenote
np
wts
null
cont
via
itrt
urt
nfb
swine
-*
punya
cade
pow

mem
supp
vox
nit
ren
ff
det
sk
aff
lat
stu
rm
wr
hu
int
rb
ent
thr
u's
gz
sp
baken
ft
fah
fir
ov
w/that
w/her
mos
hurd
derp
lml
lbvs
lolx
lolss
lls
l0l
lolzz
lols
wkwk
me2
rft
rotflmao
yessirr
rofl
kml
sheeshh
ctfu
ctfuu
leggoo
roflmao
legoo
rotfl
kmsl
bol
sheesshh
hyfr
yezzir
4sho
lma0
rotf
bwahahahaha
jajajajaja
jajajaja
aha
tqm

heehee
jaja
ahaa
jajaja
hihi
jajajajajaja
wkwkwk
jajajaj
jajaj
inshallah
yuup
yehh
nawl
noes
werd
iknow
nawh
yh
wordd
ikr
ayye
yuupp
asdfghjkl
omo
ooww
waahh
wheww
oww
owee
wooww
zomg
ayee
uugghh
wheeww
ggrr
omq
p.s.
awee
awhh
imy2
oow
oke
aaww
awh
wahh
tuhh
awl
uumm
huuhh
hhmm
kthx
brr
sheesh
whew
nbd
arghh
argh
geezz
uughh
wheew
blech
geesh
ick
arrgghh
arg
ewh

whelp
hmph
hmp
uugh
uqhh
urghh
urgh
hmmph
welp
uuggh
uqh
grr
wowzers
arrgh
thanxx
thankx
thnx
thankz
thanx
saludos
thanxs
thnxx
srry
sry
felicidades
grats
congratz
twugs
booyah
hooray
whoohoo
yummyy
yipee
woo-hoo
hoo
whee
leggo
ftw
huzzah
woot
yuumm
muah
tgif
w00t
gobble
rah
burr
meow
gudda
ayo
cmon
gmorning
brb
g'morning
laters
g'nite
gnight
ilyy
ilh
gnite
iloveyou
imy
goodnite
g'night
goodmorningg
ttyl

imyy
toodles
imu
hbd
plzz
hunh
abi
hbu
wbu
wby
whassup
wassupp
wussup
goodlookin
wydd
tf
wusup
df
wzup
waddup
wadup
wattup
4real
wuddup
watup
whattup
wyd
wya
whatup
wsup
ik
iono
idek
iknw
becareful
idgaf
amo
amoo
whatev
ig
wuteva
busta
whateva
gtfo
aiight
aiite
ard
iite
ookk
s2
<3333333
<333
<333333
<3<3<3
<33
<33333
<3<3
<3
<3333
b-)
;3
-
u.u
\m/
;]
;-d

8-)
>:d
;-p
>:)
:3
(;
toort
^.^
=)
=]]
:}
(':
(:
^_^
=]
:')
^-^
((;
^__^
((:
;))
:))
cx
._.
haga
o__0
u_u
o__o
0_0
:-o
o_0
0__o
o.o
(-__-)
o_o
0_o
x_x
o:
:oo
/:
:"(
</3
)':
;-(
:-|
.__.
:-/
://
='(
-.-"
=|
--__--
:-\
>.<
=/
-_-"
=(
-__-
d;
;/
>:o
-,-
-.-
x__x
/;
<_<

:'/
:*(
=[
-__-"
:,(
-_-
>_<
:((
>__<
""
lok
hakuna
love-
(*
*)
chuuch
gnr
foh
smhh
tyna
smfhh
fyl
kmt
j/k
guhh
rns
goodtimes
frfr
iswear
ijs
j/p
(:<
<<"
<
<-
{
[[
<~~
<==
`
<<
lol-
\3
^_
|3
^
np-
drake-
viendo
>
voice)
}
>>"
->
::
>>
merry
jagged
/mi
sportacular
]
)):
[
f/
yg
ng

Word list: Page 3 of 6.

/via	humps	timeline	tweoples	cookouts	foams	niqht
/cc	boobie	cuzzins	twits	alphas	nudes	moorning
hahart	tweep	homegirl	tweeple	spammers	subtweets	mornin
smhrt	jeezy	tl	twitterverse	spiders	timelines	mornting
lolrt	9700	boothang	twam	macs	mentions	mawnin
lmaort	heffa	homegurl	twiggaz	savages	twitpics	morninq
oan	nigha	bestfriendd	tworld	tornados	e-mails	morn
inches	bih	besty	ya'll!	bigs	twitcons	day-
chainz	nikka	twifey	bloggers	seniors	avi's	semester
wks	nicca	meech	troops	vampires	skillz	wk
loko	nukka	roomate	commentators	turntables	viruses	decade
lokos	nucca	co-worker	grads	fakers	piles	wkend
tds	bihh	followerss	promoters	referees	pix	wknd
rebounds	twigga	follwers	miners	allergies	wrds	shidd
assists	wuss	fren	interns	jumpers	vowels	shytt
innings	g6	bff's	graduates	blisters	disappointme	schoolwork
td's	spammer	bestfriends	travelers	brackets	nts	taxes
pts	slacker	thngs	execs	sacks	turnovers	hw
yards	bish	ladys	crews	snapbacks	fouls	homework
flags	mfer	lass	riots	bikinis	leagues	hmwk
yds	groupie	girlz	dj's	weaves	tryouts	errands
km	bumb	nupes	bots	dunks	meetings	enuf
min/mile	cornball	gurls	unions	helmets	medals	enuff
ln	nupe	ratchets	shoppers	scarves	pools	hickies
utc	heaux	refs	users	mints	camps	matta
pst	dubb	shorties	tablets	beatz	penalties	tyme
hunnit	thugg	gurlz	djs	quizzes	pitches	tiime
veces	dweeb	bruhs	producers	exams	finals	thng
yd	hoee	sistas	qbs	wigs	touchdowns	thinq
cpl	mf	grls	mc's	toys	vids	werk
baybee	qirl	niccas	stans	stockings	billboards	dreamland
hommie	gyal	mf's	candidates	sweatpants	lists	k.o
hunnie	grl	suckas	developers	textbooks	cribs	multitask
hunni	dag	heffas	receivers	hoodies	badges	intermission
hunn	wrd	heauxs	accts	boots	dvd's	midnite
sandz	guh	shyts	haitians	sweaters	files	soundcheck
homey	kellz	mfs	republicans	coats	avatars	fantasy
booskie	shid	mofo's	hackers	cd's	hashtags	halftime
brahh	shiid	hoes	libras	uniforms	blogs	h.s.
hun	bwoy	bishes	leos	mixtapes	ringtones	tims
siss	manee	nighas	protesters	dollaz	functionality	regionals
dawgg	b-day	nikkas	jamaicans	lacefronts	mixes	skewl
booski	bdayy	chics	scorpios	fitteds	beaches	skoo
mamacita	gabba	guyz	celebs	pins	presentations	skool
sweety	gambino	twitfam	virgos	pumps	debates	twitterjail
famo	axx	pplz	bikers	dreds	podcasts	recital
sonn	asx	guise	mosquitos	cargos	icons	class
hon	arenas	twitterworld	crooks	tees	polls	exam
soror	daddys	twitters	tornadoes	blankets	dvds	orientation
baee	neos	twitches	roads	tights	forums	midterm
meng	roomates	twitts	lasers	sandals	quo	homeroom
bestiee	homegirls	ya'll!!	fireworks	essays	feedback	klass
cuhh	exs	tweople	barbies	boyshorts	updates	rehersal
lovie	eyez	girlies	mosquitoes	joints	tix	tamales
babez	sideburns	twamily	turkeys	hitters	gifts	wedges
bae	jammies	tweeples	tt's	costumes	tixs	gumbo
bruhh	talents	twiggas	tts	emojis	invites	crawfish
bby	grades	tweeps	c's	leggins	suggestions	blueberries
ddub	cockiness	peeps	midterms	laptops	remedies	smoothies
bbs	butthole	tweethearts	sigmas	gloves	takers	pumpkin
tete	momz	twitterville	professors	cleats	predictions	chowder
bb	cuzzin	tweeties	earthquakes	trunks	nites	cherries
tunechi	novio	lovies	kiddies	grenades	niight	casserole
todos	bestfriend	twitterland	deltas	lighters	nyte	ice-cream
babii	homeboi	hunnies	bats	trax	nite	barbecue
lul	cuzzos	sorors	subliminals	snuggies	nitee	sandwhich

Word list: Page 4 of 6.

canes
bbq
locos
turkey
veg
chilli
watermelon
ceasar
choc
frap
frappe
butta
smores
popsicles
potatoe
pina
chix
pneumonia
bronchitis
libra
terrorism
bullying
tsunami
qualifying
3-d
detention
3d
hd
trainin
threes
persuasion
nightlife
defense
amusement
comm
psychology
accounting
anatomy
mojito
chardonnay
merlot
moscato
eggnog
joose
martini
cider
patron
liqour
mojitos
latte
nuvo
lipgloss
guestlist
sunscreen
lint
radiation
bandwidth
drilling
tide
h20
randomness
autotune
climax
sunburn
applause
thirst
warmth
capitalism
ambition
ridiculousness
sucess
snow
pollen
sleet
flurries
thunder
lightening
thunderstorm
s
storms
willies
fog
leopard
showers
perf
ratchetness
goody
coonery
fiyah
shiznit
bitchassness
bullshyt
piff
lrt
ping
autocorrect
twitted
twitterr
deck
campus
racks
hoarders
rss
dsl
a/c
apocalypse
physics
calc
astronomy
sociology
calculus
omega
geometry
algebra
math
pc
psych
thesis
chem
quince
biology
creole
lte
tablet
froyo
jailbroken
desktop
gametime
bfast
poolside
tailgating
fishing
sledding
swimming
golfing
h.a.m
snowboarding
biking
shoppin
skiing
tanning
bowlin
fishin
swimmin
clubbin
kayaking
camping
tubing
kickback
potluck
cookout
bane
begining
weeknd
wrld
abyss
motto
creator
miz
crazies
buzzer
sideline
ballpark
caf
hosp
sidelines
dancefloor
beachh
moviess
bookstore
supermarket
pool
bachelor
endzone
studio
dugout
carwash
in-laws
fireplace
holidays
internets
library
patio
syllabus
buildin
itis
bizness
grinch
wackness
juiceman
porch
sunroof
bizz
lawn
krib
babyshower
dorm
stepfather
mamba
sleigh
crackberry
comp
trampoline
vm
router
trackball
bberry
fone
fne
fones
backround
avi
icon
avii
twiticon
avatar
background
acct
acc
layout
default
twitcon
gamertag
namee
tethering
firmware
widget
api
browser
site
homepage
plugin
earthquake
assassin
umpire
outburst
essay
e-mail
mms
addy
spam
interception
emoji
allergy
alchy
icee
wave
muzik
musik
bootleg
disk
webcam
batt
antenna
lab
modem
cpu
netbook
adapter
gift
promo
swagga
wasp
quickie
concussion
smut
tux
badger
peacock
slime
broom
bootz
reindeer
firework
tings
tt
snowball
snowman
cooter
mosquito
beeper
fitted
hipster
monsta
rockstar
jumpoff
ting
b+
lacefront
bikini
mink
guido
d*ck
choppa
professor
ump
prof
pin
fams
bottoms
ribbon
lite
coat
hoody
trench
cardigan
snapback
jacket
sweatshirt
gown
stylist
sundress
scarf
hoodie
v-neck
sweater
hammock
mound
parade
maze
licker
cabin
tree
recession
volcano
storm
breeze
resturant
swimsuit
thunderstorm
banga
tornado
shutout
fumble
grenade
layup
lapdance
touchdown
homerun
showerr
spliff
salts
texter
snippet
screenshot
vid
hitter
3some
mixtape
cypher
joint
sextape
sonq
bullpen
playoffs
tournament
matchup
lockout
redzone
ballgame
topic
af
texters
a'f
asf
mgr
coordinator
commerical
pact
forum
prototype
sidebar
nomination
cache
flyer
software
wiki
scandal
portfolio
database
newsletter
interference
savings
decor
elections
svc
processor
panels
outage
rankings
themes
supplies
workflow
developer
tutoring
documentatic
n
conversion
exp
integration
ui
interface
auditorium
bldg
blitz
conditioning
heater
fest
festival
klub
medal
computing
disasters
bases
scorer
beatdown
afterparty
presentation
banquet
sesh
gala
conf
keynote
tweetup
photoshoot
webinar
ceremony
finale
demo
premiere
catalog
opener
photog
kicker
goalie
quarterback
bracket
receiver
o-line
pitcher
officiating
qb
dictator
promoter
hoax
client
vaccine
producer
refill
study
setup
plank
file
rebound
dunk
lecture
scrimmage
turnover
protest
check-in
revolt

Word list: Page 5 of 6.

tailgate	low-key	foggy	rnb	nighty
rally	quik	humid	kick-ass	nitey
timeout	krazy	snowy	burlesque	lst
shootout	wackk	coldd	horror	nxt
wrkout	cray	muggy	snowboard	nex
kickoff	crazi	chilly	glam	farmer's
tutor	crazii	thundering	camp	awkward
retreat	crazzy	coold	dubstep	neww
meanin	crzy	freezin	sci-fi	wittle
scope	smoove	tamed	hiphop	biig
launch	nutz	swizz	lotus	biq
bundle	fancy	linen	harvest	teeny
disclaimer	wack	flannel	mvp	24hr
s/n	trill	camo	hip-hop	womans
verdict	trippy	suede	westcoast	meteor
ques	stoopid	pajama	soca	uur
url	wreckless	fleece	vamp	otha
vow	wak	jingle	fg	othr
hashtag	grimey	swamp	bronze	2c
tribute	judgmental	gingerbread	shark	t2
refund	rediculous	blck	lightning	tew
meetin	annoyin	blk	lib	2b
mtg	borin	haunted	football	2da
qtr	awk	geaux	pre-season	2the
inning	embarassing	twitterless	crossfit	2do
quater	spooky	balloon	b-ball	2get
warranty	hopeless	atta	triathlon	ths
resolution	debatable	olympic	allstar	yesterdays
costume	irrelevant	women's	frisbee	anotha
roulette	hala	iconic	spades	anutha
decorations	hillarious	unsung	gameday	evry
resolutions	unforgettable	womens	lacrosse	a$ap
gully	amazin	mens	multiplayer	n0
dutty	halarious	upscale	preseason	
wavy	truue	celeb	soccer	
trendy	truu	freelance	baseball	
matchin	nicee	prepaid	varsity	
ratchet	kewl	wireless	golf	
sexii	niccee	touchscreen	pep	
rachet	hott	seasonal	fball	
dtf	kute	mindless	vball	
beastly	gudd	xtra	f1	
funnel	gd	external	softball	
peppermint	qood	batting	derby	
corned	guud	static	playoff	
tanned	gr8	curved	volleyball	
bizzy	grreat	mp3	ski	
chapped	kickass	torrent	kickball	
collard	niice	pdf	postseason	
clutch	doobie	rbi	stimulus	
intresting	precious	offical	healthcare	
interestin	2much	infamous	hc	
ode	betta	domain	voter	
ez	bettr	dr's	immigration	
prosperous	bettah	parkin	reform	
diff	colder	secondary	web	
dif	warmer	grad	cust	
seperate	hotter	flu	biz	
ppl's	sharper	quake	gadget	
subliminal	lonq	couture	baskets	
ppls	tardy	farmers	skating	
winky	windy	rack	quiz	
foul	dreary	powerhouse	overcast	
futuristic	cold	renegade	wackest	
shameless	rainy	xxl	flyest	
festive	freezing	blogger	deadliest	

Word list: Page 6 of 6.

References

1. Owoputi O, O'Connor B, Dyer C, Gimpel K, Schneider N, et al. (2013) Improved part-of-speech tagging for online conversational text with word clusters. In: Proceedings of the Conference of the North American Chapter of the Association for Computational Linguistics.
