# Supplementary material for: Diffusion of Lexical Change in Social Media
Source: PLoS One. 2014 Nov 19;9(11):e113114. doi: 10.1371/journal.pone.0113114 (PMC4237389; doi:10.1371/journal.pone.0113114)
Supplement: File S1 — Appendix S1-S3, Table S1 and Software S1. Appendix S1. Term list. List of all words considered in our main analysis. Appendix S2. Term examples. Examples for each term considered in our analysis. Appendix S3. Data Procedures. Description of the procedures used for data processing, including Twitter data acquisition, geocoding, content filtering, word filtering, and text processing. Table S1. Term annotations. Tab-separated file describing annotations of each term as entities, foreign-language, or acceptable for analysis. Software S1. Preprocessing software. Source code for data preprocessing. (ZIP) [file pone.0113114.s001.zip › supp_for_upload/Appendix_S2_Term_Examples.html]

## bendiciones

1. @luuzzhhyy
   you
   welcome
   !!
   gracias
   ..
   eres
   muy
   linda
   !!
   gracias
   por
   tus
   lindas
   **bendiciones**
   :)
   claro
   igual
   para
   ti
   !
   mucha
   y
   lindas
   **bendiciones**- @neidasandoval
     buenos
     y
     hernosos
     dia
     para
     ti
     mi
     apreciada
     neida
     .
     recibe
     muicjas
     **bendiciones**
     y
     energia
     .- saludos
       ,
       gracias
       por
       tu
       apoyo
       y
       **bendiciones**
       ,
       @anyelin\_v
       @yanethamado- felices
         fiestas
         ..
         que
         disfruten
         de
         la
         compa
         ~
         ia
         de
         los
         suyos
         !!
         muchisimas
         **bendiciones**
         ..
         abrazos
         pa
         todos
         !!
         <3- gracias
           a
           mi
           familla
           y
           amigos
           x
           todas
           las
           **bendiciones**
           en
           my
           cumple
           !!..
           los
           quiero
           muchoo
           !!
           dios
           los
           bendiga
           !!
           xoxoxo
           :))
           <3
           <3
           <3- @chiquibabyla
             hello
             hello
             buen
             dia
             a
             la
             mas
             bella
             de
             la
             radio
             **bendiciones**- @emelinsosa
               **bendiciones**
               emelin
               todo
               bien
               por
               estos
               lados
               .
               espero
               que
               todo
               este
               bien
               por
               tu
               lado
               .
               como
               va
               lo
               de
               tu
               libro
               ?- @a\_arambula
                 hola
                 tocaya
                 ..
                 cuando
                 se
                 presentan
                 en
                 san
                 luis
                 rio
                 colorado
                 sonora
                 ..
                 haber
                 su
                 tu
                 puedes
                 regresarme
                 mi
                 tweeter
                 ,.
                 **bendiciones**- @thalia
                   te
                   queremos
                   mas
                   q
                   nunca
                   **bendiciones**
                   a
                   toda
                   la
                   familia- @lorenarojas
                     happy
                     birthday
                     lore
                     ,
                     que
                     te
                     la
                     pases
                     increible
                     ,
                     un
                     a^ito
                     mas
                     x
                     el
                     cual
                     hay
                     que
                     agradecerle
                     a
                     dios
                     .
                     muchas
                     **bendiciones**
                     .
                     xoxo- @linnafer
                       gracias
                       lina
                       !!
                       igualmente
                       !
                       lindo
                       fin
                       de
                       semana
                       !!
                       **bendiciones**
                       !- @natashajulyanne
                         igual
                         beba
                         q
                         tengas
                         1
                         dia
                         maravillosa
                         !!
                         exitos
                         y
                         **bendiciones**
                         !!- @charytinoficial
                           mi
                           chary
                           querida
                           ,
                           que
                           dios
                           te
                           llene
                           de
                           **bendiciones**
                           y
                           que
                           te
                           conserve
                           como
                           hasta
                           ahora
                           feliz
                           dia
                           ,
                           muchas
                           muchas
                           cosas
                           buenas- @angelicareynosa
                             gracias
                             !
                             estamos
                             muy
                             emocionados
                             !
                             **bendiciones**
                             !- @ivanagav
                               felicidades
                               en
                               un
                               dia
                               tan
                               hermoso
                               y
                               especial
                               para
                               ti
                               ..
                               que
                               las
                               **bendiciones**
                               continuen
                               llegando
                               y
                               el
                               exito
                               fluyendo
                               !
                               un
                               abrazo
                               -r.c- @jackybrv
                                 buenas
                                 noches
                                 jacky
                                 !!
                                 descansa
                                 !!
                                 que
                                 esta
                                 semana
                                 este
                                 llena
                                 de
                                 muchas
                                 alegrias
                                 y
                                 **bendiciones**
                                 !!
                                 love
                                 ya
                                 !!- espero
                                   de
                                   todo
                                   corazon
                                   que
                                   tengan
                                   un
                                   dia
                                   excelente
                                   y
                                   repleto
                                   de
                                   **bendiciones**
                                   !- @elenagarcia36
                                     @sirenitaa83
                                     oki
                                     doki
                                     yo
                                     les
                                     dejo
                                     saber
                                     como
                                     me
                                     salio
                                     ok
                                     !!
                                     besos
                                     y
                                     **bendiciones**
                                     .- **bendiciones**
                                       salmo
                                       86:11
                                       ensename
                                       ,
                                       oh
                                       jehova
                                       ,
                                       tu
                                       camino
                                       ;
                                       caminare
                                       yo
                                       en
                                       tu
                                       verdad
                                       .
                                       afirma
                                       mi
                                       corazon
                                       para
                                       que
                                       tema
                                       tu
                                       nombre
                                       .- linda
                                         noche
                                         y
                                         **bendiciones**
                                         a
                                         cada
                                         ser
                                         humano
                                         en
                                         el
                                         mundo
                                         ,
                                         good
                                         night
                                         att
                                         .
                                         los
                                         rivera

## bendiga

1. @singingbush
   gracias
   :)
   buen
   dias
   !
   feliz
   miercoles
   !
   dios
   te
   **bendiga**
   ,
   besos- @cochimundial
     yeyy
     que
     bueno
     los
     veo
     el
     lunes
     tambien
     que
     emocion
     ya
     lo
     quiero
     ver
     cantar
     ahy
     cochi
     dios
     los
     **bendiga**
     a
     los
     dos- @jennirivera
       hope
       ur
       feeling
       better
       diva
       y
       que
       diosito
       te
       **bendiga**
       cause
       ur
       such
       a
       blessing
       to
       all
       of
       us- @felipeviel
         omg
         i
         love
         this
         pic
         tus
         hijas
         bellas
         ke
         dios
         los
         **bendiga**- feliz
           dia
           mi
           gente
           !!
           que
           disfruten
           este
           sabado
           precioso
           q
           dios
           nos
           a
           dado
           !!
           que
           dios
           los
           **bendiga**- @ricky\_martin
             how
             precious
             is
             ur
             little
             man
             !!
             que
             dios
             los
             **bendiga**
             !
             un
             beso
             para
             el
             chiquito
             !- @chespiritorgb
               senoron
               ..
               dios
               me
               lo
               **bendiga**
               ..
               saludos
               desde
               new
               orleans
               ,
               louisiana
               usa
               ..
               super
               mega
               fan
               de
               ud
               !!- @laureanomar
                 amigo
                 acabo
                 de
                 leer
                 tu
                 columna
                 de
                 verdad
                 que
                 tienes
                 un
                 gran
                 corazon
                 !
                 dios
                 te
                 **bendiga**
                 !- buenos
                   dias
                   a
                   todos
                   q
                   pasen
                   en
                   lindo
                   dia
                   juntos
                   a
                   sus
                   seres
                   queridos
                   hoy
                   en
                   christmas
                   eve
                   dios
                   los
                   **bendiga**- @chiquibabyla
                     happy
                     birthday
                     te
                     deceo
                     lo
                     mejor
                     y
                     que
                     dios
                     te
                     **bendiga**
                     con
                     muchos
                     anos
                     mas
                     ,
                     y
                     gracias
                     por
                     la
                     foto
                     y
                     msgs
                     estan
                     lindas
                     xoxoxo- happy
                       easter
                       !
                       may
                       god
                       bless
                       you
                       and
                       your
                       loved
                       ones
                       on
                       this
                       day
                       !!
                       felices
                       pascuas
                       !
                       que
                       dios
                       te
                       **bendiga**
                       a
                       ti
                       y
                       a
                       los
                       tuyos
                       !
                       besos
                       !- no
                         lo
                         puedo
                         creer
                         k
                         ya
                         pasaron
                         2
                         meces
                         k
                         ya
                         no
                         estas
                         a
                         ki
                         con
                         nosotros
                         pero
                         ni
                         modo
                         k
                         dios
                         t
                         **bendiga**
                         t
                         amo
                         carnalito- @paola\_rojas\_h
                           muchas
                           felicidades
                           y
                           que
                           dios
                           los
                           **bendiga**
                           plenamente
                           .- @jazzeymarie
                             happy
                             new
                             year
                             god
                             bless
                             :
                             feliz
                             de
                             anos
                             y
                             dios
                             **bendiga**- good
                               night
                               un
                               dia
                               con
                               muchas
                               emociones
                               encontradas
                               pero
                               al
                               final
                               kon
                               buenos
                               resultados
                               k
                               descansen
                               i
                               love
                               @abnermares00
                               ..
                               dios
                               nos
                               **bendiga**
                               .- @lalolachanga
                                 ya
                                 esta
                                 compare
                                 saludos
                                 y
                                 dios
                                 lo
                                 **bendiga**- viendo
                                   "
                                   brothers
                                   "
                                   good
                                   nite
                                   ppl
                                   k
                                   dios
                                   me
                                   los
                                   **bendiga**
                                   .
                                   lmm- @jennirivera
                                     good
                                     night
                                     mi
                                     diva
                                     sweet
                                     dreams
                                     que
                                     dios
                                     te
                                     **bendiga**
                                     i
                                     love
                                     youu
                                     un
                                     chingoo
                                     !!- @christian\_pagan
                                       no
                                       soy
                                       d
                                       esas
                                       fans
                                       q
                                       t
                                       escriben
                                       a
                                       diario
                                       pero
                                       nunca
                                       olvido
                                       presentar
                                       tu
                                       vida
                                       delante
                                       de
                                       dios
                                       para
                                       q
                                       t
                                       **bendiga**
                                       grandemente
                                       !- happy
                                         birthday
                                         to
                                         my
                                         amigo
                                         @davideworship
                                         from
                                         peru
                                         !!
                                         1
                                         of
                                         our
                                         best
                                         translators
                                         for
                                         our
                                         peru
                                         mission
                                         trips
                                         .
                                         dios
                                         te
                                         **bendiga**
                                         !!
                                         c
                                         u
                                         soon
                                         !!

## bomb.com

1. card
   from
   grandmother
   was
   thick
   she
   the
   **bomb.com**- pops
     made
     lasagna
     tonite
     wowzers
     !
     **bomb.com**- @drnew\_new2u
       it
       was
       the
       **bomb.com**
       wasn't
       it
       ??- my
         bae
         tyga
         was
         **bomb.com**
         :
         )- my
           muvaa
           iss
           thee
           **bomb.com**- #isitjustme
             or
             do
             kfc
             chicken
             on
             the
             commercial
             b
             lookin
             **bomb.com**
             #fattweet- had
               the
               **bomb.com**
               brunch
               ..
               thx
               2
               urs
               truly
               !!
               hopefully
               th@
               can
               help
               lift
               me
               ..
               been
               a
               really
               bad
               effin
               day
               !- my
                 moms
                 lemon
                 pepper
                 boneless
                 bake
                 chicken
                 **bomb.com**- dinner
                   was
                   **bomb.com**
                   too
                   bad
                   there
                   wasn't
                   anyone
                   to
                   share
                   it
                   with- condo
                     bbq
                     jumping
                     ,
                     better
                     than
                     the
                     damn
                     club
                     ..
                     **bomb.com**- i
                       forgot
                       all
                       about
                       will
                       from
                       making
                       the
                       band
                       ,
                       but
                       this
                       nigga
                       songs
                       are
                       **bomb.com**- this
                         food
                         is
                         about
                         to
                         be
                         **bomb.com**
                         #tamar- @callmebre
                           thanks
                           boo
                           !!
                           that
                           added
                           ingredient
                           was
                           definitely
                           the
                           **bomb.com**- what
                             ceases
                             to
                             amaze
                             me
                             is
                             how
                             rats
                             truely
                             believe
                             that
                             their
                             rave
                             outfits
                             be
                             the
                             **bomb.com**
                             o\_o
                             .
                             u
                             can't
                             have
                             the
                             highest
                             confidence
                             ..
                             #no- that
                               meal
                               i
                               just
                               fixed
                               was
                               the
                               **bomb.com**- #ff
                                 @dreamhampton
                                 cuz
                                 u
                                 will
                                 fall
                                 in
                                 love
                                 with
                                 her
                                 as
                                 i
                                 did
                                 .
                                 she's
                                 the
                                 **bomb.com**- "
                                   milk
                                   and
                                   cookies
                                   "
                                   **bomb.com**- @justincunt
                                     i'd
                                     make
                                     you
                                     a
                                     **bomb.com**
                                     breakfast
                                     !- @juniorniteout
                                       woop
                                       woop
                                       !
                                       ur
                                       the
                                       **bomb.com**
                                       :)- heey
                                         @ramii\_ram
                                         is
                                         on
                                         my
                                         tl
                                         !
                                         #ff
                                         she's
                                         the
                                         **bomb.com**

## dejar

1. @laranita25
   jjaja
   ,
   si
   me
   gusta
   el
   movimiento
   en
   twitter
   !
   pero
   dificilmente
   podria
   **dejar**
   facebook
   !- @howtwofuckup
     sabes
     lo
     que
     me
     pasa
     ?
     que
     hace
     rato
     no
     hablas
     conmigo
     ,
     pero
     yo
     te
     voy
     a
     **dejar**
     ,
     sooner
     or
     later
     you're
     gonna
     need
     me
     .- @januick
       lol
       es
       k
       cada
       ves
       k
       se
       acaba
       te
       **dejar**
       con
       el
       deseo
       de
       saber
       k
       va
       pasar
       ..- un
         #ff
         para
         @vladdo
         porque
         nuncamente
         me
         va
         a
         **dejar**
         de
         querer
         !- voy
           a
           **dejar**
           un
           "
           me
           encantas
           "
           ,
           digo
           por
           si
           me
           stalkeas
           !
           .\_.- basta
             !
             tengo
             que
             **dejar**
             de
             pensar
             en
             el
             !!- #yoconfieso
               que
               hoy
               me
               quiero
               **dejar**
               querer
               ..
               (
               via
               @mariurdaneta
               )
               <--
               despues
               de
               confesar
               que
               rico
               los
               mensajes
               de
               amor
               ..
               gracias
               funciono
               !- @predikador
                 gracias
                 x
                 **dejar**
                 el
                 nombre
                 de
                 panama
                 en
                 alto
                 ,
                 muy
                 orgullosa
                 de
                 tu
                 triunfos
                 ,
                 los
                 panamenos
                 te
                 hemos
                 visto
                 crecer- "
                   divide
                   and
                   conquer
                   "
                   es
                   lo
                   que
                   esta
                   haciendo
                   el
                   crimeen
                   organizado
                   ..
                   no
                   hay
                   que
                   **dejar**
                   que
                   nos
                   dividan
                   ..
                   hay
                   que
                   estar
                   mas
                   unidos
                   que
                   nunca- :
                     $
                     :
                     $
                     no
                     pude
                     **dejar**
                     de
                     mirarla
                     hoy
                     x\_x
                     qe
                     me
                     pasa
                     ?
                     :
                     $- que
                       bo
                       ye
                       pase
                       **dejar**
                       las
                       compras
                       en
                       el
                       bano
                       que
                       lastima
                       que
                       era
                       li
                       de
                       @estefibaeza
                       :(
                       ahora
                       te
                       tendre
                       quw
                       comprar
                       otra
                       cosa- por
                         mas
                         q
                         quiera
                         **dejar**
                         de
                         pensar
                         las
                         cosas
                         no
                         puedo- @lemirsale
                           then
                           this
                           bitch
                           ass
                           nigga
                           gonna
                           at
                           me
                           some
                           bullshit
                           in
                           spanish
                           slim
                           i'm
                           abt
                           to
                           **dejar**
                           de
                           seguir
                           this
                           dude
                           man
                           ..- que
                             tengo
                             que
                             hacer
                             pa
                             que
                             vuelvas
                             conmigo
                             vamos
                             a
                             **dejar**
                             el
                             pasado
                             atras
                             !- antibioticos
                               sera
                               que
                               me
                               van
                               a
                               **dejar**
                               recordar
                               la
                               noche
                               capitalina
                               (
                               o
                               lo
                               que
                               queda
                               de
                               ella
                               ?
                               )- si
                                 tenia
                                 nostalgia
                                 x
                                 **dejar**
                                 #nyc
                                 ahora
                                 la
                                 tengo
                                 pero
                                 elevada
                                 a
                                 la
                                 5ta
                                 potencia
                                 por
                                 **dejar**
                                 #miami- @jmacjohis
                                   @tvn\_amaro
                                   @villatri
                                   asi
                                   son
                                   estos
                                   encuentros
                                   .
                                   sirven
                                   para
                                   **dejar**
                                   una
                                   deuda
                                   al
                                   pais
                                   anfitrion
                                   y
                                   1
                                   q
                                   otro
                                   avance
                                   en
                                   las
                                   bilaterales
                                   .- decidi
                                     **dejar**
                                     de
                                     ser
                                     puntual
                                     ,
                                     cuando
                                     el
                                     tiempo
                                     decidio
                                     portar
                                     un
                                     reloj
                                     #juevesfilosofico- @yosoy\_elvitor
                                       tan
                                       intenso
                                       que
                                       no
                                       me
                                       vas
                                       a
                                       **dejar**
                                       ir
                                       ni
                                       un
                                       momento
                                       :-)
                                       :-)- que
                                         pensaban
                                         al
                                         **dejar**
                                         que
                                         miley
                                         cyrus
                                         se
                                         paseara
                                         por
                                         el
                                         c.c
                                         el
                                         recreo
                                         ?
                                         se
                                         pasaron
                                         ,
                                         de
                                         pana
                                         .

## deseo

1. @cristianugh2011
   hola
   saludos
   a
   los
   dos
   ,
   estoy
   ree
   feliz
   estoy
   mirando
   pm
   les
   **deseo**
   lo
   mejor
   en
   la
   vida
   su
   fans
   de
   las
   vegas- el
     **deseo**
     es
     tanto
     que
     quema
     por
     dentro
     .
     @tierranightclub- @lainsuperable06
       happy
       birthday
       te
       **deseo**
       lo
       mejor
       de
       lo
       mejor
       disfruta
       tu
       birthday- a
         todos
         los
         que
         a
         diario
         me
         leen
         les
         **deseo**
         lo
         mejor
         en
         este
         2010
         .
         estoy
         con
         un
         gripon
         no
         he
         dormido
         me
         acabo
         de
         tomar
         algo
         que
         causa
         sueno- @manedelaparra
           iguamente
           para
           ti
           !
           y
           desde
           ya
           los
           mejores
           **deseo**
           pa
           el
           2011
           lleno
           de
           cosas
           lindas
           para
           ti
           y
           tu
           gente
           y
           pa
           dond
           vas
           de
           vacas
           ?- tengo
             **deseo**
             de
             ir
             a
             un
             party
             en
             casa
             y
             baila
             do
             dembow
             ,
             solamente
             do
             ..
             i
             miss
             dr
             so
             fucking
             much
             !
             ugh
             !- whatever
               man
               @3gerardpique
               is
               officially
               my
               twitter
               prince
               solo
               **deseo**
               que
               sea
               mi
               principe
               en
               realidad
               <3
               \_\_
               <3- @jlbarcenasm
                 te
                 **deseo**
                 una
                 linda
                 semana
                 !- @nanenina88
                   btw
                   felicidades
                   en
                   tu
                   grad
                   !
                   te
                   **deseo**
                   muchas
                   cosas
                   buenas
                   en
                   el
                   futuro
                   !
                   tienes
                   q
                   ir
                   a
                   visitar
                   el
                   semestre
                   que
                   viene
                   !- @ponchohd
                     no
                     entiendo
                     que
                     tiene
                     que
                     ver
                     10/10/10
                     con
                     un
                     **deseo**
                     ?
                     me
                     lo
                     puedes
                     explicar
                     please
                     ?
                     aqui
                     en
                     ca
                     son
                     las
                     8:45
                     am
                     .?
                     gracias- si
                       supieran
                       cuanto
                       **deseo**
                       mudarme
                       pronto
                       a
                       vivir
                       solo
                       !
                       no
                       tienen
                       idea
                       !
                       :/
                       quiero
                       hacer
                       mi
                       vida
                       ya
                       !!- si
                         hoy
                         pudiera
                         pedir
                         un
                         **deseo**
                         es
                         que
                         rooney
                         se
                         arrepienta
                         ,
                         se
                         disculpe
                         con
                         ferguson
                         ,
                         su
                         equipo
                         y
                         la
                         aficion
                         ,
                         y
                         regrese
                         ese
                         crack- alguien
                           tiene
                           motivo
                           (
                           **deseo**
                           de
                           poder
                           )
                           ,
                           tiene
                           oportunidad
                           (
                           portavoz
                           con
                           movil
                           de
                           tarjeta
                           )
                           y
                           obtiene
                           beneficio
                           (
                           adhesion
                           incondicional
                           )- @jeanius000
                             felizidades
                             hermano
                             te
                             **deseo**
                             lo
                             mejor
                             besos
                             y
                             abrazos
                             y
                             muchas
                             bendiciones
                             para
                             delante
                             para
                             atras
                             ni
                             para
                             cojer
                             impurso- para
                               ti
                               es
                               amor
                               duenia
                               de
                               tu
                               corazon
                               ,
                               para
                               mi
                               es
                               solo
                               un
                               **deseo**
                               ..
                               no
                               hay
                               q
                               ser
                               muy
                               listo
                               para
                               darse
                               cuenta
                               q
                               ella
                               es
                               un
                               camuflaje- @johansantana
                                 te
                                 **deseo**
                                 mucha
                                 salud
                                 y
                                 que
                                 venga
                                 ayudarnos
                                 rapidamente
                                 en
                                 esta
                                 temporada
                                 .
                                 #mets- **deseo**
                                   que
                                   cualquier
                                   anhelo
                                   o
                                   meta
                                   que
                                   tenga
                                   el
                                   @bbvaprovincial
                                   se
                                   haga
                                   relidad
                                   #bbvaadelante- hay
                                     deseos
                                     a
                                     largo
                                     plazo
                                     ,
                                     por
                                     ello
                                     **deseo**
                                     que
                                     su
                                     excelencia
                                     dure
                                     hasta
                                     siempre
                                     @bbvaprovincial
                                     #bbvaadelante- @vanessapalomino
                                       hola
                                       vanesita
                                       te
                                       mando
                                       muchicimos
                                       saludos
                                       .
                                       y
                                       te
                                       **deseo**
                                       un
                                       bonito
                                       fin
                                       de
                                       semana
                                       .- no
                                         creo
                                         en
                                         la
                                         suerte
                                         ,
                                         pero
                                         si
                                         en
                                         el
                                         exito
                                         y
                                         de
                                         este
                                         te
                                         **deseo**
                                         muchisimoo
                                         !
                                         besos
                                         @pepebarbosa44

## equipo

1. "
   este
   **equipo**
   tiene
   que
   recuperar
   el
   lugar
   que
   le
   corresponde
   en
   la
   historia
   "
   :
   miguel
   herrera- @cotofdp
     @dubertialejo
     yoo
     t
     he
     ayudadoo
     !..
     y
     t
     voy
     a
     seguir
     ayudando
     !..
     como
     siempre
     me
     pongo
     el
     **equipo**
     al
     hombroo- @alejo1986sc
       paciencia
       ,
       galeno
       !
       ahora
       es
       que
       arranca
       la
       temp
       .
       el
       milan
       golea
       a
       un
       **equipo**
       de
       serie
       c
       y
       ya
       quieres
       criticar
       a
       los
       demas
       jajaja- @desafiocaracol
         sii
         ,
         se
         lo
         merecian
         los
         3
         en
         cambio
         en
         el
         otro
         **equipo**
         hay
         varios
         que
         no
         merecen
         llegar
         a
         una
         fusion
         :)- que
           bien
           esta
           jugando
           el
           milan
           vale
           ..
           el
           **equipo**
           esta
           dandole
           una
           buena
           despedida
           y
           agradecimiento
           a
           leonardo
           ..- @oscardavidguga
             jajaja
             pq
             no
             lo
             admites
             ?
             tu
             **equipo**
             gana
             gracias
             a
             sus
             goles
             .
             real
             madrid
             papa
             !- la
               diferencia
               de
               nosotros
               con
               los
               madridistas
               es
               q
               nosotros
               si
               nos
               sentimos
               orgullosos
               de
               la
               actuacion
               de
               nuestro
               **equipo**
               !- @kvca
                 que
                 madre
                 haber
                 estado
                 en
                 el
                 **equipo**
                 de
                 debate
                 verdad
                 ?
                 ^rm- si
                   tu
                   **equipo**
                   es
                   #cruzazul
                   somos
                   el
                   lider
                   general
                   #vamosazul- @radio102nueve
                     con
                     respeto
                     a
                     cheyo
                     pero
                     por
                     bolo
                     no
                     ha
                     dado
                     el
                     ancho
                     en
                     ningun
                     **equipo**
                     de
                     afuera
                     no
                     puedo
                     verlo
                     como
                     role
                     model- @foxsincodigos
                       vergara
                       dijo
                       entregar
                       un
                       ferrari
                       a
                       quirarte
                       y
                       le
                       devolvio
                       un
                       vocho
                       ,
                       vergara
                       recibio
                       un
                       **equipo**
                       de
                       1-
                       div
                       y
                       devuelve
                       un
                       infantil- oficialmente
                         jonathan
                         dos
                         santos
                         ya
                         es
                         parte
                         del
                         primer
                         **equipo**
                         del
                         barcelona
                         suerte
                         cabron
                         !!- dakjsldkjldkjlas
                           el
                           **equipo**
                           saco
                           el
                           fuaa- y
                             hoy
                             camino
                             al
                             trabajo
                             recuerden
                             .
                             si
                             ,
                             es
                             lunes
                             .
                             si
                             ,
                             que
                             verga
                             .
                             pero
                             al
                             menos
                             el
                             **equipo**
                             es
                             finalista
                             !..
                             que
                             pena
                             por
                             los
                             emelecistas
                             hoy
                             :(- por
                               q
                               dicen
                               el
                               **equipo**
                               de
                               dal
                               de
                               dirk
                               contra
                               el
                               **equipo**
                               de
                               mia
                               de
                               lebron
                               !
                               sres
                               la
                               estrella
                               del
                               heat
                               es
                               wade
                               !!- ira
                                 si
                                 ni
                                 como
                                 apoyar
                                 a
                                 mi
                                 **equipo**
                                 mm
                                 es
                                 un
                                 pinche
                                 asco
                                 no
                                 meterlos
                                 ya
                                 es
                                 normal
                                 para
                                 ellos
                                 pero
                                 q
                                 pinche
                                 defensa- si
                                   un
                                   **equipo**
                                   de
                                   futbol
                                   ,
                                   no
                                   yo
                                   .\_.- por
                                     q
                                     dicen
                                     el
                                     **equipo**
                                     de
                                     dal
                                     de
                                     dirk
                                     contra
                                     el
                                     **equipo**
                                     de
                                     mia
                                     de
                                     lebron
                                     !
                                     sres
                                     la
                                     estrella
                                     del
                                     heat
                                     es
                                     wade
                                     !!- no
                                       estamos
                                       concentrado
                                       en
                                       lo
                                       que
                                       hablan
                                       su
                                       mierda's
                                       ,
                                       me
                                       dijeron
                                       que
                                       algo
                                       bien
                                       hace
                                       tu
                                       y
                                       tu
                                       **equipo**
                                       para
                                       que
                                       esten
                                       hablando
                                       de
                                       ellos
                                       @djmemo- @jona2santos
                                         jugaste
                                         bien
                                         pero
                                         a
                                         veces
                                         viene
                                         bien
                                         una
                                         derrota
                                         no
                                         todo
                                         estuvo
                                         mal
                                         esperamos
                                         q
                                         jio
                                         ya
                                         juegue
                                         en
                                         otro
                                         **equipo**

## esperar

1. puedo
   **esperar**
   a
   visitar
   nueva
   york
   este
   verano
   ,
   muy
   emocionado
   !- no
     puedo
     dejarte
     de
     amar
     ,
     y
     no
     puedo
     dejar
     de
     **esperar**
     ,
     y
     no
     puedo
     perderte
     al
     final
     ,
     y
     no
     te
     puedo
     olvidar
     .
     no
     se
     vivir
     si
     no
     estas- @justmad
       debimos
       haber
       sido
       doctores
       ,
       te
       hacen
       **esperar**
       1
       hora
       y
       te
       cobran
       $800
       por
       15
       mins
       .- @guto\_sena
         blz
         entao
         vamos
         **esperar**
         para
         agilizarmos
         kk- @conniehoneyhope
           tienes
           que
           **esperar**
           a
           que
           llegue
           alla
           :
           $- las
             guitarras
             quedaron
             alucinantes
             !
             solo
             faltan
             bajo
             y
             voces
             y
             listos
             para
             la
             mezcla
             .
             no
             puedo
             **esperar**
             para
             que
             la
             escuchen
             !- @gilmacarlina
               tengo
               q
               arreglar
               algunos
               papeles
               immigracion
               y
               eso
               ..
               y
               **esperar**
               ..
               la
               playa
               y
               el
               sol
               no
               ta
               mal
               ..
               me
               das
               un
               bistazo
               del
               brochure
               ?- el
                 amor
                 es
                 una
                 maravilla
                 ..
                 lo
                 tienes
                 que
                 **esperar**
                 .
                 <3- no
                   puedo
                   **esperar**
                   para
                   verte
                   ,
                   gracias
                   por
                   estar
                   en
                   mi
                   vida
                   !
                   tu
                   sabes
                   que
                   te
                   la
                   sabes
                   !- ahh
                     quiero
                     pasar
                     ya
                     que
                     ladilla
                     **esperar**- necesito
                       cafeina
                       !
                       lo
                       peor
                       de
                       la
                       vida
                       **esperar**
                       a
                       los
                       doctores
                       !
                       tengo
                       suenio
                       :(- @angelgabriel20
                         termina
                         de
                         decirselo
                         ,
                         para
                         que
                         **esperar**
                         mas
                         ?
                         :p- la
                           dicha
                           de
                           la
                           vida
                           consiste
                           en
                           tener
                           siempre
                           algo
                           que
                           hacer
                           ,
                           alguien
                           a
                           quien
                           amar
                           y
                           alguna
                           cosa
                           que
                           **esperar**
                           .
                           @chayannemusic- no
                             **esperar**
                             lo
                             inesperado
                             que
                             lo
                             inesperado
                             se
                             la
                             esperaba
                             ..?
                             #yosolomeentiendo- amor
                               mutante
                               amigos
                               con
                               derecho
                               y
                               sin
                               derecho
                               de
                               tenerte
                               siempre
                               y
                               siempre
                               tengo
                               que
                               **esperar**
                               paciente
                               el
                               pedazo
                               que
                               me
                               toca
                               de
                               ti- e
                                 aprendamos
                                 a
                                 **esperar**
                                 no
                                 senhor
                                 !
                                 enquanto
                                 esperamos
                                 ,
                                 que
                                 nos
                                 deleitemos
                                 nele
                                 ,
                                 contemplando
                                 sua
                                 face
                                 ..!- tiempo
                                   perdido
                                   ,
                                   bueno
                                   que
                                   mas
                                   tocara
                                   **esperar**
                                   y
                                   tener
                                   paciencia
                                   ..
                                   :/- @romivavi
                                     tampoco
                                     esta
                                     mal
                                     jajaja
                                     .
                                     no
                                     puedo
                                     **esperar**
                                     a
                                     que
                                     acabe
                                     el
                                     paron
                                     ,
                                     pero
                                     creo
                                     que
                                     si
                                     es
                                     como
                                     la
                                     pasada
                                     temporada
                                     ,
                                     hasta
                                     2013
                                     nada
                                     ..- odio
                                       **esperar**
                                       :/- no
                                         sabia
                                         que
                                         se
                                         podia
                                         tener
                                         2
                                         ganadores
                                         en
                                         guerrero
                                         .
                                         bueno
                                         de
                                         guerrero
                                         ya
                                         no
                                         se
                                         que
                                         **esperar**
                                         .

## gooaall

1. jamaica
   score
   **gooaall**
   !- **gooaall**
     !!
     captain
     !!
     #coyg- **gooaall**
       **gooaall**
       **gooaall**
       **gooaall**
       ..!!- **gooaall**
         !!- **gooaall**
           .
           #usa
           .
           #suckitlalas- **gooaall**
             !!
             donovan
             is
             a
             beast
             !- **gooaall**
               !!- **gooaall**
                 #uswnt- italy
                   just
                   scored
                   **gooaall**- @margermusic
                     the
                     vip
                     room
                     dj
                     :
                     dimarco
                     the
                     great
                     orchestrating
                     live
                     tonight
                     @aerobar
                     on
                     south
                     beach
                     ,
                     say
                     my
                     name
                     at
                     the
                     door
                     !
                     **gooaall**
                     !!- **gooaall**
                       !!
                       u-s-a
                       !!
                       u-s-a
                       !!
                       u-s-a
                       !!
                       u-s-a
                       !!
                       u-s-a
                       !!- **gooaall**- **gooaall**
                           !!- my
                             home
                             is
                             full
                             of
                             **gooaall**
                             screaming
                             .
                             go
                             usa
                             futbol
                             !- **gooaall**
                               paul
                               crowder- **gooaall**
                                 !!
                                 #gomez- **gooaall**
                                   argentina
                                   !!- **gooaall**
                                     !!
                                     :-d
                                     :-d
                                     :-d
                                     #briere
                                     #flyers
                                     #nhl
                                     #playoffs- **gooaall**
                                       ac
                                       milan
                                       !- **gooaall**
                                         !!
                                         keep
                                         it
                                         up
                                         germany
                                         !

## gooll

1. **gooll**
   arriba
   getafe
   !- **gooll**
     ..
     huevos
     cerrotes
     ..
     lol- #halamadrid
       **gooll**- **gooll**
         !!
         la
         furia
         !!- **gooll**
           de
           mexico
           ..
           gille
           !!- **gooll**
             de
             pumas- **gooll**
               !!
               kembali
               terkucilkan
               ,
               inter
               akan
               selalu
               antusiasi
               dlm
               menghadapi
               trick
               permainan
               roma
               ;p
               #ironis- **gooll**
                 !- yyeess
                   !!
                   never
                   give
                   up
                   ,
                   never
                   surrender
                   !!
                   #usmnt
                   **gooll**
                   lando
                   !!- uhuu
                     **gooll**
                     grande
                     @fabbrojony17- **gooll**
                       hijos
                       de
                       la
                       serpiente
                       #fb- **gooll**
                         chucha
                         **gooll**
                         #river- **gooll**
                           guardado
                           !!- @tourex
                             **gooll**
                             (
                             how
                             do
                             you
                             shoot
                             like
                             that
                             )
                             smh- **gooll**
                               !!- **gooll**
                                 de
                                 los
                                 negritos
                                 todo
                                 choco
                                 brinca
                                 de
                                 emocion
                                 por
                                 su
                                 raza
                                 !!
                                 #wc2010- **gooll**
                                   maravilha
                                   falta
                                   1
                                   pra
                                   acabar
                                   e
                                   2
                                   pra
                                   ficar
                                   tranquiloo- **gooll**
                                     !- **gooll**
                                       !!
                                       pedro
                                       !!- porfin
                                         **gooll**

## hheeyy

1. @ohhsoglamorous
   **hheeyy**
   wasup
   !!
   thanx
   for
   tha
   follow
   ..
   watchn
   106
   ugh
   so
   incredibly
   lame- been
     getting
     a
     lot
     of
     new
     followers
     ..
     **hheeyy**
     yyaall- @pecantan89
       **hheeyy**
       ..
       girl
       nothin
       ..
       tryna
       enjoy
       these
       last
       couple
       days
       before
       classes
       start
       !!
       u
       ??- **hheeyy**
         new
         follow
         !!
         talk
         to
         me
         so
         i
         can
         follow
         back
         :)- @d\_mabry10
           **hheeyy**- power
             !
             **hheeyy**- @devoted2luvinme
               **hheeyy**- @dha\_total\_pkg
                 **hheeyy**
                 boo- @sammyseaton03
                   @jake\_laing
                   **hheeyy**
                   wwee
                   wanntt
                   soomme
                   puussy
                   !- @yehpnohands
                     **hheeyy**
                     fool
                     !!- @kingwole
                       **hheeyy**- @barryarchie
                         lol
                         ,
                         **hheeyy**
                         barry
                         !- @dyme\_divazprod
                           **hheeyy**
                           bbaabbyy- @eddasays
                             **hheeyy**
                             !!- @miss\_bajan
                               **hheeyy**
                               yea
                               girl
                               iam
                               back
                               for
                               a
                               few
                               !
                               lol- @dru512
                                 **hheeyy**
                                 druu
                                 !!- @nysweetie155
                                   **hheeyy**
                                   tam- @punjaab\_huh
                                     **hheeyy**
                                     lol- @poliedagreat83
                                       aaww
                                       **hheeyy**
                                       i
                                       missed
                                       u
                                       !!
                                       i
                                       don't
                                       like
                                       your
                                       new
                                       friend
                                       tho
                                       #thatsall
                                       lol- @grinding4ddub
                                         **hheeyy**
                                         jjaaiimmee

## leeggoo

1. @dwiard11
   i'm
   so
   ready
   for
   college
   football
   season
   .
   tailgating
   ,
   coozies
   ,
   and
   kentucky
   bourbon
   !
   **leeggoo**
   :)
   hahaha- @ptownmiked
     **leeggoo**
     ..
     aye
     @jtee14
     n
     @lildaddy757
     we
     got
     some
     new
     victims
     in
     #2k
     ..- almost
       at
       that
       300
       mark
       !!
       **leeggoo**
       !!- **leeggoo**- #glee
           started
           the
           brittany
           episode
           **leeggoo**- leggo
             performing
             live
             in
             vergas
             newxt
             weekend
             ima
             tear
             the
             roof
             off
             tht
             shit
             **leeggoo**
             niga
             #s.o.m.e- basketball
               game
               at
               43o
               **leeggoo**- @monia\_lisa
                 **leeggoo**- @rainmillions
                   \*
                   singing
                   \*
                   i'm
                   going
                   back
                   to
                   cali
                   to
                   cali
                   i'm
                   going
                   back
                   to
                   cali
                   to
                   cali
                   ..
                   lol
                   .
                   619
                   skyline
                   park
                   !
                   **leeggoo**
                   !- @iam\_hollywood
                     wrd
                     !
                     yea
                     maann
                     im
                     already
                     on
                     !
                     soo
                     **leeggoo**- tweetin
                       from
                       the
                       web
                       !!
                       **leeggoo**
                       #lametweet- s/o
                         to
                         #mynewfollowers
                         u
                         guys
                         r
                         in
                         for
                         some
                         #somecrazyexoticclassyniggashit
                         **leeggoo**- just
                           got
                           that
                           call
                           "
                           mr.
                           hinson
                           ur
                           bookd
                           are
                           waiting
                           for
                           you
                           at
                           the
                           front
                           office
                           "
                           **leeggoo**
                           !- #soultrainawardweek
                             #coredjsretreat
                             **leeggoo**
                             !!
                             s/0
                             to
                             the
                             big
                             homie
                             tony
                             neal
                             !
                             congrats
                             bro
                             !
                             looking
                             forward
                             to
                             2night
                             !
                             @thecoredjs- **leeggoo**- i
                                 hope
                                 the
                                 #heat
                                 can
                                 close
                                 it
                                 out
                                 today
                                 .
                                 **leeggoo**
                                 !- got
                                   up
                                   ,
                                   ate
                                   breakfast
                                   ..
                                   went
                                   back
                                   to
                                   sleep
                                   ..
                                   up
                                   again
                                   ..
                                   bout
                                   to
                                   get
                                   right
                                   for
                                   da
                                   beach
                                   blast
                                   ..
                                   **leeggoo**
                                   !!- perkins
                                     n
                                     chandler
                                     goin
                                     at
                                     it
                                     ..
                                     ha
                                     ..
                                     **leeggoo**
                                     mavs- smh
                                       twitter
                                       is
                                       live
                                       dis
                                       morning
                                       **leeggoo**- @bellalalalalala
                                         @chickeninpocket
                                         hell
                                         yeaah
                                         **leeggoo**

## llegar

1. @sick\_doll
   nada
   como
   **llegar**
   empapado
   y
   que
   pantera
   me
   llore
   para
   que
   lo
   deje
   salir
   .
   lo
   que
   no
   sabe
   es
   que
   soy
   muy
   duro
   de
   convencer
   !!- despues
     de
     11
     hrs
     de
     carretera
     +
     7
     hrs
     de
     puente
     para
     cruzar
     **llegar**
     a
     casa
     no
     tiene
     precio
     ..
     pero
     ha
     valido
     la
     pena
     la
     he
     pasado
     genial
     !- con
       la
       esperanza
       de
       **llegar**
       a
       tiempo
       para
       ver
       el
       partido- ya
         quiero
         **llegar**
         :/
         a
         mi
         casa
         !!
         me
         siento
         super
         cansado
         !!- **llegar**
           y
           carnita
           asada- entrando
             a
             texas
             y
             ya
             se
             rajaron
             todos
             .
             ni
             me
             dejaron
             **llegar**
             a
             dallas
             .
             buen
             inicio
             de
             semestre
             a
             todos
             mis
             amiguitos
             .- @sindylazo
               hola
               sindy
               ,
               como
               hago
               para
               hacerte
               **llegar**
               un
               guion
               ?
               hay
               un
               papel
               q
               me
               gustaria
               interpretaras
               ..- fucking
                 luu
                 acaba
                 de
                 **llegar**
                 -.-- tonteando
                   en
                   twitter
                   antes
                   de
                   **llegar**
                   al
                   doc
                   .- @juanriveramusic
                     sigan
                     con
                     sus
                     mamadas
                     y
                     tu
                     y
                     tu
                     hermana
                     se
                     les
                     va
                     a
                     **llegar**
                     !!
                     se
                     ponen
                     d
                     pesaditos
                     en
                     mexico
                     aya
                     se
                     van
                     a
                     kedar
                     !- camino
                       a
                       austin
                       ,
                       tx
                       ..
                       ya
                       quiero
                       **llegar**
                       a
                       casita
                       :(- @hadacon
                         desafortunadamente
                         es
                         lunes
                         ..
                         pero
                         para
                         **llegar**
                         a
                         viernes
                         ,
                         lunes
                         es
                         necesario
                         ..- @doritosr
                           jajajajajajaja
                           y
                           que
                           flojera
                           de
                           verdad
                           eso
                           ,
                           te
                           aseguro
                           que
                           mucha
                           gente
                           va
                           a
                           **llegar**
                           a
                           pedir
                           su
                           dinero
                           y
                           hasta
                           unas
                           papas
                           gratis
                           jaj- @henrysantos
                             @maxi\_iglesias
                             como
                             estas
                             henry
                             ..
                             los
                             kiero
                             ver
                             **llegar**
                             a
                             la
                             final
                             ..
                             de
                             todos
                             tu
                             eres
                             el
                             rival
                             a
                             vencer
                             ,
                             #ganarashenry
                             ,
                             suguro- @monikin2
                               a
                               po
                               tu
                               no
                               va
                               a
                               **llegar**
                               a
                               la
                               playa
                               !!
                               k
                               mal
                               loka
                               !- y
                                 al
                                 **llegar**
                                 a
                                 hacer
                                 tu
                                 check-in
                                 te
                                 sale
                                 la
                                 maravillosa
                                 sorpresa
                                 que
                                 para
                                 chequear
                                 un
                                 segunda
                                 maleta
                                 debes
                                 pagar
                                 $70
                                 ..
                                 genial- tengo
                                   una
                                   duda
                                   ,
                                   cuando
                                   el
                                   blackberry
                                   esta
                                   en
                                   3g
                                   pueden
                                   **llegar**
                                   los
                                   pin
                                   ?
                                   @bberryblog- @angetkieremucho
                                     pasado
                                     maniana
                                     !!
                                     ya
                                     quiero
                                     **llegar**
                                     !- @escandalotv
                                       kee
                                       ??
                                       cristian
                                       chavez
                                       se
                                       casa
                                       otra
                                       vez
                                       adonde
                                       vamos
                                       a
                                       **llegar**
                                       con
                                       estas
                                       lokas
                                       ,
                                       estan
                                       lokas
                                       lokas- ser
                                         la
                                         ultimas
                                         en
                                         **llegar**
                                         y
                                         **llegar**
                                         con
                                         un
                                         buen
                                         de
                                         bolsas
                                         y
                                         decir
                                         me
                                         quedare
                                         en
                                         la
                                         ruina
                                         #noporquelahagomimejoramiga
                                         @aylinsandoval

## niiccee

1. @jhlotto
   yea
   ..
   girls+spanx
   =
   **niiccee**- **niiccee**- @madeiinjamaica
       lol
       **niiccee**- drinkn
         0n
         this
         rum
         n
         barcardi
         !!
         feel'n
         uhm
         quite
         **niiccee**
         !- feelin
           jealous
           ..
           hahart
           @dianiajay
           :
           @antb00gi3
           feelin
           **niiccee**- big
             bro
             @louardo
             welcome
             me
             out
             of
             work
             with
             a
             nice
             40
             oz
             sol
             ..
             **niiccee**
             ..- was
               on
               your
               website
               last
               night
               ..
               **niiccee**
               !
               @itsbbod
               @monalisa21
               @sexxylexxy1
               @missmoemoney- @dnellztristate
                 church
                 ,
                 tri
                 state
                 is
                 realluy
                 bout
                 to
                 take
                 ova
                 ,
                 ohh
                 yeaa
                 **niiccee**- @loonee16
                   that's
                   cool
                   gonna
                   be
                   **niiccee**
                   out
                   today
                   and
                   tomorrow- i'm
                     loving
                     all
                     these
                     documentaries
                     i've
                     watched
                     ..
                     first
                     about
                     miami
                     football
                     and
                     then
                     n.w.a
                     !!
                     **niiccee**
                     !!- nutcraccah
                       got
                       me
                       feelin
                       **niiccee**- got
                         a
                         **niiccee**
                         lil
                         water
                         hole
                         on
                         mondays
                         and
                         i'm
                         keeping
                         it
                         close
                         to
                         the
                         chest
                         #fo'realthough- its
                           **niiccee**
                           outside
                           ..
                           2
                           bad
                           i
                           have
                           2
                           work
                           (
                           smfh
                           )- young
                             money
                             cd
                             is
                             **niiccee**
                             ..
                             but
                             it
                             would
                             be
                             harder
                             if
                             everybody
                             had
                             longer
                             verses
                             ..
                             #imjustsayin- mangoes
                               was
                               **niiccee**- **niiccee**- @hanksb
                                   @turbalance
                                   yea
                                   @brinathemodel
                                   is
                                   (
                                   fabolous
                                   voice
                                   )
                                   **niiccee**
                                   !
                                   #instantfollow- @dippedinbadd
                                     mm
                                     ..
                                     ii
                                     thought
                                     yu
                                     were
                                     jokiin
                                     ..
                                     yu
                                     got
                                     a
                                     **niiccee**
                                     flow
                                     !- @jeff\_ischill
                                       **niiccee**
                                       ,
                                       good
                                       lkn
                                       .- @stackbundles
                                         **niiccee**

## oommgg

1. **oommgg**
   ..
   i'm
   out
   of
   water
   !
   anyone
   who
   knows
   me
   ,
   knows
   i
   always
   have
   #liq
   and
   #water
   in
   my
   house
   at
   all
   times
   !- **oommgg**
     why
     is
     ur
     back
     on
     the
     flloorr
     whhoorree
     !..
     #ugh- you
       are
       the
       slowest
       cashier
       i've
       ever
       seen
       **oommgg**- **oommgg**
         i'm
         in
         mobile
         and
         they
         got
         juve's
         bounce
         back
         on
         the
         radio
         .
         i'm
         so
         #louisianabound
         i-10
         baby- **oommgg**
           if
           your
           texting
           me
           all
           day
           and
           i
           don't
           answer
           ,
           shouldn't
           that
           tell
           you
           something
           ?
           helloo
           get
           a
           clue- **oommgg**
             its
             your
             birthday
             ,
             happy
             birthday
             damu
             ..
             let's
             party- **oommgg**
               the
               proposal
               is
               too
               funny
               !
               lol
               this
               part
               with
               margret
               and
               the
               grandmother
               in
               the
               woods- @ms\_taliaferro08
                 **oommgg**
                 !!
                 i
                 know
                 !!
                 i've
                 seen
                 everybody
                 this
                 summer
                 but
                 u
                 and
                 effin
                 @hurlz\_jr
                 #pissed- **oommgg**
                   !!
                   im
                   like
                   a
                   fiend
                   im
                   going
                   thru
                   some
                   serious
                   withdrawals
                   here
                   !!
                   like
                   seriously- @djvelz
                     **oommgg**
                     ur
                     nuts- **oommgg**
                       if
                       1
                       more
                       person
                       tells
                       me
                       me
                       and
                       favorite
                       need
                       to
                       hurry
                       up
                       and
                       get
                       together
                       ima
                       go
                       crazy
                       lol
                       ..
                       i
                       like
                       hearing
                       it
                       though
                       :)- @i\_perfectedsex
                         lol
                         **oommgg**
                         that's
                         crazy
                         i'm
                         too
                         nice
                         to
                         be
                         a
                         killer- @no\_impostors
                           **oommgg**
                           tatii
                           u
                           go
                           to
                           sleep- **oommgg**
                             shut
                             that
                             shit
                             up- lmaoo
                               yo
                               ass
                               b
                               tweakin
                               grt
                               @sunshyneblvd
                               :
                               **oommgg**
                               he
                               has
                               two
                               thumbs
                               omg- @dontsleep\_on10c
                                 @kiasojazzy
                                 @uptown140
                                 @myolekutetass
                                 **oommgg**
                                 get
                                 the
                                 fuck
                                 outta
                                 here
                                 .!
                                 where
                                 the
                                 fuck
                                 was
                                 that
                                 .!?- **oommgg**
                                   !!
                                   that
                                   was
                                   a
                                   close
                                   one
                                   by
                                   the
                                   u.s.
                                   !!- @sponser\_girl
                                     **oommgg**
                                     why
                                     they
                                     firing
                                     everybody
                                     !- @callme\_samii
                                       **oommgg**
                                       i
                                       wanna
                                       see
                                       him
                                       eat
                                       a
                                       hot
                                       chip
                                       .- @
                                         \_missbarbie\_
                                         @kwamainef
                                         **oommgg**
                                         are
                                         those
                                         her
                                         titties
                                         ??
                                         wtff
                                         !
                                         put
                                         that
                                         shit
                                         up
                                         !!
                                         -->
                                         @mzjuicywetwet1

## otros

1. soy
   yo
   o
   mi
   clima
   no
   enfria
   tanto
   como
   **otros**
   dias- @massieljavier
     no
     en
     vivo
     y
     directo
     desde
     la
     oficina
     y
     digo
     k
     parece
     wealth
     fare
     x
     k
     solo
     hay
     boricuas
     negros
     entre
     **otros**
     latinos- pendejos
       de
       **otros**
       bancos
       que
       creen
       que
       trabajas
       para
       ellos
       (
       y
       en
       dia
       feriado
       )
       jajaja- comuna
         :
         arte
         de
         matarse
         los
         unos
         a
         los
         **otros**
         ,
         ya
         sea
         por
         una
         arepa
         ,
         bloque
         etc- @badmilfx3
           ahh
           bueno
           los
           cuernos
           son
           para
           las
           vacas
           y
           **otros**
           animales
           i
           think
           you
           .
           better
           dem
           that- quiero
             aclarar
             que
             no
             todos
             los
             mexicanos
             son
             maltratados
             en
             az
             .
             algunos
             mexicanos
             son
             maltratados
             en
             **otros**
             estados- @summersnothot
               noo
               ..
               :'(
               !!
               casi
               no
               viene
               a
               florida
               ..
               la
               verdad
               creo
               que
               se
               la
               pasa
               mas
               por
               mexico
               .
               y
               **otros**
               estados
               mas
               de
               usa
               pero
               no
               aqui- @maru\_navaira
                 ayte
                 van
                 los
                 **otros**
                 names
                 :
                 ashley
                 ciclalytl
                 ,
                 nahuytl
                 shelsea
                 ,
                 joshelym
                 athzirytl
                 .
                 checa
                 todos
                 con
                 sh
                 y
                 ytl
                 #kiovole
                 ??
                 paike
                 vdd
                 ?- @carlenny
                   yo
                   no
                   me
                   desesperoo
                   si
                   **otros**
                   pueden
                   yo
                   tambien- @sharoncuevas
                     @gilbertkeyz
                     entre
                     ustedes
                     y
                     sara
                     tambien
                     me
                     tienen
                     el
                     twitter
                     cargado
                     !
                     no
                     me
                     dejan
                     ver
                     los
                     twits
                     de
                     **otros**
                     de
                     pol
                     dioo
                     lol
                     !- lo
                       que
                       es
                       poco
                       para
                       algunos
                       ,
                       es
                       mucho
                       para
                       **otros**
                       .- en
                         la
                         vida
                         estoy
                         acostumbrado
                         a
                         competir
                         ,
                         desde
                         que
                         le
                         gane
                         la
                         carrera
                         a
                         los
                         **otros**
                         espermatozoides- con
                           mi
                           compa
                           julyy
                           bebiendoo
                           o
                           y
                           los
                           **otros**
                           primos
                           ,
                           mi
                           familia
                           ee
                           loka
                           sheesh
                           smh
                           con
                           razon
                           yo
                           soi
                           media
                           atota
                           tambien- @mariagabrielamm
                             hay
                             verga
                             jajaja
                             que
                             vos
                             tengais
                             **otros**
                             gustos
                             es
                             otra
                             verga
                             jaja
                             a
                             vos
                             te
                             gustan
                             asi
                             tipo
                             no
                             se
                             mejor
                             me
                             callo
                             la
                             jeta
                             jaja- @anngelazul
                               jajajaj
                               no
                               see
                               pero
                               yo
                               tengo
                               cosas
                               k
                               m
                               informan
                               tambien
                               jajaja
                               asi
                               no
                               espero
                               aver
                               ke
                               dicen
                               **otros**
                               jajaj- @crackberrista
                                 tambien
                                 tengo
                                 una
                                 lista
                                 de
                                 los
                                 dos
                                 **otros**
                                 que
                                 me
                                 salieron
                                 y
                                 estoy
                                 segura
                                 que
                                 es
                                 una
                                 aplicacion
                                 ,
                                 no
                                 se
                                 cual
                                 .
                                 (
                                 202
                                 )
                                 y
                                 (
                                 66
                                 )
                                 .
                                 gra
                                 !- en
                                   el
                                   cumple
                                   de
                                   @el\_chocorrol
                                   con
                                   el
                                   novio
                                   ,
                                   @llevelellevele
                                   @hat\_keeper
                                   @diadeoctubre
                                   entre
                                   **otros**
                                   (
                                   sin
                                   twitter
                                   )- @cuqui03
                                     xuzo
                                     men
                                     k
                                     desgracias
                                     pa
                                     unos
                                     felicidad
                                     pa
                                     **otros**
                                     ese
                                     man
                                     viaje
                                     de
                                     lujo
                                     y
                                     sin
                                     pagar
                                     el
                                     boleto
                                     y
                                     yo
                                     loca
                                     x
                                     ir
                                     y
                                     tengo
                                     k
                                     ahorra- @andreiitax3
                                       lololololol
                                       yo
                                       trabajo
                                       eske
                                       mi
                                       horario
                                       es
                                       mas
                                       corto
                                       q
                                       **otros**
                                       lol- no
                                         acostumbro
                                         a
                                         darle
                                         un
                                         trago
                                         a
                                         la
                                         botella
                                         que
                                         **otros**
                                         le
                                         toman

## pueden

1. @mahitafunes
   a
   mi
   no
   me
   bloqueo
   ,
   pero
   a
   las
   demas
   si
   porque
   no
   **pueden**
   ver
   sus
   tweets
   y
   no
   **pueden**
   seguirlo
   y
   no
   le
   llega
   lo
   que
   le
   escriben- no
     te
     meta
     con
     nosotra
     palomaa
     no
     t
     emeta
     con
     la
     jente
     k
     te
     **pueden**
     da
     plomoo
     !!
     lmll
     ..
     mad
     hypee
     =]- @alexsensation
       no
       critico
       el
       precio
       compa
       ,
       soy
       dominicano
       me
       gusta
       omega
       pero
       no
       se
       **pueden**
       comparar
       !
       recuerda
       en
       el
       ambiente
       k
       tamos
       !- estoy
         en
         nota
         curandome
         con
         rapero
         fictisios
         jajajaja
         ,
         **pueden**
         bajar
         la
         musica
         nueva
         de
         aro
         sanchez
         por
         itunes
         muy
         pronto
         !!- @alfonsoleon10
           alfonzo
           que
           colores
           se
           le
           **pueden**
           poner
           a
           un
           local
           de
           venta
           de
           equipos
           telefonicos
           y
           tecnologia
           para
           activar
           las
           ventas- con
             un
             dispensador
             se
             ahorran
             el
             pago
             de
             15
             empleados
             ,
             +
             local
             ,
             servicios
             .
             **pueden**
             rentar
             +
             barato
             y
             compiten
             con
             la
             pirateria
             @tibiguerra- @biigredlmp
               @mzgemzlmp
               ella
               e
               una
               mula
               .
               las
               mulas
               no
               **pueden**
               parir
               .- @mr\_marin
                 @diegopulecio
                 dejen
                 la
                 envidia
                 .
                 voy
                 por
                 el
                 millon
                 de
                 followers
                 .
                 no
                 **pueden**
                 ver
                 a
                 un
                 pobre
                 triunfar
                 !- @cynthia\_uriastv
                   no
                   te
                   preocupes
                   seguro
                   estoy
                   de
                   que
                   vendra
                   algo
                   importante
                   en
                   el
                   futuro
                   ,
                   tienes
                   muchas
                   virtudes
                   ,
                   y
                   no
                   se
                   **pueden**
                   desaprovechar- @infobae
                     el
                     mundo
                     gira
                     hacia
                     un
                     lado
                     y
                     nosotros
                     hacia
                     otro
                     .
                     vivimos
                     en
                     el
                     mundo
                     del
                     reves
                     .
                     se
                     van
                     x
                     q
                     no
                     **pueden**
                     generar
                     nada
                     con
                     argentina- @sgalizzi14
                       @luzmarvis
                       @luzmarvis
                       @pjimenez\_lopez
                       se
                       **pueden**
                       cuidar
                       ..!!- **pueden**
                         creer
                         que
                         hasta
                         ahora
                         voy
                         a
                         ver
                         el
                         partido
                         solo
                         he
                         visto
                         los
                         updates
                         de
                         ustedes
                         ,
                         me
                         voy
                         a
                         poner
                         la
                         verde
                         a
                         ver
                         si
                         ocurre
                         un
                         milagro- @reyes\_mr
                           @soniasev
                           pues
                           si
                           pero
                           no
                           **pueden**
                           estar
                           seperados
                           tantos
                           capitulso
                           y
                           de
                           una
                           manera
                           tan
                           incohernte
                           un
                           dia
                           renata
                           esta
                           con
                           el
                           y- @orlansoco
                             que
                             bueno
                             !.
                             asi
                             es
                             !
                             unidos
                             no
                             **pueden**
                             con
                             nosotors
                             !- come
                               on
                               !!
                               no
                               tengo
                               palabras
                               ,
                               alomenos
                               mexico
                               califico
                               .
                               pinche
                               uruguay
                               y
                               argentina
                               se
                               **pueden**
                               ir
                               al
                               infuerno
                               !- el
                                 amor
                                 ,
                                 la
                                 paz
                                 y
                                 la
                                 alegria
                                 no
                                 **pueden**
                                 florecer
                                 hasta
                                 q
                                 ud
                                 se
                                 haya
                                 liberado
                                 del
                                 dominio
                                 d
                                 la
                                 mente
                                 ..- no
                                   entiendo
                                   como
                                   **pueden**
                                   pasar
                                   por
                                   direct
                                   v-v
                                   zla
                                   el
                                   miss
                                   vzla
                                   en
                                   hd
                                   y
                                   no
                                   pasan
                                   los
                                   juegos
                                   de
                                   la
                                   vino
                                   tinto
                                   en
                                   hd
                                   !
                                   toda
                                   la
                                   se
                                   #al
                                   debe
                                   ser
                                   hd
                                   !!- @luischataing
                                     noo
                                     **pueden**
                                     van
                                     a
                                     gastar
                                     muucha
                                     agua
                                     y
                                     eso
                                     esta
                                     ahorita
                                     prohibido- @artistasfcmaria
                                       el
                                       telefono
                                       esta
                                       muerto
                                       se
                                       me
                                       quedo
                                       el
                                       cargador
                                       en
                                       la
                                       oficina
                                       me
                                       **pueden**
                                       llamar
                                       al
                                       tel
                                       de
                                       google
                                       832
                                       380
                                       5386- no
                                         te
                                         meta
                                         con
                                         la
                                         giente
                                         que
                                         te
                                         **pueden**
                                         da
                                         plomo

## quieren

1. la
   gente
   que
   no
   tiene
   na
   son
   lo
   que
   ma
   **quieren**
   llama
   la
   atencion
   #shittttt- despues
     que
     nombro
     las
     tiendas
     que
     amo
     ,
     entonces
     van
     y
     las
     **quieren**
     asalar
     -.-
     ash
     !- @oswaldoalvarez
       pero
       **quieren**
       @chavezcandanga
       :
       apoyo
       a
       paraguay
       en
       su
       batalla
       contra
       la
       violencia
       criminal- @migueloz
         se
         **quieren**
         hacer
         los
         experimentales
         y
         alternativos
         y
         la
         neta
         no
         les
         va
         ,
         nomas
         la
         acaban
         cagando- @mrs\_hardita
           podemos
           comensar
           a
           conocernos
           tenemos
           todo
           el
           finde
           senana
           para
           eso
           y
           dire
           k
           **quieren**
           decir
           las
           xx- **quieren**
             q
             me
             muera
             pa
             poner
             mi
             cara
             en
             un
             poloche
             q
             diga
             por
             siempre
             lo
             recordare
             .
             si
             y
             se
             **quieren**
             vever
             mi
             cafe
             .- hahaha
               **quieren**
               vr
               una
               paliiza
               ?
               vean
               el
               juego
               ..
               a
               australiia
               le
               estan
               ense\*ando
               a
               bailar- @satelliteeheart
                 .
                 realmente
                 **quieren**
                 seguir
                 juntos
                 o
                 no
                 ?
                 que
                 los
                 obliga
                 ?- @louiseflorcita
                   todas
                   **quieren**
                   el
                   corazon
                   del
                   @bisarro
                   es
                   el
                   sexsimbol
                   de
                   twitter- "
                     voy
                     a
                     ir
                     a
                     pollo
                     rico
                     .
                     **quieren**
                     ?
                     esta
                     rico
                     el
                     pollo
                     ..
                     "
                     #noshitsherlock- el
                       mundo
                       esta
                       lleno
                       de
                       muchos
                       misterios
                       y
                       verdades
                       que
                       no
                       nos
                       **quieren**
                       decir
                       ..- @cougar\_pr
                         @tony\_banana
                         mi
                         consejo
                         es
                         que
                         hable
                         con
                         tasadores
                         locales
                         (
                         varios
                         )
                         ,
                         no
                         corredores
                         ,
                         ellos
                         lo
                         que
                         **quieren**
                         es
                         venderte
                         .- gente
                           que
                           solo
                           **quieren**
                           que
                           le
                           digan
                           lo
                           que
                           **quieren**
                           escuchar
                           y
                           asi
                           !- cuando
                             ven
                             que
                             sigues
                             adelante
                             sin
                             ellos
                             ,
                             es
                             cuando
                             te
                             **quieren**
                             de
                             regreso
                             .- que
                               ladilla
                               con
                               la
                               gente
                               que
                               no
                               deja
                               que
                               los
                               otros
                               fumen
                               -.-
                               es
                               su
                               vida
                               ,
                               son
                               sus
                               pulmones
                               si
                               se
                               **quieren**
                               morir
                               dejenlos
                               es
                               su
                               decision
                               .- si
                                 **quieren**
                                 reirse
                                 un
                                 ratito
                                 de
                                 nuestra
                                 tragedia
                                 nacional
                                 ,
                                 les
                                 recomiendo
                                 una
                                 visitada
                                 a
                                 #preguntasparafelipe- @majoruano
                                   @maflopz
                                   llamenme
                                   si
                                   **quieren**
                                   :)
                                   aurita
                                   solo
                                   me
                                   avisa
                                   antes- estas
                                     mujeres
                                     me
                                     **quieren**
                                     volver
                                     loco
                                     ..
                                     pero
                                     que
                                     hare
                                     lol
                                     .- @periodismo\_es
                                       pero
                                       yo
                                       no
                                       soy
                                       famosa
                                       jaa
                                       pa
                                       q
                                       **quieren**
                                       una
                                       entrevista
                                       mia
                                       ??- lo
                                         mas
                                         cabron
                                         es
                                         q
                                         c
                                         **quieren**
                                         hacer
                                         las
                                         santa
                                         siempre

## recuerdo

1. @evesbk
   lol
   sabes
   me
   **recuerdo**
   mi
   madre
   telling
   me
   ,
   not
   to
   get
   a
   girl
   prego
   b4
   i
   even
   knew
   bout
   birds
   &
   bees
   !!
   lol
   #madres\_sonhermosas
   !!- **recuerdo**
     la
     primera
     vez
     que
     hoy
     gee
     !
     hehehhe
     no
     sabia
     lo
     que
     decia
     ..
     pero
     yo
     decia
     :
     ndhsukdkrkda
     gee
     gee
     gee
     gee
     baby
     baby
     :d- **recuerdo**
       que
       la
       arena
       no
       era
       tan
       oscura
       y
       tu
       voluntad
       no
       era
       tan
       dura
       ..- ahora
         es
         que
         **recuerdo**
         que
         yo
         no
         eh
         comido
         en
         todo
         el
         dia
         !- tu
           **recuerdo**
           son
           mi
           dios- @maar27
             gracias
             mi
             lokita
             hermosa
             .
             aunque
             no
             hablamos
             mucho
             siempre
             te
             **recuerdo**
             .
             un
             besote
             para
             ti
             .- @evesosa
               hey
               ahora
               q
               **recuerdo**
               nosotros
               tenemos
               unos
               dm's
               pendientes
               jajajajaja
               !!
               no
               te
               me
               hagas
               la
               mariada
               !!
               :p- @shroudripper
                 no
                 **recuerdo**
                 estuvo
                 muy
                 cabrona
                 la
                 cruda- @malignoreshu
                   yo
                   la
                   vi
                   en
                   el
                   2009
                   y
                   aun
                   **recuerdo**
                   el
                   dia
                   como
                   si
                   fuere
                   hace
                   una
                   semana- son
                     queridas
                     las
                     heridas
                     ..
                     quizas
                     porque
                     marcan
                     tu
                     **recuerdo**
                     .- @lustown
                       oh
                       si
                       ,
                       yo
                       **recuerdo**
                       haber
                       pasado
                       por
                       el
                       ipn
                       .
                       such
                       is
                       life
                       !- lamento
                         contrariarlo
                         pero
                         yo
                         ..
                         no
                         lo
                         **recuerdo**
                         ..,,- :
                           no
                           te
                           arrepientas
                           de
                           nada
                           ..
                           si
                           es
                           bueno
                           es
                           un
                           **recuerdo**
                           ..
                           si
                           es
                           malo
                           es
                           una
                           experiencia
                           .- ..
                             y
                             esto
                             que
                             no
                             era
                             amor
                             ,
                             lo
                             que
                             hoy
                             niegas
                             .
                             lo
                             que
                             dices
                             que
                             nunca
                             paso
                             ,
                             es
                             el
                             mas
                             dulce
                             **recuerdo**
                             de
                             mi
                             vida
                             ..- no
                               dejo
                               de
                               asombrarme
                               de
                               todo
                               lo
                               que
                               dios
                               da
                               ..
                               lo
                               malo
                               es
                               cuando
                               no
                               lo
                               **recuerdo**
                               ..- un
                                 **recuerdo**
                                 siempre
                                 te
                                 persigue
                                 ,
                                 con
                                 el
                                 fin
                                 de
                                 ser
                                 escrito
                                 con
                                 tinta
                                 permanente
                                 ,
                                 pues
                                 no
                                 puede
                                 ser
                                 borrado
                                 .- @liqs
                                   mh
                                   ..
                                   porque
                                   no
                                   **recuerdo**
                                   tal
                                   ?- "
                                     y
                                     hoy
                                     guardo
                                     tu
                                     **recuerdo**
                                     ,
                                     como
                                     el
                                     mejor
                                     secreto
                                     ,
                                     que
                                     dulce
                                     fue
                                     tenerte
                                     dentro
                                     .
                                     hay
                                     un
                                     trozo
                                     de
                                     luz
                                     ,
                                     en
                                     esta
                                     oscuridad
                                     para
                                     prestarme
                                     clama
                                     "- **recuerdo**
                                       cuando
                                       history
                                       channel
                                       era
                                       seriesisimo
                                       y
                                       el
                                       comercial
                                       de
                                       the
                                       dark
                                       ages
                                       era
                                       con
                                       suffocation
                                       .
                                       ahora
                                       es
                                       basura
                                       .
                                       >:(- @alexiimena
                                         @sandritangel
                                         @zully\_fod
                                         no
                                         **recuerdo**
                                         bien
                                         ese
                                         tweet
                                         ,
                                         xd
                                         .

## seguro

1. @claudiaruiz
   ve
   ,
   ayer
   tuitie
   un
   par
   de
   articulos
   del
   asunto
   indigena
   .
   estoy
   **seguro**
   q
   te
   interesaran
   y
   gustaran
   .
   buscalos
   en
   mis
   twts
   .- @luisfgomezb
     gracias
     amigo
     luis
     que
     tengas
     un
     feliz
     y
     muy
     **seguro**
     viernes- @elcesar\_kn
       gracias
       mi
       vida
       pues
       mi
       dia
       ekiz
       que
       tal
       el
       tuyo
       ?
       si
       **seguro**
       si
       voy
       con
       mishel- @edileyojeda
         .
         mi
         hermana
         acuerdese
         que
         barinas
         es
         de
         los
         chavez
         por
         esa
         vaina
         es
         !!
         y
         **seguro**
         que
         tu
         luis
         fonsi
         debe
         cobrar
         dos
         lochas
         !- minutos
           antes
           de
           bajarselo
           de
           **seguro**
           alguno
           se
           tiro
           el
           :
           where
           have
           you
           bin
           ?
           #obama1
           -osama0- pronto
             llegara
             el
             dia
             de
             mi
             suerte
             ..
             se
             que
             ante
             de
             mi
             muerte
             **seguro**
             que
             mi
             suerte
             cambiara
             ..- @priscimorenaj96
               umm
               mira
               nomas
               pos
               hay
               aber
               cuando
               amiga
               pos
               yo
               aca
               de
               **seguro**
               me
               largo
               a
               un
               lugar
               ?
               jejej
               como
               siempre
               eda
               ?- pero
                 de
                 **seguro**
                 @lipaoc
                 tiene
                 un
                 corazon
                 muy
                 grande
                 que
                 comparta
                 jeje
                 #amanecerw- @da\_pineda
                   que
                   es
                   muy
                   **seguro**
                   q
                   no
                   vaya
                   @adriruizma
                   quiere
                   ir
                   reserva
                   colocha
                   este
                   s
                   l
                   numero
                   6026875- @benjamin\_reyna
                     hahaha
                     y
                     k
                     tal
                     la
                     fiesta
                     d
                     **seguro**
                     andas
                     super
                     crudo
                     .- @ginasalazarm
                       creo
                       que
                       atleta
                       que
                       se
                       niegue
                       ayuda
                       que
                       le
                       quitan
                       **seguro**
                       y
                       la
                       mayoria
                       vive
                       afuera
                       de
                       vzla
                       la
                       unica
                       forma
                       de
                       llegar
                       alto
                       nivel- @compositorcarl
                         **seguro**
                         le
                         hecho
                         un
                         vistazo
                         y
                         te
                         dejo
                         mi
                         critica
                         ,
                         me
                         gusta
                         mucho
                         el
                         trabajo
                         de
                         joan
                         ,
                         es
                         un
                         artista
                         muy
                         completo
                         .- @maderagomez
                           es
                           un
                           cantante
                           grupero
                           y
                           pues
                           **seguro**
                           tenia
                           algun
                           con
                           la
                           mafia
                           .- @fugamx
                             te
                             mande
                             dos
                             o
                             tres
                             tweets
                             jodiendote
                             con
                             tu
                             robo
                             de
                             imagen
                             ,
                             dije
                             **seguro**
                             ya
                             se
                             enojoo
                             por
                             q
                             lo
                             jodi
                             y
                             la
                             puse
                             tambien
                             x
                             joderlo- @elpana
                               sii
                               es
                               que
                               me
                               da
                               mucha
                               pereza
                               ir
                               a
                               una
                               academia
                               ahi
                               equis
                               y
                               **seguro**
                               lo
                               ponen
                               a
                               uno
                               a
                               tocar
                               tonteras
                               ..
                               prefiero
                               pagarle
                               a
                               ud
                               :p- @niko\_\_realg
                                 saludos
                                 **seguro**
                                 ya
                                 te
                                 tire
                                 el
                                 follow- @heeldaary
                                   eso
                                   dalo
                                   por
                                   **seguro**
                                   sipota
                                   ;
                                   )- @chinobrown
                                     oorraahh
                                     !
                                     manana
                                     de
                                     **seguro**
                                     homey
                                     vamos
                                     al
                                     tot- @henkelgarcia
                                       @suhelistejero
                                       exactamente
                                       ,
                                       pero
                                       **seguro**
                                       ya
                                       estamos
                                       perdiendo
                                       followers
                                       por
                                       criticarlo
                                       =p- @beyafaby
                                         si
                                         fuiste
                                         asi
                                         ,
                                         de
                                         **seguro**
                                         pasaste

## tengan

1. good
   night
   all
   !
   q
   **tengan**
   buenas
   noches
   :)- @ruthpolanco
     @nelfinova
     @ifanini
     @amandysha
     @pavelonsky
     @bregna
     buenos
     dias
     a
     todos
     .
     que
     **tengan**
     un
     dia
     productivo
     .- buenas
       noches
       gente
       ,
       que
       **tengan**
       un
       excelente
       inicio
       de
       semana
       (:- tgif
         <
         que
         **tengan**
         todos
         un
         buen
         fin
         de
         semana
         con
         muchas
         energia
         positiva
         ..- un
           super
           fin
           de
           esparcimiento
           y
           crecimiento
           personal
           .
           gracias
           tats
           y
           david
           !
           aunq
           no
           **tengan**
           twitter- buenos
             dias
             **tengan**
             todos
             por
             aqui
             ..
             bello
             lunes
             y
             hay
             q
             aprovecharlo
             .
             saludines
             .- aa
               trabajar
               se
               dijo
               ..
               q
               **tengan**
               un
               buen
               dia- saludoos
                 =d
                 quelocaruan
                 gente
                 !!
                 besos
                 pa
                 '
                 to
                 el
                 mundo
                 **tengan**
                 un
                 lindo
                 diaa
                 !!- @izarradeverdad
                   mandaste
                   a
                   lanzar
                   cohetes
                   a
                   las
                   8
                   pm
                   rizarra
                   ?
                   para
                   tapar
                   ruido
                   de
                   cacerolas
                   ?
                   espero
                   **tengan**
                   bastantes
                   ,
                   como
                   los
                   traen
                   d
                   china- q
                     **tengan**
                     un
                     buenisimo
                     finde
                     !
                     :)- buen
                       dia
                       tl
                       !!
                       que
                       **tengan**
                       tod
                       @s
                       un
                       excelente
                       martes
                       !!
                       #losdelrojo
                       #losdeltinto- por
                         favor
                         ahora
                         todos
                         los
                         rest
                         .
                         de
                         valencia
                         tienen
                         sushi
                         **tengan**
                         un
                         poquito
                         mas
                         de
                         creatividad
                         gracias- @nadamasana
                           no
                           porque
                           **tengan**
                           un
                           huesito
                           "
                           en
                           un
                           partido
                           .
                           hay
                           hermana
                           no
                           eres
                           normal
                           .- @cartel\_jr
                             buenos
                             dias
                             a
                             todos
                             k
                             **tengan**
                             un
                             divertido
                             dia- buenas
                               las
                               **tengan**
                               banda
                               !!
                               preguntal
                               pendeja
                               .!!
                               trabajas
                               .
                               >>
                               like
                               estudias
                               >>
                               comenta
                               las
                               dos
                               cosas))like
                               estas
                               en
                               la
                               hueva
                               >>
                               comenta- si
                                 el
                                 camino
                                 esta
                                 lleno
                                 de
                                 obstaculos
                                 quiere
                                 decir
                                 q
                                 vaz
                                 en
                                 direccion
                                 correcta
                                 !!
                                 buena
                                 vibra
                                 amigos
                                 q
                                 **tengan**
                                 un
                                 dia
                                 bello
                                 para
                                 la
                                 historia
                                 !!- sonrie
                                   siempre
                                   para
                                   q
                                   los
                                   q
                                   t
                                   odian
                                   no
                                   **tengan**
                                   l
                                   placer
                                   d
                                   verte
                                   triste
                                   ,
                                   y
                                   los
                                   q
                                   t
                                   queremos
                                   **tengan**
                                   la
                                   satisfaccion
                                   d
                                   verte
                                   reir
                                   .
                                   buen
                                   dia
                                   !!- @itzaena
                                     @gusilvia
                                     que
                                     **tengan**
                                     un
                                     lindo
                                     fin
                                     !!
                                     no
                                     perdamos
                                     la
                                     fe
                                     de
                                     que
                                     pronto
                                     veremos
                                     el
                                     gran
                                     despegue
                                     !!
                                     de
                                     pie
                                     hasta
                                     la
                                     victoria
                                     !!- en
                                       el
                                       trabajoo
                                       !!
                                       que
                                       **tengan**
                                       todos
                                       muy
                                       buenos
                                       dias
                                       !!- buenos
                                         dias
                                         feliz
                                         dia
                                         de
                                         la
                                         inmcaulada
                                         concepcion
                                         .
                                         espero
                                         que
                                         **tengan**
                                         un
                                         bello
                                         martes
                                         ,
                                         ya
                                         regreso
                                         ,
                                         para
                                         que
                                         inter
                                         actuemos
                                         .

## tengas

1. @lacozinela\_j51
   hello
   k
   **tengas**
   un
   maravilloso
   dia
   un
   dia
   lleno
   de
   bendiciones
   mi
   cozi-bella
   :-)
   god
   bless
   you
   always
   corazon
   ..
   muah
   muah
   muah- esto
     es
     a
     fuerza
     de
     cantazos
     ..
     no
     creas
     que
     la
     vida
     dura
     ,
     no
     le
     **tengas**
     miedo
     a
     los
     fracasos
     ..
     cada
     cual
     se
     las
     busca
     como
     pueda-
     @jalvarezflow- @chrisolvido
       orale
       ya
       estas
       en
       aeropuerto
       ,
       bueno
       que
       **tengas**
       buen
       viaje
       y
       ya
       no
       estranaras
       a
       tus
       amigos
       jajajaja
       :-p- @jackybrv
         buenos
         dias
         diosa
         preciosa
         !!
         como
         amanesiste
         ?
         bueno
         que
         **tengas**
         un
         hermoso
         dia
         !!
         tqm
         !- @aybelogando
           eso
           es
           para
           que
           **tengas**
           espacio
           para
           el
           postre
           y
           el
           cafecito
           :p- @thatonegirlbivi
             ni
             **tengas**
             celos- @raydigon
               gracias
               por
               el
               #ff
               de
               ayer
               !!
               que
               **tengas**
               un
               excelente
               fin
               de
               semana
               !- @dockrasovsky
                 gracias
                 por
                 tus
                 bellas
                 palabras
                 me
                 motivan
                 a
                 seguir
                 adelante
                 que
                 **tengas**
                 un
                 exelente
                 jueves
                 ,
                 luz
                 para
                 ty
                 bello- @gabinopadilla
                   que
                   **tengas**
                   un
                   bendecido
                   dia
                   ,
                   gabino
                   .- @erikamurillo
                     hola
                     ..
                     si
                     lo
                     es
                     ..
                     que
                     **tengas**
                     un
                     dia
                     alegre
                     :
                     0
                     )- @ednitanazario
                       mi
                       corazon
                       q
                       **tengas**
                       un
                       lindisimo
                       fin
                       de
                       semana
                       ..
                       q
                       descanses
                       !
                       te
                       quiero
                       muchoo
                       !- @monysor
                         igualmente
                         mony
                         !
                         que
                         **tengas**
                         una
                         maravillosa
                         semana
                         !
                         besos- @ninaamadamia
                           buenos
                           dias
                           **tengas**
                           el
                           dia
                           de
                           hoy
                           ,
                           te
                           deseo
                           de
                           corazon
                           peque- @cynthiaantigua
                             jejeje
                             .
                             que
                             **tengas**
                             un
                             lindo
                             dia
                             !
                             estas
                             en
                             ny
                             oh
                             siges
                             en
                             dr
                             ?- @ponchohdchile
                               pos
                               talves
                               **tengas**
                               suerte- no
                                 significa
                                 que
                                 porque
                                 **tengas**
                                 tu
                                 felicidad
                                 ,
                                 los
                                 otros
                                 no
                                 lo
                                 puedan
                                 tener
                                 :)- @gabrielvoficial
                                   bonito
                                   pensamiento
                                   y
                                   totalmente
                                   cierto
                                   !!
                                   que
                                   **tengas**
                                   buen
                                   dia
                                   !- @soyelmasbuscado
                                     hola
                                     como
                                     te
                                     va
                                     espero
                                     que
                                     **tengas**
                                     un
                                     buen
                                     dia
                                     esta
                                     pero
                                     caliente
                                     lol- @tuliopb
                                       que
                                       **tengas**
                                       fuerza
                                       !- @elvil24
                                         aaww
                                         graciias
                                         chulaa
                                         !!
                                         de
                                         verdad
                                         ke
                                         se
                                         les
                                         agradece
                                         ti
                                         kelo
                                         !!
                                         ke
                                         **tengas**
                                         un
                                         hermoso
                                         dia
                                         lleno
                                         de
                                         #buenavibra
                                         bendiciones
                                         beshitos

## vacaciones

1. @titobertomy
   jajajajajajaja
   have
   fun
   en
   esas
   **vacaciones**
   ;-)- mi
     papa
     es
     un
     loco
     vale
     .
     creo
     que
     voy
     a
     pagar
     todas
     las
     cuentas
     de
     la
     tienda
     en
     **vacaciones**
     .
     y
     con
     -302038912
     bolos
     que
     aporto
     ,
     jajaja- grax
       por
       estas
       **vacaciones**
       dios
       jajaja
       waoo
       estar
       con
       los
       mio
       es
       lo
       mejor- **vacaciones**
         cn
         mi
         chiquitoo
         !!- me
           fui
           de
           **vacaciones**
           ;
           lejos
           de
           los
           amores
           -
           on
           my
           aventura
           shit
           right
           now
           :-d- esto
             de
             estar
             de
             **vacaciones**
             tiene
             que
             ser
             el
             cielo
             .- @nina\_thebest
               :/
               ahi
               .
               no
               encontramo
               a
               guillermina
               ni
               a
               fefita
               .
               supuetamente
               tan
               de
               **vacaciones**
               en
               la
               guasara
               atra
               de
               la
               curva
               de
               pichin- preparandome
                 para
                 ir
                 al
                 aeropuerto
                 pq
                 ya
                 se
                 acabaron
                 las
                 **vacaciones**
                 !!- yo
                   necesito
                   unas
                   **vacaciones**
                   y
                   un
                   superintendente
                   que
                   se
                   haga
                   cargo
                   de
                   darle
                   mantenimiento
                   a
                   mi
                   clinica
                   .
                   apagar
                   el
                   cerebro
                   de
                   preocupaciones- esto
                     de
                     estar
                     en
                     **vacaciones**
                     cuando
                     todo
                     el
                     mundo
                     esta
                     en
                     clase
                     es
                     una
                     mierda- @mashirafael
                       suerte
                       y
                       que
                       tenga
                       unas
                       bonitas
                       **vacaciones**
                       estimado
                       rafael
                       ,
                       recuerde
                       que
                       dios
                       le
                       bendice
                       !- no
                         me
                         gusta
                         **vacaciones**
                         con
                         mi
                         familia
                         .
                         everyone
                         is
                         soo
                         bitchy
                         and
                         hostile
                         .
                         woo
                         !- @jeanessapr
                           perfecto
                           q
                           la
                           pases
                           super
                           en
                           las
                           **vacaciones**
                           y
                           descanses
                           .- muertaa
                             !
                             necesito
                             **vacaciones**
                             asap- @caro\_orellana\_v
                               ah
                               ok
                               amiga
                               yo
                               sigo
                               en
                               huelga
                               jeje
                               :)
                               que
                               rico
                               que
                               estas
                               de
                               mini
                               **vacaciones**
                               :)- @hbolio
                                 de
                                 **vacaciones**
                                 o
                                 vas
                                 a
                                 hacer
                                 verano
                                 ?!- @bestbuymexico
                                   me
                                   compraria
                                   mi
                                   juego
                                   de
                                   resident
                                   evil
                                   operation
                                   raccoon
                                   city
                                   #recompensabby
                                   para
                                   entretenerme
                                   todas
                                   mis
                                   **vacaciones**
                                   ;)- @lovelessnaoko
                                     y
                                     cuando
                                     sales
                                     de
                                     **vacaciones**
                                     ??
                                     siempres
                                     estas
                                     con
                                     mucho
                                     trabajo
                                     y
                                     no
                                     veo
                                     que
                                     tengaa
                                     un
                                     descanso
                                     ~.~- es
                                       q
                                       yo
                                       estoy
                                       de
                                       **vacaciones**
                                       hoy
                                       @joel\_pacheko
                                       @el\_original25- para
                                         mi
                                         nuevo
                                         trabajo
                                         me
                                         exigieron
                                         un
                                         examen
                                         de
                                         alcohol
                                         y
                                         drogas
                                         ..!!
                                         y
                                         gracias
                                         a
                                         la
                                         demora
                                         de
                                         los
                                         resultados
                                         estoy
                                         en
                                         **vacaciones**
                                         vee

## yyaayy

1. **yyaayy**
   i'm
   so
   ready
   for
   this
   lol- i
     had
     a
     good
     day
     @
     work
     now
     on
     my
     way
     home
     to
     see
     my
     favorite
     girl
     ..
     i'm
     off
     2mar
     **yyaayy**- "
       @duckie\_corleone
       :
       @ijaejoe
       **yyaayy**
       !!
       "
       ;)- i'm
         working
         out
         today
         too
         !!
         **yyaayy**- nobody
           panic
           ..
           my
           phone
           problem
           has
           a
           temporary
           solution
           !!
           **yyaayy**
           !!- @v\_marzz
             **yyaayy**
             cuz
             yu
             look
             b-u-t
             full
             :)- @xklusyvjetz
               **yyaayy**
               i
               feel
               loved- @sheplaysgolf
                 ..
                 **yyaayy**
                 !!- @hanslc
                   **yyaayy**
                   congrats
                   ..- @eduardo11
                     **yyaayy**- @blackitaly
                       **yyaayy**
                       ..
                       :)- @theylovebam
                         **yyaayy**
                         my
                         love
                         how
                         iive
                         miissed
                         u
                         so
                         -
                         !- **yyaayy**
                           i
                           got
                           my
                           fafsa
                           done
                           !!
                           #finallyitsover
                           lol- **yyaayy**
                             bout
                             to
                             fuck
                             up
                             moms
                             chilli- sitting
                               at
                               work
                               .
                               there's
                               no
                               heat
                               .
                               **yyaayy**
                               .
                               and
                               **yyaayy**
                               i
                               mean
                               fuck
                               this- @giftgft
                                 **yyaayy**
                                 i'm
                                 soo
                                 happyy
                                 !!
                                 yes
                                 i
                                 can't
                                 park
                                 lol
                                 ..
                                 so
                                 wat- 5
                                   mi
                                   .
                                   done
                                   .
                                   b4
                                   da
                                   rain
                                   came
                                   **yyaayy**
                                   !!- **yyaayy**
                                     the
                                     miss
                                     america
                                     pagaent
                                     is
                                     on
                                     tonight
                                     ..
                                     go
                                     mimi
                                     pabon
                                     !!
                                     mimi
                                     mimi
                                     mimi
                                     mimi
                                     !!- hackkedd
                                       byy
                                       @rinapeyton
                                       **yyaayy**
                                       :]- **yyaayy**
                                         me
                                         and
                                         @nyree17
                                         sonngg
                                         .
                                         best
                                         thing
                                         i
                                         never
                                         had
                                         !

## shee

1. **shee**
   @justlike\_daddy
   shuld
   bbm
   me- **shee**
     got
     dahh
     cakeyy
     and
     all
     latt- shees
       wantinq
       mee
       to
       follow
       all
       thesee
       ruless
       bout
       thesee
       othaa
       bitxhes
       dhat
       be
       at
       mee
       but
       ,
       iss
       **shee**
       playyinqq
       ha
       part
       rytee
       !?- @sullybearrawr
         yes
         !!
         i
         wish
         i
         was
         a
         20s
         gangster
         .
         merh
         **shee**- @mrsthereshegoes
           **shee**
           baacckk
           !!- ah
             no
             she
             not
             ,
             **shee**
             needah
             bring
             her
             ass
             ,
             for
             i
             get
             lazy- @corinaaalesliee
               when
               iss
               **shee**
               getting
               herr
               phone
               baack
               ?- where
                 is
                 **shee**
                 :(- **shee**
                   got
                   it
                   .- ,
                     **shee**
                     needaa
                     cum
                     on
                     manee
                     `- @who\_mike\_jones
                       neva
                       stop
                       tee'n
                       **shee**
                       yu
                       sno- '
                         **shee**
                         saiid
                         **shee**
                         gunna
                         takee
                         mee
                         and
                         dayyaa
                         and
                         rennyy
                         and
                         takee
                         uss
                         outt
                         too
                         eatt
                         sundaiiee
                         '
                         from
                         mycal
                         #tp140402
                         #twiphone- @babykasi
                           **shee**
                           ain't
                           get
                           noo
                           cuddy
                           last
                           niqht
                           that's
                           whyy
                           **shee**
                           madd
                           .
                           lol
                           haha- **shee**
                             did
                             i
                             believee
                             soo
                             @4eva\_smoov3- @jennyd03
                               **shee**
                               can
                               spend
                               anight
                               and
                               sleep
                               in
                               your
                               bed- @free\_man\_juice
                                 ;o
                                 ;
                                 **shee**
                                 a
                                 dogg- @murdaaamook
                                   (
                                   in
                                   my
                                   mind
                                   )
                                   ..
                                   damn
                                   i
                                   gotta
                                   to
                                   work
                                   ..
                                   don't
                                   wanna
                                   fucking
                                   go
                                   ..
                                   looking
                                   though
                                   this
                                   twitter
                                   ..
                                   wait
                                   ..
                                   who
                                   that
                                   ..
                                   **shee**
                                   niccee- bless
                                     you
                                     from
                                     floridart
                                     @hello\_clarisse
                                     so
                                     i
                                     sneezed
                                     and
                                     no1
                                     said
                                     "
                                     god
                                     bless
                                     you
                                     "
                                     -\_-
                                     even
                                     if
                                     you
                                     don't
                                     believe
                                     in
                                     god
                                     ,
                                     just
                                     bless
                                     me
                                     **shee**
                                     !- @vronica\_bby
                                       lol
                                       haa
                                       !!
                                       **shee**
                                       kicked
                                       yewh
                                       out
                                       whutt
                                       uh
                                       douche
                                       baqq
                                       lol
                                       haa
                                       bow
                                       yewh
                                       on
                                       twitter
                                       lol
                                       (:- **shee**
                                         so
                                         adorable
                                         ..
                                         #makayla

## uve

1. @mrzrightt
   @itsgipsybitchh
   ayy
   weve
   been
   here
   question
   is
   where
   **uve**
   been
   at
   ;)- @littlemsdebbie
     live
     like
     **uve**
     never
     been
     hurt
     ..
     something
     to
     contemplate
     on
     ..
     thanks
     mama
     ,
     u
     always
     know
     what
     to
     say
     :-)- @butterfly\_luvly
       and
       when
       ur
       fingers
       still
       smell
       like
       bleach
       after
       two
       showers
       and
       fifty
       handwashes
       u
       know
       **uve**
       done
       a
       good
       job
       lol- @katherinesobol
         ya
         cuz
         **uve**
         been
         calling
         me
         for
         like
         20
         minutes
         off
         a
         blocked
         number
         .
         #urcool- @bomshel
           kel
           by
           the
           look
           in
           ur
           eye
           i
           think
           **uve**
           had
           a
           few
           ;)- @semii\_sweet
             lolszx
             np
             **uve**
             been
             replaced- to
               base
               ur
               judgment
               on
               knowledge
               **uve**
               acquired
               from
               others
               is
               very
               ignorant
               .
               #onthereal- @nolan\_lucas
                 **uve**
                 earned
                 ur
                 follow
                 now- @robynlatoi
                   lmao
                   a
                   lil
                   birdie
                   told
                   me
                   u
                   told
                   them
                   **uve**
                   been
                   married
                   i
                   was
                   like
                   boi
                   stop
                   where
                   they
                   do
                   that
                   at
                   lol- @darrenyoungnxt
                     great
                     match
                     last
                     night
                     man
                     ?
                     but
                     y
                     did
                     they
                     put
                     u
                     bck
                     in
                     nxt
                     if
                     **uve**
                     bn
                     doin
                     matches
                     on
                     superstars
                     ?- @notoriace
                       haha
                       **uve**
                       been
                       to
                       the
                       dp
                       every1
                       talks
                       breeds
                       and
                       whiskey
                       lives
                       like
                       a
                       king
                       and
                       loves
                       the
                       attention
                       !- @yellowkobe
                         **uve**
                         been
                         having
                         a
                         lot
                         of
                         subtweets
                         ..
                         lol
                         ..
                         hope
                         everythings
                         ok
                         friend
                         :)- i
                           kno
                           sometimes
                           i
                           cause
                           u
                           pain
                           .
                           i
                           kno
                           sometimes
                           i
                           may
                           seem
                           vain
                           but
                           baby
                           **uve**
                           through
                           fire
                           n
                           through
                           rain
                           but
                           ull
                           live
                           to
                           love
                           again
                           .- @kallandar13
                             **uve**
                             been
                             hacked
                             .
                             getting
                             spam
                             frm
                             u- headache
                               ,
                               why
                               must
                               u
                               torture
                               me
                               so
                               .
                               **uve**
                               been
                               stalkin
                               me
                               all
                               week
                               .
                               go
                               bother
                               sumone
                               else
                               !- @tasha592
                                 i
                                 came
                                 down
                                 twice
                                 last
                                 week
                                 ..
                                 **uve**
                                 been
                                 mia
                                 from
                                 there
                                 !!
                                 il
                                 try
                                 and
                                 come
                                 tmr- my
                                   lifes
                                   like
                                   a
                                   movie
                                   and
                                   momma
                                   keep
                                   falln
                                   in
                                   lu
                                   **uve**
                                   #ooooowwwww
                                   #lyrics
                                   #free
                                   max
                                   b- @avi\_sodmg
                                     oh
                                     no
                                     lolz
                                     if
                                     it
                                     say
                                     **uve**
                                     been
                                     kicked
                                     does
                                     that
                                     mean
                                     i
                                     cant
                                     talk
                                     anymore
                                     till
                                     tomorrow
                                     lolz- if
                                       u
                                       wanna
                                       go
                                       to
                                       sleep
                                       after
                                       **uve**
                                       eaten
                                       ..
                                       that's
                                       bcuz
                                       u
                                       just
                                       had
                                       some
                                       #goodfood- @mvpete96
                                         ya
                                         pretty
                                         unlucky
                                         to
                                         hit
                                         the
                                         post
                                         .
                                         twice
                                         even
                                         .
                                         **uve**
                                         scored
                                         a
                                         goal
                                         before
                                         u
                                         know
                                         what
                                         thats
                                         like

## u've

1. @itsjustayo
   **u've**
   officially
   been
   chopped
   nd
   screwed
   ..- @2great2bavg1901
     nite
     boo
     **u've**
     been
     mia- life
       lesson
       :
       how
       can
       you
       say
       ya
       mama's
       fried
       chicken
       is
       the
       best
       ,
       if
       that's
       the
       only
       fried
       chicken
       **u've**
       tasted
       !- @kidsupreme87
         ..
         well
         damn
         gucci
         !
         its
         kennyw2
         .
         even
         tho
         **u've**
         punctured
         my
         heart
         i
         still
         love
         u
         ..
         and
         w4l
         productions
         are
         still
         on
         .- **u've**
           been
           benched
           !!
           #threewordstories- @tstashwick
             please
             tell
             me
             **u've**
             seen
             how
             the
             veteran's
             administration
             provides
             health
             care
             .
             trust
             me
             ,
             gov't
             health
             care
             is
             not
             wht
             u
             wnt
             .- **u've**
               captured
               my
               heart
               ,
               my
               treasure
               .
               you
               looked
               at
               me
               ,
               &
               i
               fell
               in
               love
               .
               one
               look
               my
               way
               and
               i
               was
               hopelessly
               in
               love
               !
               -songs
               of
               solomon
               4:9- @aubreyoday
                 will
                 u
                 please
                 come
                 to
                 london
                 soon
                 ?
                 maybe
                 for
                 a
                 few
                 night
                 clubs
                 gigs
                 or
                 somethin
                 !
                 **u've**
                 soo
                 many
                 fans
                 here
                 .- @missmaryj
                   ur
                   vision
                   is
                   gettin
                   worse
                   bcuz
                   **u've**
                   needed
                   glasses
                   4
                   years
                   &
                   havent
                   seen
                   a
                   doctor
                   #whodoesthat
                   ~~>
                   yep
                   ,
                   #guiltyascharged- and
                     my
                     mom
                     wonders
                     why
                     i
                     don't
                     have
                     any
                     f'in
                     respect
                     for
                     her
                     .
                     when
                     u
                     start
                     trash
                     talking
                     someone
                     who
                     i
                     care
                     about
                     .
                     **u've**
                     crossed
                     the
                     line- @donniewahlberg
                       no
                       thank
                       you
                       lover
                       !
                       u
                       now
                       own
                       my
                       heart
                       ,
                       &
                       my
                       body
                       &
                       my
                       soul
                       **u've**
                       always
                       had
                       it
                       &
                       u
                       always
                       will
                       !
                       i
                       love
                       u
                       !- @idomeesh
                         lol
                         yea
                         i
                         was
                         ,
                         u
                         jealous
                         michelle
                         ?
                         i'm
                         sure
                         its
                         been
                         a
                         while
                         since
                         **u've**
                         had
                         a
                         good
                         grilled
                         cheese
                         !- #imthetypeofperson
                           that
                           you
                           gotta
                           wrk
                           hard
                           2
                           get
                           me
                           2
                           trust
                           u
                           once
                           **u've**
                           crushed
                           it- it's
                             midday
                             and
                             i
                             haven't
                             had
                             a
                             fruit
                             smoothie
                             or
                             starbucks
                             yet
                             !
                             **u've**
                             been
                             warned
                             ,
                             lol- "
                               a
                               gud
                               rule
                               of
                               thumb
                               is
                               if
                               **u've**
                               made
                               it
                               to
                               35
                               and
                               ur
                               job
                               still
                               requires
                               u
                               2
                               wear
                               a
                               name
                               tag
                               ,
                               **u've**
                               made
                               a
                               serious
                               vocational
                               error
                               .
                               d
                               .
                               miller- im
                                 a
                                 good
                                 mood
                                 lets
                                 see
                                 if
                                 **u've**
                                 got
                                 the
                                 right
                                 stuff
                                 :p- @jon\_huertas
                                   my
                                   just
                                   joking
                                   ??
                                   "
                                   now
                                   that
                                   we
                                   know
                                   **u've**
                                   always
                                   wanted
                                   2
                                   moonlite
                                   4
                                   free
                                   as
                                   a
                                   porn
                                   star
                                   .
                                   why
                                   pick
                                   an
                                   unknown
                                   like
                                   @southbelle
                                   ?
                                   "- actin
                                     is
                                     about
                                     honesty
                                     ..
                                     if
                                     u
                                     can
                                     fake
                                     dat
                                     ,
                                     **u've**
                                     got
                                     it
                                     made
                                     ..
                                     foonies- listening
                                       to
                                       smokey
                                       robinson
                                       and
                                       the
                                       miracles
                                       -
                                       **u've**
                                       really
                                       gotta
                                       hold
                                       on
                                       me
                                       ..- @lalaalena
                                         hahahahahaha
                                         **u've**
                                         officailly
                                         #geekedme
                                         2nite
                                         wit
                                         ur
                                         thugs
                                         and
                                         "
                                         #todolist
                                         "
                                         i
                                         mite
                                         have
                                         to
                                         get
                                         one
                                         of
                                         those
                                         haha
                                         !

## jut

1. people
   who
   are
   **jut**
   getting
   hip
   to
   @theweekndxo
   <<- @jayde\_nicole
     wow
     **jut**
     first
     5
     minutes
     seems
     so
     staged
     ..
     not
     sure
     if
     i'll
     keep
     watching
     .- @wingedparadox
       oh
       my
       god
       .
       give
       me
       .
       i
       will
       pay
       you
       $19.90
       for
       that
       !
       :d
       **jut**
       keeding
       ?
       yes
       .- **jut**
         woke
         up- i
           wish
           i
           was
           so
           rich
           i
           could
           **jut**
           blow
           my
           money
           on
           pointless
           random
           stuff
           .- **jut**
             finished
             writing
             my
             liner
             notes
             for
             dionne
             warwicks
             new
             album
             coming
             out
             in
             march
             2011
             ..- martin
               prado
               i
               will
               love
               you
               forever
               if
               you
               will
               **jut**
               end
               this
               gam
               right
               here
               i
               have
               to
               write
               a
               paper- **jut**
                 finished
                 cheating
                 my
                 ass
                 off- @tmt\_fucwatuhurd
                   ..
                   yu
                   can
                   havee
                   dat
                   ju
                   **jut**
                   gottha
                   act
                   like
                   ju
                   dew- **jut**
                     saw
                     a
                     lady
                     with
                     two
                     large
                     dogs
                     carrying
                     a
                     huge
                     bag
                     of
                     shit
                     lol
                     i
                     will
                     never
                     pick
                     my
                     dogs
                     shit
                     up
                     for
                     that
                     reason
                     hahaha- cuz
                       i
                       won't
                       forgive
                       i'll
                       **jut**
                       forget
                       you
                       lived
                       !- sometimes
                         you
                         **jut**
                         gotta
                         walk
                         in
                         the
                         room
                         smack
                         her
                         on
                         the
                         ass
                         and
                         whisper
                         in
                         her
                         ear
                         "
                         drop
                         them
                         draws
                         girl
                         "- on
                           facebook
                           scrolling
                           down
                           \*
                           me
                           :
                           "
                           idk
                           how
                           many
                           times
                           i
                           **jut**
                           saw
                           that
                           as
                           someone's
                           status
                           ..- #thoughtsduringsex
                             man
                             i
                             hope
                             he
                             dnt
                             pull
                             my
                             hair
                             this
                             shit
                             needa
                             be
                             re-adjusted
                             first
                             ..
                             aye
                             i
                             **jut**
                             know
                             he
                             taking
                             me
                             2
                             the
                             salon
                             in
                             the
                             morn- damn
                               ,
                               and
                               tonight
                               nothing's
                               on
                               my
                               mind
                               ,
                               i'm
                               not
                               **jut**
                               not
                               fucking
                               tired
                               ..
                               i
                               wanna
                               #sleep- @paperandvinyl
                                 **jut**
                                 had
                                 a
                                 seizure
                                 in
                                 line
                                 to
                                 get
                                 into
                                 the
                                 show
                                 .
                                 shit
                                 is
                                 fucked
                                 up- @purple\_diesel
                                   ok
                                   im
                                   sorry
                                   ill
                                   **jut**
                                   call
                                   u
                                   names
                                   in
                                   person
                                   then- :o
                                     **jut**
                                     remember
                                     ihave
                                     some
                                     weed
                                     left
                                     (:
                                     hmm
                                     take
                                     a
                                     walk
                                     and
                                     explore
                                     ??
                                     lol
                                     maybee
                                     !- supposed
                                       to
                                       be
                                       studying
                                       and
                                       i
                                       **jut**
                                       watched
                                       goodfellas
                                       from
                                       beginning
                                       to
                                       end
                                       in
                                       bed- i
                                         really
                                         **jut**
                                         busted
                                         out
                                         laughing
                                         in
                                         class
                                         ,
                                         laughing
                                         at
                                         bird
                                         lame
                                         ass

## jux

1. i
   be
   lyin
   **jux**
   to
   hang
   up
   hell- @veevelasquez
     thts
     how
     i
     feel
     but
     **jux**
     deleted
     all
     mi
     numbers
     ?
     how
     r
     yu
     been
     ?- das
       riteert
       @shawtymello
       :
       me
       &&
       @mr\_plzurneedz
       was
       **jux**
       playin
       ppl
       !
       that's
       my
       babe
       !
       =)- i'm
         **jux**
         sayin
         tho
         lmao- @ohsopretty24
           lol
           ..
           i'm
           saying
           ..
           that
           super
           baller
           lol
           .
           i
           **jux**
           went
           to
           my
           moms
           and
           spent
           time
           with
           my
           family
           ..
           had
           fun
           and
           it
           was
           overdue- i
             want
             a
             new
             phone
             **jux**
             for
             da
             hell
             of
             it- **jux**
               gt
               from
               church- **jux**
                 made
                 it
                 2
                 dhaa
                 crib
                 frm
                 practice
                 !- teacher
                   **jux**
                   said
                   niggas
                   don't
                   kno
                   hist
                   !
                   africans
                   fought
                   africans
                   and
                   enslaved
                   themselves
                   #bitchplease
                   #wellyoumayberight
                   niggas- @thatothergirl\_b
                     leqit
                     2
                     sleep
                     lolx
                     im
                     **jux**
                     playinq
                     britt- .
                       #oomf
                       **jux**
                       told
                       me
                       to
                       hush
                       #whytho- i
                         **jux**
                         dnt
                         affiliate
                         wit
                         extra
                         fake
                         synthetic
                         ..
                         artificial
                         ass
                         bytches
                         ..
                         lmao- **jux**
                           talk
                           to
                           the
                           da
                           college
                           coach
                           #winning- im
                             bout
                             to
                             #letgo
                             #oomf
                             n
                             **jux**
                             #forgetit
                             #thrutryn
                             ..
                             but
                             #anyway
                             still
                             with
                             the
                             #kuzzos
                             .- mann
                               mi
                               ankle
                               is
                               hurtin
                               heellaa
                               badd
                               smh
                               see
                               basketball
                               is
                               **jux**
                               not
                               mi
                               sport
                               .
                               .
                               #realtalk- **jux**
                                 came
                                 4rm
                                 the
                                 game
                                 ,
                                 layn
                                 down
                                 had
                                 on
                                 a
                                 fresh
                                 v-neck
                                 had
                                 to
                                 take
                                 it
                                 off
                                 ,
                                 cuz
                                 i
                                 might
                                 **jux**
                                 fall
                                 asleep
                                 !- them
                                   hoes
                                   **jux**
                                   got
                                   yah
                                   head
                                   gne- **jux**
                                     letting
                                     you
                                     know- dey
                                       really
                                       **jux**
                                       showd
                                       dey
                                       ghetto
                                       side
                                       lol- i
                                         **jux**
                                         tweet
                                         wats
                                         on
                                         my
                                         mind

## jus

1. @lbugg91
   b
   quiet
   ,
   dat
   song
   was
   **jus**
   on
   my
   mind
   ..
   vibein
   to
   da
   oldies
   lol- @proudyb
     yeahh
     i
     knoo
     lol
     ..
     dunt
     u
     **jus**
     love
     mah
     #ihateitwhen
     trending
     topic- i
       need
       new
       team
       members
       really
       i
       **jus**
       need
       a
       new
       thug
       ..
       lmao- @ohnoitsterr
         dead
         ass
         serious
         ..
         i
         **jus**
         called
         611
         and
         they
         said
         i
         gotta
         full
         upgrade
         and
         everything
         ..
         so
         i
         coulda
         got
         it
         for
         like
         80
         bucks- i
           feel
           bad
           ..
           there's
           a
           group
           of
           charles
           school
           students
           by
           the
           window
           n
           all
           of
           a
           sudden
           1of
           em
           **jus**
           starts
           breakin
           dwn
           ..
           i
           cnt
           imagine
           tht
           pain- bt
             some
             shit
             is
             **jus**
             uncalled
             for- #ff
               my
               nigga
               @shablack
               **jus**
               cuz
               he'll
               put
               a
               bitch
               in
               its
               place
               2point2
               second
               .- and
                 i
                 didn't
                 think
                 i
                 was
                 ready
                 to
                 let
                 go
                 but
                 i'm
                 just
                 so
                 unhappy
                 and
                 your
                 **jus**
                 the
                 worst
                 person
                 to
                 try
                 to
                 be
                 with- @suchafuccnlady
                   **jus**
                   chillin
                   tryna
                   make
                   dis
                   cash
                   sum
                   how
                   ,
                   wud
                   up
                   ur
                   way
                   ??- @manipretty
                     hell
                     no
                     ..
                     **jus**
                     was
                     seeing
                     u
                     beening
                     cheap
                     lol- my
                       dawg
                       **jus**
                       called
                       me
                       ..
                       dem
                       pills
                       gottem
                       chirpin
                       ..
                       ask
                       em
                       wat
                       he
                       doin
                       said
                       "
                       shyly
                       **jus**
                       lurrkin
                       "
                       #goontweet
                       #fb- @jsermo
                         i
                         kno
                         i'm
                         **jus**
                         givin
                         ya
                         shit
                         bout
                         it
                         lol- i
                           absolutely
                           hate
                           liars
                           don't
                           fuccn
                           lie
                           dude
                           u
                           **jus**
                           make
                           yourself
                           look
                           dumb- niggaz
                             be
                             wearin
                             snap
                             backs
                             **jus**
                             cuz
                             lol- #epicfail
                               you
                               just
                               dnt
                               do
                               shit
                               like
                               that
                               ..
                               i
                               cud
                               have
                               been
                               finishing
                               my
                               work
                               ..
                               **jus**
                               wasted
                               30
                               mins
                               of
                               my
                               life- @msmuppet
                                 lol
                                 im
                                 **jus**
                                 tellin
                                 u
                                 !- #dontactlikeyounever
                                   made
                                   a
                                   bum
                                   do
                                   sum
                                   funny
                                   shyt
                                   **jus**
                                   to
                                   give
                                   them
                                   a
                                   quarter
                                   n
                                   the
                                   end
                                   !- @im\_sucha\_lady
                                     oh
                                     ok
                                     **jus**
                                     askin
                                     care
                                     bout
                                     my
                                     followers- @juztlisa
                                       hell
                                       no
                                       !
                                       i
                                       **jus**
                                       wanna
                                       be
                                       cunt
                                       lol- somebody
                                         **jus**
                                         told
                                         me
                                         i
                                         was
                                         a
                                         fool
                                         for
                                         messin
                                         up
                                         wit
                                         @\_\_\_\_\_\_\_\_
                                         ..
                                         lol
                                         but
                                         i
                                         didnt
                                         mess
                                         up
                                         ..
                                         it
                                         **jus**
                                         didnt
                                         work
                                         out

## jsut

1. it
   feels
   like
   i
   **jsut**
   broke
   my
   ear- @xdeadfredx
     and
     i
     was
     like
     '
     it's
     fine
     ..
     '
     because
     really
     the
     damage
     is
     done
     ,
     they
     all
     got
     the
     millions
     of
     notifications
     and
     i
     **jsut**
     look- i
       **jsut**
       chowwedd
       thee
       fuckk
       downn
       mann- is
         **jsut**
         ready
         to
         start
         over
         ..
         drama
         free
         .
         can
         that
         possibly
         happen
         ?- **jsut**
           lost
           cell
           service
           in
           tampa
           ,
           anyone
           else
           ?
           #att- @billionaire\_b
             **jsut**
             chillin
             shopping
             and
             stuff- **jsut**
               gettin
               back
               4rm
               applebee's
               ..
               i
               full
               up
               as
               they
               would
               say
               in
               charleston
               ..
               skype
               me
               im
               bored
               brittanybostic- @gbc001
                 idk
                 bra
                 im
                 **jsut**
                 tweeting
                 everything
                 that's
                 coming
                 to
                 mind
                 .- @adoired
                   **jsut**
                   started
                   singing
                   ..
                   \*
                   insert
                   head
                   phones
                   \*- i
                     will
                     ,
                     in
                     most
                     probability
                     ,
                     regret
                     this
                     words
                     soon
                     but
                     i
                     really
                     **jsut**
                     made
                     pai
                     gow
                     poker
                     my
                     bitch
                     .- @mikeymcfly
                       me
                       too
                       and
                       i
                       **jsut**
                       got
                       to
                       the
                       office
                       ..
                       smh- @mzauth3ntik
                         hey
                         ..
                         my
                         bad
                         **jsut**
                         saw
                         ur
                         tweet
                         .
                         i'm
                         doing
                         good
                         though
                         how
                         about
                         you
                         ..??- @opeezy09
                           damn
                           it
                           looks
                           like
                           you
                           look
                           **jsut**
                           like
                           your
                           mom
                           .
                           shit
                           is
                           mad
                           blurry
                           but
                           .
                           .
                           looks
                           like
                           it
                           .- @runlikeamug
                             **jsut**
                             the
                             basics
                             of
                             being
                             a
                             good
                             human
                             being
                             in
                             my
                             estimation
                             .
                             seems
                             most
                             people
                             do
                             not
                             agree
                             with
                             me- did
                               i
                               **jsut**
                               make
                               headway
                               at
                               work
                               and
                               leave
                               a
                               meeting
                               with
                               a
                               smile
                               ?
                               #crapsomethingbadisgonnahappen- realize
                                 that
                                 i'm
                                 human
                                 and
                                 i
                                 have
                                 **jsut**
                                 as
                                 many
                                 insecurities
                                 as
                                 the
                                 next
                                 chick
                                 .
                                 .
                                 .- mann
                                   so
                                   my
                                   mama
                                   **jsut**
                                   wake
                                   up
                                   at
                                   4
                                   2nite
                                   an
                                   get
                                   back
                                   on
                                   the
                                   road
                                   shidd
                                   i
                                   might
                                   as
                                   stay
                                   up- @21streetcoffee
                                     maybe
                                     ..
                                     but
                                     also
                                     many
                                     people
                                     don't
                                     **jsut**
                                     eat
                                     ketchup
                                     alone
                                     ,
                                     they
                                     add
                                     a
                                     hot
                                     dog
                                     to
                                     it
                                     to
                                     complete
                                     the
                                     flavor
                                     profile
                                     :-)- @michaelferrari
                                       **jsut**
                                       wave
                                       your
                                       hand
                                       and
                                       say
                                       "
                                       this
                                       is
                                       the
                                       deal
                                       you're
                                       looking
                                       for
                                       .
                                       "
                                       #jedimindtrick- @thelongsilence
                                         well
                                         you
                                         have
                                         to
                                         wake
                                         up
                                         to
                                         feed
                                         harlow
                                         soon
                                         so
                                         why
                                         not
                                         **jsut**
                                         stay
                                         up
                                         ?

## iaint

1. bidch
   **iaint**
   da
   1
   to
   be
   fuked
   with
   .- ineeda
     nap
     when
     iget
     off
     ..
     but
     **iaint**
     fukkn
     up
     my
     saturday
     lol- imiss
       @homewreckin\_101
       **iaint**
       talked
       to
       her
       in
       a
       whille- **iaint**
         on
         no
         sports
         team
         ..
         but
         ya
         nigga
         ballin
         '
         ..- istill
           don't
           understand
           my
           tumblr
           page
           **iaint**
           been
           on
           there
           since
           imade
           my
           1st
           4
           post- its
             some
             twitter
             love
             connections
             goin
             on
             an
             **iaint**
             sayin
             no
             names
             !!
             ha- that's
               why
               **iaint**
               gget
               on
               today- @jreezy\_11
                 yu
                 knw
                 **iaint**
                 scared
                 ..- i
                   was
                   bout
                   2
                   say
                   **iaint**
                   see
                   u
                   in
                   a
                   minute
                   ..
                   buhh
                   i
                   jus
                   saw
                   u
                   dha
                   otha
                   dai
                   :-)
                   !
                   @imake\_emsquirt- damn
                     u
                     kno
                     who
                     went
                     silent
                     **iaint**
                     seen
                     #teamhnf
                     on
                     my
                     tl
                     @
                     all
                     lmao
                     what's
                     up
                     with
                     that
                     iguess
                     its
                     #teamrmf- @beasleybby
                       ion
                       knoww
                       ..
                       **iaint**
                       know
                       she
                       hoop- ugh
                         ..
                         but
                         **iaint**
                         going
                         to
                         sleep
                         yet- ion
                           really
                           vibe
                           wit
                           a
                           lot
                           a
                           dese
                           niggas
                           ,
                           **iaint**
                           really
                           into
                           givin
                           more
                           den
                           one
                           chance
                           girl
                           ,
                           ialready
                           tried
                           wit
                           alot
                           a
                           dese
                           niggas
                           fuck
                           that
                           !- mad
                             **iaint**
                             get
                             a
                             kiss
                             thou
                             ..- **iaint**
                               tryna
                               find
                               out
                               eitha- @bjthamanson
                                 lmaoo
                                 **iaint**
                                 even
                                 peep- tss
                                   ;
                                   **iaint**
                                   even
                                   doo
                                   all
                                   mhyproject'ss
                                   thoo
                                   .- @kawaii\_renee
                                     i
                                     feel
                                     dat
                                     ,
                                     do
                                     ya
                                     thang
                                     ,
                                     **iaint**
                                     hatin
                                     #boosie- a
                                       nigga
                                       really
                                       mad
                                       that
                                       **iaint**
                                       never
                                       rolled
                                       my
                                       own
                                       blunt
                                       .
                                       <<- @williegotemopen
                                         cuz
                                         **iaint**
                                         get
                                         ah
                                         permission
                                         slip
                                         &
                                         **iaint**
                                         wanna
                                         go
                                         ;
                                         how
                                         was
                                         it
                                         ?

## ain't

1. '
   you
   say
   you
   on
   your
   period
   ,
   i
   **ain't**
   hearing
   it
   ,
   put
   a
   rubber
   on
   i
   **ain't**
   fearing
   it
   '- @miss\_agem
     one
     say
     you'll
     realize
     change
     is
     best
     ..
     u
     **ain't**
     learn
     shit
     from
     barack
     ?
     lol- @ososwishersweet
       @tweetme\_\_\_
       stop
       playing
       wur
       me
       like
       yo
       life
       **ain't**
       real- wow
         tj
         ford
         retired
         smh
         neck
         injuries
         **ain't**
         nothing
         to
         play
         with
         hope
         his
         health
         get
         back
         100%- downtown
           frisco
           is
           crazy
           with
           shoppers
           .
           **ain't**
           trying
           to
           hey
           invved- if
             you
             and
             giving
             100
             i
             promise
             i
             **ain't**
             fucking
             with
             it
             ,- damn
               class
               already
               ..
               i
               **ain't**
               get
               no
               nap
               ..
               fuck
               !- @goonassross
                 you
                 **ain't**
                 know
                 ?
                 check
                 what
                 he
                 do
                 to
                 ray
                 allen
                 !- my
                   chick
                   @sandybernadette
                   **ain't**
                   thirsty
                   she
                   hungry
                   !!
                   lol
                   hahahaha- #thingscheaterssay
                     oh
                     my
                     homeboy
                     **ain't**
                     have
                     his
                     phone
                     last
                     night
                     so
                     i
                     had
                     to
                     save
                     this
                     girl
                     phone
                     number
                     for
                     him
                     in
                     my
                     phone- @barnonefadeall
                       u
                       **ain't**
                       never
                       lie
                       !
                       i
                       never
                       tweet
                       from
                       the
                       pc
                       n
                       then
                       to
                       b
                       tweetn
                       all
                       dam
                       day
                       via
                       web
                       ..
                       #nawimstraight- saleem
                         betta
                         cutt
                         dat
                         attitude
                         he
                         got
                         rite
                         now
                         cus
                         i
                         **ain't**
                         do
                         shit
                         to
                         him
                         !- it
                           **ain't**
                           easy
                           being
                           cheesy- &&
                             it
                             **ain't**
                             a
                             strip
                             club
                             .,
                             if
                             they
                             **ain't**
                             showing
                             pussy
                             !.
                             @yolo\_28- the
                               things
                               people
                               do
                               when
                               they
                               nigga
                               **ain't**
                               around
                               ..
                               pure
                               foolery
                               just
                               be
                               single- s/o
                                 to
                                 my
                                 fav
                                 og
                                 @joeycrackkkk
                                 **ain't**
                                 talk
                                 to
                                 her
                                 in
                                 a
                                 min- @caramelkisses2u
                                   **ain't**
                                   ntn
                                   chillen
                                   how
                                   yu
                                   doing- it's
                                     raining
                                     and
                                     i
                                     **ain't**
                                     wit
                                     bay
                                     ..- dat
                                       cush
                                       put
                                       me
                                       on
                                       my
                                       ass
                                       last
                                       night
                                       i
                                       **ain't**
                                       eem
                                       gone
                                       lie- "
                                         @simplywhitley
                                         :
                                         @missmiinephew20
                                         them
                                         fukkn
                                         naps
                                         them
                                         **ain't**
                                         waves
                                         "
                                         lol

## aint

1. @santhaban
   na
   i
   **aint**
   fuckin
   wit
   @t\_thoughts
   either
   i
   asked
   him
   to
   put
   me
   on
   with
   a
   girl
   and
   he
   didn't
   so
   eff
   both
   yal
   #blockers- @real\_ambition1
     lol
     tht
     u
     might
     expose
     tht
     boy
     he
     **aint**
     about
     tht
     life
     ..
     yet
     !!- @trapniggaflee
       yoo
       ya
       bb
       is
       trash
       i
       wrote
       u
       back
       earlier
       and
       it
       still
       **aint**
       go
       thru
       !!
       wtf- @tangierenee
         suppose
         too
         waiting
         on
         my
         boy
         money
         to
         get
         right
         cuz
         i
         **aint**
         paying
         for
         everything- @donniewahlberg
           i
           just
           took
           a
           big
           breath
           ,
           that
           **aint**
           oxygen
           ,
           what
           is
           it
           ?!!- omfg
             the
             o-line
             **aint**
             have
             shit
             to
             do
             with
             that
             !!- bou
               ta
               finish
               this
               movie
               and
               sleep
               ,
               dnt
               have
               the
               energy
               to
               cook
               i
               **aint**
               havin
               dinner
               tonite
               ,
               will
               attend
               to
               my
               tummy
               tomoro- @ladiej135
                 lol
                 .
                 naw
                 if
                 all
                 goes
                 well
                 i'm
                 outta
                 there
                 next
                 semester
                 .
                 if
                 i
                 don't
                 have
                 to
                 stay
                 i
                 **aint**
                 .
                 lol
                 .
                 ppl
                 b
                 playin
                 .
                 i'm
                 n
                 and
                 out- @bricetonmcnair
                   i
                   asked
                   ha
                   2
                   ask
                   you
                   2
                   put
                   **aint**
                   no
                   way
                   round
                   it
                   and
                   boo
                   by
                   tity
                   boy
                   ..
                   please
                   ?
                   \*
                   hopefull
                   voice
                   \*- @itskiki\_mmm
                     i
                     would
                     have
                     u
                     but
                     im
                     sure
                     u
                     **aint**
                     havin
                     it- champ
                       **aint**
                       lying
                       down
                       son
                       #evo2k- #ilikeduuntil
                         u
                         told
                         me
                         ya
                         last
                         boyfriend
                         liked
                         it
                         !!?!?!
                         #pause
                         ..
                         sorry
                         shawty
                         that
                         **aint**
                         my
                         thang
                         !- i
                           hate
                           girls
                           that
                           say
                           bro
                           like
                           nah
                           that
                           **aint**
                           cool
                           or
                           my
                           dude
                           that
                           **aint**
                           for
                           girls
                           im
                           sorry
                           #turnoff- these
                             buffalo
                             wings
                             slick
                             dun
                             gave
                             me
                             heartburn
                             ,
                             and
                             this
                             smirnoff
                             **aint**
                             helpin
                             !
                             lol
                             @chels\_27\_love
                             want
                             one
                             ?- @ebebonly1love
                               hell
                               na
                               .
                               imma
                               be
                               all
                               about
                               it
                               in
                               a
                               cpl
                               months
                               but
                               until
                               then
                               if
                               it
                               **aint**
                               a
                               hot
                               tub
                               i
                               **aint**
                               splashin- @mizblessed
                                 u
                                 really
                                 gotta
                                 stop
                                 dis
                                 celtic
                                 talk
                                 or
                                 we
                                 **aint**
                                 friends- niggas
                                   made
                                   it
                                   seem
                                   like
                                   bbm
                                   was
                                   groundbreaking
                                   and
                                   shi
                                   t.t
                                   hat
                                   shit
                                   **aint**
                                   nuhtin
                                   but
                                   aim
                                   on
                                   a
                                   bb
                                   .
                                   smfh
                                   .
                                   niggas
                                   always
                                   exaggerating
                                   about
                                   shit
                                   .- shit
                                     just
                                     pissed
                                     me
                                     off
                                     .
                                     like
                                     its
                                     my
                                     fault
                                     you
                                     **aint**
                                     got
                                     a
                                     back
                                     bone
                                     ..
                                     naw
                                     it
                                     **aint**
                                     ,
                                     i
                                     got
                                     plenty
                                     .
                                     and
                                     you
                                     know
                                     i
                                     will
                                     die
                                     for
                                     mine- @cloud9\_smurf
                                       u
                                       crazy
                                       but
                                       u
                                       **aint**
                                       lien
                                       co0llie
                                       the
                                       sweety
                                       !!- #thingscheatersay
                                         i
                                         **aint**
                                         gone
                                         do
                                         it
                                         no
                                         more
                                         !

## ainn

1. this
   year
   awards
   was
   whack
   .
   &
   it
   **ainn**
   even
   over
   withh
   yet
   !- i
     **ainn**
     been
     to
     the
     zoo
     ,
     since
     i
     was
     9
     >>- @swaaq\_od
       **ainn**
       nothing
       showing
       buhh
       my
       stomach
       .- beforee
         i
         give
         youu
         the
         keyys
         lett
         youu
         know
         i
         **ainn**
         '
         playnn
         but
         ,
         before
         i
         jumpp
         out
         thee
         window
         whts
         yaa
         '
         namee
         ?- nicckas
           '
           **ainn**
           real
           at
           all- so
             she
             **ainn**
             see
             the
             tweet
             ?
             pmo
             !- i
               **ainn**
               '
               gone
               lie
               that
               was
               my
               show
               .
               .- @bosskiemaa\_moe
                 lolss
                 ,
                 nunn
                 juss
                 came
                 fromm
                 the
                 parrtyy
                 ;
                 omw
                 too
                 the
                 bbx
                 w
                 @kaygotdht\_
                 why
                 youu
                 **ainn**
                 '
                 comee- **ainn**
                   ate
                   shxd
                   all
                   dayy
                   .
                   thesee
                   chipss
                   so
                   good
                   .- @the3rdhamilton
                     i
                     **ainn**
                     even
                     know
                     we
                     had
                     it
                     tomorrow- @x\_kaleighmom\_
                       i
                       **ainn**
                       hushing
                       .
                       i
                       did
                       nothing
                       wrong
                       .
                       lol- seems
                         like
                         dont
                         nobody
                         give
                         a
                         damn
                         ,
                         you
                         can
                         sayy
                         you
                         care
                         all
                         day
                         but
                         words
                         mean
                         nothing
                         if
                         you
                         **ainn**
                         showinn
                         no
                         actions
                         #wifetweet- my
                           tv
                           on
                           but
                           i
                           **ainn**
                           watchn
                           it- i
                             **ainn**
                             '
                             even
                             have
                             to
                             wait
                             to
                             the
                             upper
                             grades
                             for
                             the
                             bitch
                             to
                             shrink
                             ..
                             \*
                             shrugs
                             \*- @geeripdeiante
                               nyny
                               so
                               they
                               sayin
                               future
                               **ainn**
                               comin
                               !- @djtsmoove
                                 i
                                 would
                                 but
                                 **ainn**
                                 much
                                 of
                                 a
                                 freestyler
                                 ,
                                 cant
                                 embarrass
                                 myself- y'all
                                   over
                                   here
                                   arguing
                                   about
                                   if
                                   he
                                   dead
                                   or
                                   not
                                   !
                                   like
                                   he
                                   **ainn**
                                   '
                                   worried
                                   about
                                   youu
                                   .
                                   you
                                   arguing
                                   over
                                   somebody
                                   who
                                   don't
                                   even
                                   know
                                   you
                                   exist- **ainn**
                                     have
                                     fast
                                     food
                                     in
                                     a
                                     month
                                     #smh- im
                                       not
                                       sleepy
                                       ,
                                       but
                                       **ainn**
                                       got
                                       nobody
                                       to
                                       keep
                                       me
                                       up
                                       ..
                                       wahh- **ainn**
                                         even
                                         gon
                                         trip
                                         !!

## aiint

1. @raylovessyou
   myy
   mom
   **aiint**
   lettiin
   mehh
   talkk
   on
   thee
   cellular
   riiqht
   noww
   &&
   she
   liimiitiinqq
   myy
   tiimee
   on
   thee
   pc
   :/
   smh- @mike\_2\_wavy
     lol
     ii
     **aiint**
     tryna
     stay
     hea
     neiither
     lml
     so
     goo
     far
     dnt
     wanna
     see
     yu
     (
     x\_\_x
     )- @tharealdollas
       watz
       good
       dame
       ..
       **aiint**
       hitchu
       up
       iin
       a
       miinutee- @skuuur\_thatstye
         @simplyy\_gawjuss
         yall
         **aiint**
         do
         shit- @cirocinmysystem
           wee
           **aiint**
           even
           do
           shiit
           ..- @luv\_kiki
             yea
             you
             qot
             iit
             riiqht
             but
             you
             **aiint**
             sayy
             my
             bday
             >:o
             !!- @brandoncici
               i
               swear
               i
               didn't
               get
               the
               missed
               call
               so
               take
               the
               screen
               shot
               !
               i
               **aiint**
               caree
               !
               duhh
               !
               :p- @ayoxlele
                 lol
                 ..
                 but
                 **aiint**
                 nobody
                 wanna
                 hear
                 that
                 tho
                 .
                 and
                 u
                 said
                 it
                 so
                 loud
                 everybody
                 behind
                 u
                 turned
                 around
                 .
                 but
                 i
                 **aiint**
                 say
                 nuttin
                 tho
                 .
                 lol- @boogzdaaddict
                   i
                   **aiint**
                   been
                   on
                   the
                   shiit
                   at
                   all
                   really- @misztastytaurus
                     tht
                     cereal
                     iis
                     good
                     tho
                     ii
                     **aiint**
                     gonna
                     front
                     !!
                     he
                     tryna
                     say
                     she
                     aste
                     good
                     or
                     somethiing- @i\_stack\_carrots
                       lmaoo
                       wow
                       u
                       **aiint**
                       got
                       noo
                       cum
                       baqq
                       .
                       u
                       gotta
                       thiink
                       ov
                       1- it
                         **aiint**
                         just
                         rap
                         ,
                         i
                         body
                         ladies
                         in
                         one
                         line
                         ,
                         talking
                         slick
                         to
                         three
                         chicks
                         at
                         one
                         time
                         .- @keem\_o\_therapy
                           it
                           was
                           a
                           nasty
                           job
                           that
                           i
                           **aiint**
                           feel
                           like
                           doin- i
                             hate
                             wen
                             niggas
                             try
                             to
                             go
                             n
                             on
                             otha
                             ppl
                             wen
                             dey
                             mugged
                             n
                             washed
                             like
                             go
                             siit
                             dwn
                             man
                             !
                             u
                             def
                             **aiint**
                             it
                             !- @qu33n\_sassy
                               datz
                               gud
                               real
                               gud
                               but
                               ii
                               **aiint**
                               goin
                               2
                               la
                               rekordiing- yhu
                                 **aiint**
                                 trippn
                                 butt
                                 steadii
                                 bringn
                                 it
                                 up
                                 #whytho- #smt
                                   da
                                   carebear
                                   moviie
                                   **aiint**
                                   free
                                   !
                                   nd
                                   ii
                                   wanna
                                   watch
                                   iit
                                   !- ii
                                     **aiint**
                                     kno
                                     dere
                                     wasz
                                     street
                                     kalled
                                     frost
                                     st
                                     lmao
                                     aww
                                     man- @savage\_lady22
                                       plz
                                       dnt
                                       ckuz
                                       iit
                                       **aiint**
                                       worth
                                       the
                                       headache- lol
                                         dat
                                         **aiint**
                                         miines
                                         soo
                                         get
                                         on
                                         sandra
                                         ..

## couldve

1. @tre\_bae\_bee
   defense
   ,
   he
   jus
   had
   greater
   offense
   .
   same
   thing
   with
   wade
   ,
   bosh
   had
   a
   quiet
   game
   ,
   did
   jus
   enough
   ,
   **couldve**
   did
   more- **couldve**
     been
     a
     loott
     better
     night
     ..
     but
     it
     also
     coulda
     been
     alot
     worse
     ..
     i
     really
     hope
     it's
     not
     over- lmaoo
       i
       **couldve**
       sworn
       niggas
       knew
       my
       @
       name
       ..
       "
       people
       "
       lol
       need
       to
       use
       it
       next
       time
       !- jus
         found
         my
         licensce
         tht
         i
         **couldve**
         sworn
         the
         cop
         tht
         tried
         2
         but
         #failed
         2
         get
         me
         4
         a
         duiy
         never
         gave
         back
         ..
         glad
         i
         had
         a
         back
         up
         anyways- man
           this
           loud
           ass
           truck
           outside
           cuttin
           dwn
           sum
           trees
           woke
           me
           up
           ugh
           dammit
           this
           so
           rude
           **couldve**
           waited
           til
           like
           11- you'll
             realize
             you
             **couldve**
             had
             something
             great
             #stupidhead- **couldve**
               went
               to
               hollywood
               tonight
               but
               nope
               it's
               cool
               i'd
               rather
               spend
               a
               night
               with
               some
               of
               my
               best
               friends- @bigolbitees
                 don't
                 do
                 that
                 u
                 had
                 my
                 number
                 you
                 **couldve**
                 called
                 n
                 reminded
                 me- @tymylaces
                   i
                   **couldve**
                   went
                   in
                   ,
                   but
                   i
                   didnt
                   ..- nets
                     **couldve**
                     made
                     smarter
                     decisions
                     down
                     the
                     stretch- @rickyruckus
                       damn
                       ,
                       lol
                       !!
                       that's
                       crazy
                       ,
                       i
                       wish
                       you
                       **couldve**
                       got
                       that
                       twitpic
                       though
                       ,
                       only
                       in
                       the
                       damn
                       tre'!!- @bleu\_billions
                         i
                         **couldve**
                         paid
                         some
                         of
                         my
                         dam
                         bills
                         wit
                         that
                         .
                         omg
                         ya
                         tweets
                         gettin
                         me
                         mad- @joka118
                           uu
                           \*
                           banged
                           it
                           on
                           me
                           ..
                           smh
                           something
                           **couldve**
                           happened
                           to
                           me
                           out
                           there
                           yo- @mad\_arie\_1dub
                             u
                             **couldve**
                             fooled
                             me
                             but
                             how
                             bout
                             we
                             talk
                             about
                             this
                             off
                             the
                             air- if
                               yu
                               vaccumed
                               when
                               i
                               told
                               yu
                               to
                               we
                               **couldve**
                               been
                               gone
                               #stupid
                               #managerondutytypeshit- westbrook
                                 mann
                                 ..
                                 u
                                 **couldve**
                                 gotten
                                 a
                                 better
                                 shot
                                 than
                                 that
                                 for
                                 the
                                 2
                                 for
                                 1
                                 ..
                                 smh- just
                                   saw
                                   a
                                   tweet
                                   on
                                   my
                                   tl
                                   that
                                   i
                                   wish
                                   i
                                   **couldve**
                                   responded
                                   too
                                   .
                                   lawwd
                                   .
                                   lol- @hemmm
                                     lol
                                     pokemon
                                     is
                                     sick
                                     i
                                     grew
                                     up
                                     with
                                     it
                                     .
                                     i
                                     **couldve**
                                     been
                                     in
                                     it
                                     but
                                     they
                                     said
                                     i
                                     was
                                     "
                                     too
                                     advanced
                                     and
                                     had
                                     an
                                     enormous
                                     advantage
                                     .
                                     "
                                     lol
                                     jk- @goodkharma\_
                                       i
                                       think
                                       dr.
                                       rice
                                       aint
                                       never
                                       gonna
                                       break
                                       out
                                       of
                                       this
                                       role
                                       ..
                                       she
                                       **couldve**
                                       given
                                       that
                                       sista
                                       a
                                       thank
                                       you
                                       girl
                                       or
                                       something
                                       ..
                                       lol- @stephaniebenoit
                                         i
                                         **couldve**
                                         had
                                         said
                                         it
                                         better
                                         myself
                                         !!

## wulda

1. @inkedup\_berry
   if
   u
   **wulda**
   said
   hi
   lik
   a
   normal
   person
   instead
   of
   calln
   me
   a
   punk
   ass
   mayb
   i
   **wulda**
   lol- i
     wish
     i
     **wulda**
     had
     off
     2day
     !
     i
     **wulda**
     woke
     up
     nd
     saw
     how
     hot
     it
     is
     nd
     i
     swear
     i
     **wulda**
     went
     to
     da
     beach
     by
     myself
     !
     i
     just
     want
     a
     tan- @\_chichi
       yeaa
       i
       got
       my
       first
       one
       at
       14
       &
       it
       all
       started
       there
       !
       wish
       i
       **wulda**
       waited
       tho
       i'm
       runnin
       outta
       space
       lol- did
         not
         kno
         the
         fuccin
         bank
         was
         dis
         far
         ii
         **wulda**
         said
         fuccit- sum
           dude
           saw
           dat
           i
           was
           wearn
           a
           mu
           shirt
           n
           said
           he
           nvr
           **wulda**
           saw
           some1
           dat
           went
           der
           ..
           on
           top
           of
           dat
           he
           was
           gettn
           on
           da
           trolly
           and
           kinda
           scruf- @badass\_chrissy
             lemme
             kno
             how
             tht
             go
             kuz
             my
             shit
             **wulda**
             ben
             blown
             2
             tha
             max
             too
             .!- practice
               **wulda**
               been
               so
               much
               more
               fun
               if
               i
               wasn't
               about
               to
               pass
               out- @xosunshineee
                 @ronib00
                 dem
                 niggaz
                 is
                 soo
                 lucky
                 @eluv203
                 wasn't
                 there
                 .
                 god
                 bless
                 dem
                 ,
                 cuz
                 that
                 club
                 **wulda**
                 simply
                 got
                 let
                 out
                 early
                 lmao- @lovealexnoelle
                   lmao
                   yo
                   scaryy
                   self
                   .
                   i
                   **wulda**
                   been
                   tryna
                   fight
                   da
                   door
                   bell
                   .
                   :-)- @jiffybodydrop
                     o0o
                     ok
                     ok
                     ..
                     she
                     prolly
                     still
                     inna
                     labor
                     cause
                     ifn
                     e
                     ting
                     she
                     **wulda**
                     get
                     a
                     room
                     an
                     u
                     **wulda**
                     hear
                     sutn- i
                       still
                       **wulda**
                       picked
                       john
                       tho- aww
                         i
                         jus
                         took
                         my
                         lil
                         cousin
                         pooh
                         to
                         buy
                         her
                         first
                         eye
                         shadow
                         and
                         mascara
                         set
                         !
                         who
                         **wulda**
                         thought
                         i
                         wuld
                         be
                         teachin
                         anyone
                         bout
                         makeup
                         ?!?- i
                           **wulda**
                           caught
                           ha
                           ass
                           slippin
                           !!- @brooklynszbunny
                             lol
                             a
                             simple
                             mwen
                             renmen'w
                             **wulda**
                             been
                             better- @do\_it\_truitt
                               bra
                               fuck
                               i
                               **wulda**
                               loved
                               to
                               hav
                               gone
                               to
                               dt
                               shit- @acewuzhere
                                 but
                                 1
                                 time
                                 it
                                 was
                                 20
                                 g1s
                                 and
                                 20
                                 blackberry
                                 curves
                                 ..
                                 if
                                 i
                                 was
                                 a
                                 thief
                                 i
                                 **wulda**
                                 took
                                 it
                                 ..
                                 but
                                 i'm
                                 not
                                 lol- @aleseeyaa
                                   dang
                                   it
                                   u
                                   shulda
                                   told
                                   me
                                   u
                                   was
                                   going
                                   i
                                   **wulda**
                                   went
                                   to- @ls\_made
                                     awl
                                     .
                                     wish
                                     yu
                                     **wulda**
                                     stayed
                                     at
                                     the
                                     g
                                     ,
                                     we
                                     needed
                                     a
                                     good
                                     soccer
                                     player
                                     lol- @foreveryung15
                                       lol
                                       yea
                                       ..
                                       smh
                                       i
                                       wasn't
                                       mad
                                       tho
                                       we
                                       was
                                       still
                                       winnin
                                       ..
                                       if
                                       they
                                       did
                                       that
                                       n
                                       we
                                       was
                                       losin
                                       then
                                       i
                                       **wulda**
                                       got
                                       a
                                       tech
                                       lmao- @mc\_great
                                         sis
                                         we
                                         n
                                         new
                                         brunswick
                                         ..
                                         u
                                         kno
                                         if
                                         we
                                         was
                                         home
                                         i
                                         **wulda**
                                         told
                                         u
                                         :)

## shulda

1. ofwgkta
   **shulda**
   been
   nominated- yo
     my
     tt
     **shulda**
     been
     up
     on
     the
     bet- #ilikedyouuntil
       -after
       we
       fuck
       yu
       tell
       me
       yu
       qotta
       qirl
       but
       still
       expect
       for
       us
       to
       fuck
       no
       niqqah
       ..
       **shulda**
       been
       real
       frm
       da
       jump
       str- lik
         really
         tho
         .
         .
         bosh
         !
         ?
         **shulda**
         gave
         it
         to
         miller
         since
         he
         wanted
         to
         be
         clutch
         tonite
         !
         ha- @missiworah
           u
           **shulda**
           called
           me
           i
           was
           out
           all
           night
           til
           4am- i
             **shulda**
             cheated
             ..
             ii
             **shulda**
             let
             yu
             go
             ..
             ii
             jus
             shake
             em
             off
             etch
             a
             sketch
             a
             hoe
             ..- i
               deff
               **shulda**
               smoked
               before
               this
               ..- @deweygat
                 oh
                 lord
                 i
                 **shulda**
                 known
                 yu
                 was
                 gon
                 say
                 sumthnn
                 lmao- i
                   love
                   nate
                   and
                   all
                   ,
                   but
                   i
                   think
                   dude
                   **shulda**
                   won
                   !
                   feelin
                   like
                   itz
                   a
                   win
                   by
                   popularity- urghh
                     **shulda**
                     def
                     came
                     to
                     the
                     center
                     earlyy
                     this
                     morn
                     smh- **shulda**
                       jus
                       went
                       to
                       atl
                       this
                       weekend
                       !- @ntertainer\_icon
                         lol
                         it
                         was
                         coo
                         !!
                         dats
                         wat
                         u
                         **shulda**
                         b
                         doing
                         n
                         e
                         way
                         lol- @datgutta\_nigga
                           lml
                           i
                           didnt
                           noe
                           yu
                           **shulda**
                           txted
                           me
                           and
                           tol
                           me- "
                             @vikingslb54
                             :
                             @106andpark
                             @boomkack
                             y
                             did
                             that
                             girl
                             win
                             she
                             sucked
                             the
                             girl
                             with
                             dance
                             on
                             her
                             shirt
                             **shulda**
                             won
                             "
                             ikr
                             lol
                             she
                             was
                             killing
                             it- popz
                               juss
                               made
                               me
                               feel
                               like
                               shit
                               tlkn
                               abt
                               i
                               aint
                               improve
                               since
                               last
                               summer
                               ;
                               i
                               swear
                               iwish
                               i
                               never
                               start
                               playing
                               **shulda**
                               been
                               a
                               game
                               freak- this
                                 id
                                 says
                                 i'm
                                 20
                                 i
                                 **shulda**
                                 jus
                                 went
                                 the
                                 whole
                                 way
                                 wit
                                 21
                                 rite
                                 ?- @neecy0628
                                   u
                                   **shulda**
                                   asked
                                   for
                                   his
                                   number
                                   wit
                                   ur
                                   chicken
                                   ..
                                   say
                                   somethin
                                   slick
                                   to
                                   blow
                                   his
                                   mind
                                   .
                                   use
                                   ur
                                   charm
                                   girl
                                   !!
                                   lmao- @tasteme\_
                                     lol
                                     soo
                                     u
                                     **shulda**
                                     had
                                     bbm
                                     so
                                     i
                                     wudnt
                                     havta
                                     tlk
                                     to
                                     u
                                     on
                                     slow
                                     ass
                                     twitter- **shulda**
                                       never
                                       stopped
                                       playin
                                       football- @marissa\_luv
                                         lol
                                         u
                                         **shulda**
                                         came
                                         with
                                         me
                                         so
                                         i'm
                                         not
                                         that
                                         bored
                                         .

## wouldve

1. i
   surely
   **wouldve**
   been
   there- @oui\_pariee
     really
     ?
     i
     **wouldve**
     fucked
     with
     the
     guy
     from
     kill
     bill
     2- @sneaker\_nerd
       who
       **wouldve**
       thought
       5-6
       glasses
       of
       wine
       **wouldve**
       fucked
       me
       up
       like
       that
       ??
       i'm
       good
       now
       !
       lol- "
         @jahnellywellys
         :
         and
         i
         aint
         even
         tripping
         ,
         cause
         we
         **wouldve**
         never
         last
         .
         "
         yeah
         we
         have- @jaypee\_z
           **wouldve**
           slept
           with
           gloria
           james
           if
           meant
           lebron
           stayin
           in
           cleveland
           .
           #desperate- @whitneylkennedy
             lol
             i
             **wouldve**
             gone
             but
             it's
             to
             cold
             lol- coke
               zero
               is
               not
               as
               bad
               as
               i
               **wouldve**
               expected- @golde\_locs
                 ohh
                 !
                 thought
                 u
                 **wouldve**
                 been
                 sleep
                 !- @pdahlem
                   haha
                   .
                   we've
                   integrated
                   vimeo
                   and
                   flickr
                   onto
                   our
                   media
                   page
                   .
                   free
                   hosting
                   and
                   bandwidth
                   that
                   we
                   **wouldve**
                   had
                   to
                   pay
                   for
                   otherwise
                   .- they
                     **wouldve**
                     won
                     best
                     group
                     to
                     shake
                     your
                     ass
                     too
                     haha
                     #travisporter- i
                       stopped
                       after
                       margarita
                       #1
                       due
                       to
                       that
                       damn
                       earthquake
                       !
                       i
                       shouldve
                       kept
                       it
                       going
                       i
                       **wouldve**
                       been
                       so
                       on
                       right
                       now- they
                         shouldve
                         put
                         @justinbieber
                         to
                         sing
                         :)
                         it
                         **wouldve**
                         been
                         way
                         better- #whatif
                           i
                           didn't
                           know
                           how
                           to
                           throw
                           it
                           back
                           ..
                           **wouldve**
                           been
                           a
                           waste
                           of
                           this
                           ass
                           !!- @mzchelle2u
                             the
                             visual
                             just
                             made
                             me
                             lol
                             !!
                             ur
                             kids
                             **wouldve**
                             thought
                             u
                             did
                             magic
                             !!- @shakethatash\_
                               that
                               totally
                               makes
                               sense
                               ,
                               reason
                               i
                               ask
                               is
                               because
                               i
                               grew
                               up
                               in
                               a
                               2
                               parent
                               home
                               and
                               sometimes
                               i
                               thought
                               it
                               **wouldve**
                               been
                               betta- "
                                 @incomparable2me
                                 :
                                 @buckalmighty
                                 \*
                                 lol
                                 she
                                 went
                                 right
                                 ..
                                 u
                                 went
                                 wrong
                                 ..
                                 this
                                 **wouldve**
                                 been
                                 funnier
                                 if
                                 u
                                 said
                                 i
                                 went
                                 left
                                 "
                                 \*
                                 fr
                                 fr
                                 lol- he
                                   gave
                                   one
                                   of
                                   the
                                   best
                                   performances
                                   .
                                   i'm
                                   big
                                   on
                                   english
                                   and
                                   grammar
                                   my
                                   last
                                   tweet
                                   was
                                   incorrect
                                   and
                                   **wouldve**
                                   meant
                                   he's
                                   the
                                   actual
                                   performace- @empressmarleyy
                                     yeah
                                     was
                                     gonna
                                     beep
                                     the
                                     horn
                                     ..
                                     but
                                     that
                                     **wouldve**
                                     been
                                     weird
                                     .- @mrudeness
                                       t.i
                                       !
                                       lol
                                       ,
                                       if
                                       i
                                       hear
                                       that
                                       1
                                       moe
                                       time
                                       :)
                                       it
                                       was
                                       str8
                                       last
                                       nite
                                       tho
                                       ,
                                       **wouldve**
                                       been
                                       beta
                                       if
                                       muna
                                       was
                                       n
                                       da
                                       buildin- patriots
                                         finally
                                         got
                                         a
                                         break
                                         ,
                                         but
                                         if
                                         it
                                         was
                                         challenged
                                         they
                                         probably
                                         **wouldve**
                                         gave
                                         the
                                         ball
                                         to
                                         the
                                         ravens
                                         .

## shouldve

1. holy
   crap
   .
   my
   class
   doesn't
   start
   til
   2
   ..
   i
   **shouldve**
   gotten
   something
   to
   eat
   !- i
     knew
     i
     **shouldve**
     jus
     stayed
     sleep
     ..
     always
     some
     shit- i
       have
       to
       stay
       still
       until
       my
       toes
       dry
       !
       this
       is
       rough
       !
       i
       **shouldve**
       showered
       before
       i
       started
       this
       ..
       oh
       well
       !- @onlyprecious1
         damn
         .
         i
         **shouldve**
         known
         that- crap
           !
           the
           man
           who's
           always
           jabbing/crushing
           me
           in
           his
           sleep
           just
           sat
           nxt
           to
           me
           .
           **shouldve**
           kept
           my
           purse
           in
           the
           seat- we
             **shouldve**
             listened
             to
             woody
             harrelson
             !
             stay
             away
             from
             yellowstone
             !
             everybody
             get
             on
             the
             ship
             ,
             i
             got
             the
             map
             !- i
               **shouldve**
               never
               let
               myself
               go
               bak
               2
               that
               ..
               waste
               of
               time
               &
               $$- ppl
                 **shouldve**
                 already
                 know
                 this
                 nigga
                 osama
                 was
                 dead
                 he
                 was
                 planning
                 as
                 shit- u
                   went
                   2
                   tha
                   club
                   early
                   but
                   still
                   had
                   2
                   pay
                   $20
                   ,
                   #commonsense
                   tells
                   u
                   ,
                   u
                   **shouldve**
                   got
                   a
                   presale
                   ticcet- while
                     they
                     taking
                     pics
                     they
                     **shouldve**
                     just
                     pointed
                     to
                     da
                     left
                     n
                     ride
                     that
                     wave- da
                       lights
                       went
                       out
                       and
                       scared
                       me
                       ,
                       maybe
                       i
                       **shouldve**
                       had
                       em
                       turned
                       off
                       already
                       lol- @krazisexirude
                         \*
                         blushes
                         \*
                         you
                         **shouldve**
                         been
                         around
                         yesterday
                         i
                         made
                         a
                         strawberry
                         shortcake
                         ,
                         but
                         its
                         gone
                         already- @barrettsallee
                           yes
                           -
                           don't
                           wait
                           so
                           long
                           to
                           use
                           bacon
                           !
                           it
                           **shouldve**
                           been
                           included
                           in
                           every
                           dish
                           by
                           this
                           point
                           !- i
                             **shouldve**
                             took
                             a
                             nap- @iismokegood
                               ahahaha
                               ooh
                               woww
                               ,
                               that
                               sounds
                               pretty
                               good
                               !
                               lol
                               i
                               really
                               **shouldve**
                               spent
                               it
                               with
                               you
                               lol- i've
                                 **shouldve**
                                 eaten
                                 a
                                 second
                                 breakfast
                                 .- noo
                                   you
                                   **shouldve**
                                   just
                                   stayed
                                   @2stevo
                                   .
                                   nigga
                                   .- this
                                     political
                                     debate
                                     going
                                     on
                                     around
                                     me
                                     <<
                                     ..
                                     **shouldve**
                                     kept
                                     my
                                     ass
                                     in
                                     the
                                     bed- ron
                                       **shouldve**
                                       threw
                                       the
                                       hood
                                       up
                                       and
                                       cracked
                                       his
                                       ass- @tleewins
                                         it
                                         was
                                         soo
                                         fun
                                         ,
                                         u
                                         **shouldve**
                                         gone
                                         !

## wudda

1. @krystylbystyl
   lmaoo
   ur
   damn
   right
   thats
   what
   i
   **wudda**
   done
   ,
   but
   then
   rubb
   ya
   bottom
   so
   it
   feels
   better
   lol- @beenhadpolos
     lmfaoo
     my
     shidd
     will
     soon
     get
     up
     there
     ;
     eww
     hawtt
     breath
     i
     **wudda**
     cut
     her
     ass- son
       if
       i
       was
       home
       i
       **wudda**
       been
       runnin
       to
       that
       shower- mi
         love
         de
         way
         u
         look
         like
         how
         ,
         like
         say
         u
         **wudda**
         suck
         a
         cocky
         tight
         now- so
           that
           nigga
           **wudda**
           been
           a
           nigerian
           king
           ?!
           lmfao- @stephiie\_rocks
             twitcon
             says
             "
             o
             you
             tatted
             too
             ?..
             fuck
             you
             "
             ..
             lol/
             a
             middle
             finger
             **wudda**
             been
             great
             ..- @melaela
               it
               **wudda**
               been
               better
               if
               nuggets
               won
               .- **wudda**
                 upp
                 twitter
                 wat
                 it
                 look- @swaggeris
                   nigga
                   shut
                   up
                   !!
                   you
                   **wudda**
                   did
                   da
                   same
                   thing- watching
                     the
                     tip
                     drill
                     video
                     if
                     i
                     was
                     18
                     when
                     they
                     recorded
                     this
                     i
                     so
                     **wudda**
                     been
                     in
                     it- @mrsproperlady
                       maan
                       i
                       **wudda**
                       blacked
                       on
                       her
                       i'm
                       sorry- @reeseonyourlips
                         i
                         **wudda**
                         odeed
                         on
                         they
                         soul
                         lol
                         ,
                         but
                         if
                         u
                         **wudda**
                         spazzed
                         i
                         **wudda**
                         understood
                         lol
                         ,
                         i
                         think
                         i
                         told
                         u
                         i
                         love
                         you
                         haha
                         woow- @mycashhairndick
                           damn
                           ii
                           thought
                           we
                           go
                           wai
                           bak
                           ii
                           **wudda**
                           paid
                           4
                           yu
                           lol- my
                             mom
                             just
                             left
                             to
                             go
                             to
                             the
                             casino
                             ..
                             u
                             **wudda**
                             thought
                             she
                             wud
                             take
                             me
                             smh
                             ..
                             stuck
                             here- @creolove
                               lmao
                               we
                               cudda
                               sold
                               that
                               to
                               julio
                               or
                               jose
                               for
                               the
                               l
                               o
                               he
                               **wudda**
                               bought
                               it
                               lol- @vthadon
                                 too
                                 late
                                 !!
                                 \*
                                 gives
                                 u
                                 a
                                 half
                                 assed
                                 kick
                                 like
                                 the
                                 ravens
                                 kicker
                                 \*
                                 rofl
                                 !!
                                 i
                                 said
                                 pats
                                 **wudda**
                                 won
                                 by
                                 3
                                 points
                                 or
                                 overtime
                                 lol- @withluv\_nia
                                   andd
                                   u
                                   **wudda**
                                   got
                                   beat
                                   ..
                                   u
                                   know
                                   the
                                   deal
                                   and
                                   where
                                   u
                                   go
                                   ?- @trevparanoia
                                     i
                                     are
                                     dnt
                                     get
                                     it
                                     yet
                                     ..
                                     mi
                                     **wudda**
                                     beg
                                     stephen
                                     but
                                     him
                                     change
                                     him
                                     pin
                                     -\_\_-- lana
                                       a
                                       talk
                                       true
                                       she
                                       married
                                       lol
                                       mek
                                       she
                                       was
                                       just
                                       a
                                       baby
                                       mada
                                       and
                                       u
                                       **wudda**
                                       see- @iamdnayb
                                         :
                                         ok
                                         !!
                                         look
                                         at
                                         detective
                                         mocha
                                         over
                                         here
                                         lol
                                         !
                                         yea
                                         iwas
                                         tryna
                                         get
                                         in
                                         it
                                         to
                                         .
                                         it
                                         was
                                         iite
                                         ,
                                         but
                                         it
                                         **wudda**
                                         got
                                         better
                                         .
                                         that's
                                         sad
                                         tho
                                         .

## shudda

1. @murdamookez
   u
   **shudda**
   known
   i
   was
   gonna
   download
   soon
   as
   you
   dropped
   that
   shit
   ..
   my
   cologne
   only
   way
   u
   get
   a
   scent
   from
   me
   (
   smh
   )- @taytagotbody
     ssoo
     nigga
     ain't
     kno
     u
     still
     wanted
     to
     go
     u
     **shudda**
     hit
     me
     up- i
       am
       so
       mad
       i
       wasn't
       hungry
       !!
       they
       had
       a
       20-piece
       nugget
       for
       $5
       !!
       i
       **shudda**
       still
       got
       it
       ..- fuxn
         right
         robinson
         boy
         u
         **shudda**
         been
         hoppin
         up
         like
         dat
         !!- @jessicamarie022
           @djmoska
           it
           was
           horrible
           !
           i
           **shudda**
           stayed
           home
           n
           watched
           save
           by
           the
           bell
           cuz
           even
           screetch
           is
           more
           entertaining
           lol- @lookitslon
             bull-fuckin-shit
             !!
             you
             **shudda**
             told
             me
             that
             before
             he
             became
             who
             he
             is
             ctfu- good
               luck
               to
               my
               babe
               jordan
               in
               his
               football
               game
               go
               jr
               vikings
               against
               grant
               :)
               i
               **shudda**
               went
               insted
               of
               cryin
               bout
               my
               toe- damn
                 i
                 **shudda**
                 brought
                 my
                 keyboard
                 to
                 skool
                 ..
                 im
                 bored
                 af
                 right
                 now- lebron
                   **shudda**
                   got
                   his
                   team
                   involved
                   early
                   in
                   the
                   game- @ayoitsjflo
                     hahaha
                     ur
                     fam
                     **shudda**
                     know
                     better
                     :p
                     but
                     hahaha
                     that
                     works
                     ;
                     b
                     my
                     mom
                     wnt
                     be
                     mad
                     she'll
                     be
                     drunk- @mugsy\_718
                       aww
                       u
                       **shudda**
                       invited
                       me- @djyoung\_yg
                         lowkey
                         made
                         me
                         mad
                         ..
                         i
                         quote
                         "
                         you
                         **shudda**
                         passed
                         it
                         to
                         me
                         "
                         those
                         were
                         ur
                         words
                         an
                         u
                         were
                         standin
                         right
                         behind
                         me- i
                           be
                           so
                           madd
                           when
                           i
                           run
                           in
                           to
                           a
                           chick
                           i
                           **shudda**
                           hit
                           and
                           didnt
                           ..- @the\_org\_goon
                             i
                             **shudda**
                             punched
                             u
                             in
                             ya
                             fucking
                             jaw
                             wen
                             i
                             saw
                             u
                             .
                             suc
                             ma
                             dic
                             pussy- @ms\_hazel\_
                               told
                               u
                               u
                               **shudda**
                               came
                               over
                               smh
                               u
                               good
                               did
                               u
                               finish
                               ur
                               schoolwork
                               ?- i
                                 **shudda**
                                 blasted
                                 u
                                 -\_-
                                 @spoilmeonjuly13- **shudda**
                                   neva
                                   ate
                                   dem
                                   mc
                                   griddles
                                   dis
                                   morning
                                   smh
                                   dey
                                   deff
                                   fucked
                                   my
                                   stomach
                                   up
                                   smh- damn
                                     i
                                     was
                                     comfortable
                                     i
                                     **shudda**
                                     nvr
                                     got
                                     mah
                                     ass
                                     up- @pusha\_p\_
                                       i
                                       kno
                                       i
                                       **shudda**
                                       jus
                                       kept
                                       it
                                       to
                                       myself
                                       i
                                       thought
                                       about
                                       it
                                       for
                                       like
                                       5
                                       mins
                                       b4
                                       i
                                       posted
                                       it- i
                                         **shudda**
                                         brought
                                         my
                                         ipod
                                         smh

## wuda

1. @ubuy\_isell
   lmao
   i
   **wuda**
   bn
   cryin
   n
   dat
   bitch- for
     the
     record
     i
     **wuda**
     cleared
     the
     first
     one
     up
     i
     **wuda**
     nipped
     it
     in
     the
     bud
     but
     yu
     didn't
     ..
     how
     much
     yu
     want
     me
     deal
     wit- if
       i
       **wuda**
       known
       my
       teachers
       worked
       this
       hard
       i
       **wuda**
       been
       a
       dentist
       !
       grr
       tht
       #2
       on
       my
       list
       !- @justskell
         now
         unno
         i
         **wuda**
         if
         i
         kuda
         ,
         n
         plus
         i'm
         still
         tryna
         get
         ova
         our
         last
         encounter
         lls
         \*
         extends
         arm
         for
         hug- i
           **wuda**
           stuck
           him
           a
           hedbutt
           comeing
           to
           my
           face
           like
           he
           was
           to
           that
           reporter- @nbm\_lo
             my
             my
             xbox
             was
             still
             working
             i
             **wuda**
             buss
             dat
             ass
             in
             madden- awh
               her
               feelings
               is
               hurt
               ..
               mine
               **wuda**
               been
               2
               even
               tho
               she
               had
               the
               same
               concerns
               lol
               she
               feels
               dumpd
               n
               wantd
               2
               b
               tha
               dumper
               not
               tha
               dumpee- @heiress\_niecey
                 o
                 ..
                 lol
                 i
                 **wuda**
                 thought
                 u
                 knew- @retrospec\_ofccc
                   loll
                   if
                   u
                   **wuda**
                   been
                   there
                   sat
                   night
                   u
                   shud
                   have
                   understood
                   !- @l3ftyg3tsitdon3
                     u
                     can
                     read
                     ?
                     woww
                     who
                     **wuda**
                     thought
                     lmfao
                     #justkidding- victoria
                       beckham
                       has
                       acne
                       who
                       **wuda**
                       known- @prissypretty
                         na
                         i
                         wud
                         have
                         ..
                         this
                         just
                         twitter
                         ..
                         idgaf
                         !!
                         i
                         **wuda**
                         just
                         be
                         laughin
                         like
                         shit
                         !!- quiz
                           went
                           well
                           ..
                           so
                           hungry
                           what
                           shud
                           i
                           eat
                           ..
                           ahh
                           had
                           a
                           good
                           dream
                           lastnight
                           wit
                           a
                           fella
                           i
                           wish
                           won't
                           in
                           it
                           lol
                           ..
                           @willystylezz
                           **wuda**
                           ben
                           beta- @eloquentboi
                             haa
                             aww
                             thas
                             soo
                             cute
                             ,
                             =(
                             well
                             it
                             **wuda**
                             been
                             nice
                             to
                             see
                             u
                             tonite
                             too- got
                               enuff
                               juice
                               to
                               speak
                               i
                               **wuda**
                               been
                               sick- "
                                 i
                                 **wuda**
                                 came
                                 bac
                                 4
                                 u
                                 ..
                                 but
                                 i
                                 jus
                                 needed
                                 time
                                 ..
                                 2
                                 do
                                 wat
                                 i
                                 had
                                 2
                                 do
                                 "- @duuuhnae
                                   sorry
                                   nae
                                   we
                                   **wuda**
                                   came
                                   to
                                   get
                                   u
                                   bc
                                   the
                                   bros
                                   backed
                                   out- wat
                                     kittie
                                     **wuda**
                                     done
                                     if
                                     he
                                     sed
                                     no
                                     lmaoo- @t\_kristen
                                       i
                                       thought
                                       u
                                       **wuda**
                                       saw
                                       it
                                       by
                                       now
                                       but
                                       aye
                                       ull
                                       get
                                       over
                                       it- @fancy\_bre
                                         aww
                                         ,,
                                         u
                                         shuda
                                         @
                                         me
                                         then
                                         nd
                                         wheneva
                                         i
                                         kame
                                         on
                                         i
                                         **wuda**
                                         seen
                                         nd
                                         u
                                         **wuda**
                                         been
                                         the
                                         1st
                                         person
                                         i
                                         wrote
                                         to
                                         lol

## shuda

1. @dayoungone
   u
   **shuda**
   brought
   a
   snack
   to
   keep
   u
   up
   or
   sumthin
   ..- #letsbereal
     i
     **shuda**
     known
     **shuda**
     knwn
     **shuda**
     knwn
     **shuda**
     knwn
     **shuda**
     known
     **shuda**
     knwn
     dat
     she
     was
     crazy
     den
     a
     muthafuka
     \*
     in
     my
     @bigsean
     voice
     \*- @6babyk9
       look
       on
       fb
       we
       battled
       it
       out
       ,
       but
       im
       tryna
       be
       grown
       and
       move
       on
       .
       its
       up
       to
       you
       if
       you
       still
       wana
       hold
       a
       grudge
       .
       you
       **shuda**
       told
       me
       .- @clevenole
         i
         don't
         know
         .
         you
         seem
         to
         over
         defend
         ur
         tm
         .
         the
         way
         u
         backed
         browns
         this
         year
         was
         eye
         opening
         .
         i
         think
         they
         **shuda**
         been
         called
         out- i
           **shuda**
           jus
           stayed
           n
           gboro
           4
           dis
           shit
           !- @carterkiddd
             lol
             fucking
             jock
             .
             to
             bad
             u
             **shuda**
             did
             it
             already
             cause
             today
             my
             last
             .- @mkemescrmzaddie
               yeoo
               we
               still
               ova
               hea
               ,
               u
               **shuda**
               came
               mann- yayy
                 i
                 got
                 my
                 jacket
                 but
                 its
                 too
                 big
                 ..
                 :(
                 i
                 **shuda**
                 got
                 a
                 size
                 .
                 8- nigga
                   you
                   **shuda**
                   been
                   had
                   ya
                   shit
                   together
                   like
                   yesterday
                   .- @popupblocker
                     lmfaoo
                     u
                     **shuda**
                     #
                     tht
                     and
                     retweet
                     it
                     haha
                     so
                     i
                     can
                     get
                     some
                     more
                     followers
                     lmfaoo
                     #twitafterdark
                     jk
                     lmaoo- oomf
                       **shuda**
                       neva
                       left
                       :(
                       imy- @gorgeousderisha
                         lol
                         man
                         i
                         **shuda**
                         broughta
                         driva
                         doe- @mszyaniboobabyy
                           i
                           got
                           u
                           lol
                           i
                           **shuda**
                           been
                           following
                           u
                           ..
                           i
                           been
                           lookin
                           for
                           u- so
                             much
                             ice
                             ,
                             i
                             **shuda**
                             brought
                             my
                             furr
                             brr- thats
                               fucked
                               up
                               .
                               you
                               have
                               to
                               give
                               head
                               to
                               help
                               save
                               your
                               own
                               life
                               now
                               .
                               so
                               if
                               u
                               get
                               breast
                               cancer
                               u
                               think
                               "
                               shit
                               **shuda**
                               sucked
                               some
                               penis
                               "
                               ..- @lexphilex06
                                 **shuda**
                                 neva
                                 gave
                                 them
                                 niggas
                                 alpha- i
                                   swear
                                   my
                                   mommy
                                   **shuda**
                                   been
                                   a
                                   mind
                                   reader
                                   ..
                                   damn
                                   i
                                   can't
                                   hide
                                   anything
                                   4rm
                                   her
                                   ,
                                   she
                                   know
                                   exactly
                                   when
                                   sumthn
                                   wrong
                                   now
                                   i
                                   gota
                                   spill
                                   da
                                   beans- @yeeaaa\_sm00ve
                                     **shuda**
                                     came
                                     to
                                     doub
                                     house- 73
                                       people
                                       called
                                       out
                                       from
                                       my
                                       store
                                       today
                                       i
                                       **shuda**
                                       made
                                       it
                                       74
                                       !- @tiombe\_talkshit
                                         realshit
                                         i
                                         didn't
                                         .
                                         i
                                         **shuda**
                                         tho
                                         ,
                                         or
                                         you
                                         **shuda**
                                         so
                                         you
                                         wrong
                                         too

## mustve

1. @greggutfeld
   you
   **mustve**
   flown
   useless
   airways- @wtfis\_upman
     oh
     so
     it
     **mustve**
     been
     jay
     z
     lol
     ,
     yea
     i
     never
     heard
     that
     one
     lol- @diamond196
       just
       a
       lucky
       guess
       !
       i
       was
       there
       ,
       u
       **mustve**
       been
       too
       drunk
       to
       remember
       !- pete
         carroll
         **mustve**
         envisioned
         this
         when
         he
         signed
         .
         escape
         sanctions
         ,
         get
         big
         $
         ,
         and
         a
         chance
         to
         make
         the
         playoffs
         in
         a
         sorry
         ass
         division
         .- you
           **mustve**
           done
           this
           before
           !- @mr\_3live
             yeah
             that
             lil
             web
             site
             thing
             lol
             haha
             something
             **mustve**
             went
             wrong
             with
             the
             genes
             to
             make
             kevin
             so
             short
             lol- @mr\_ajones
               haa
               !
               i
               can
               work
               out
               bruhh
               !
               you
               **mustve**
               got
               me
               mixed
               up
               !!- @kmanchester
                 i'm
                 sorry
                 ,
                 did
                 you
                 stab
                 yourself
                 with
                 your
                 pink
                 ribbon
                 this
                 morning
                 ?
                 i'm
                 not
                 dropping
                 any
                 insults
                 here
                 .
                 i
                 **mustve**
                 struck
                 a
                 chord- so
                   my
                   aunt
                   goes
                   "
                   i
                   saw
                   the
                   nicest
                   ferrari
                   on
                   obt
                   yesterday
                   ,
                   and
                   the
                   guy
                   inside
                   was
                   black
                   w
                   gold
                   teeth
                   "
                   **mustve**
                   been
                   a
                   drugdealer
                   #fail- suffolk
                     county
                     policegivn
                     away
                     free
                     hamsdand
                     turkey
                     in
                     brentwood
                     they
                     **mustve**
                     got
                     a
                     lot
                     of
                     dudes
                     for
                     they
                     packages
                     lol
                     #doing
                     good- @robynnista
                       u
                       **mustve**
                       slit
                       ur
                       wrists
                       after
                       u
                       typed
                       that
                       bc
                       u
                       wldnt
                       b
                       able
                       2
                       if
                       u
                       did
                       it
                       b4- 103
                         unread
                         emails
                         ,
                         the
                         vip
                         **mustve**
                         been
                         crackin
                         while
                         i
                         was
                         in
                         the
                         air- my
                           fault
                           whiz
                           was
                           n
                           da
                           cypher
                           i
                           **mustve**
                           missed
                           it
                           !- @tast3ofh0n3y
                             ur
                             belly
                             **mustve**
                             been
                             feelin
                             szwell
                             !!
                             i'm
                             jealous
                             lol- chris
                               gone
                               take
                               my
                               charger
                               out
                               &
                               ndd
                               then
                               say
                               ,
                               "
                               you
                               still
                               my
                               nigga
                               "
                               lol
                               you
                               **mustve**
                               almost
                               got
                               slapped- @nicktittone
                                 i've
                                 seen
                                 a
                                 better
                                 celebration
                                 at
                                 my
                                 middle
                                 school
                                 graduation
                                 #remember1985
                                 **mustve**
                                 been
                                 nice
                                 to
                                 be
                                 alive
                                 for
                                 that
                                 huh- alotta
                                   people
                                   say
                                   money
                                   aint
                                   everything
                                   i
                                   say
                                   i
                                   **mustve**
                                   came
                                   from
                                   different
                                   planet
                                   cause
                                   that
                                   my
                                   everyday
                                   chase
                                   !- @im\_so\_badd
                                     noo
                                     why
                                     would
                                     i
                                     ?
                                     **mustve**
                                     been
                                     when
                                     i
                                     updated
                                     my
                                     bbm
                                     a
                                     few
                                     people
                                     got
                                     deleted
                                     .
                                     add
                                     me
                                     2103b4df- @snooki
                                       hahaha
                                       !
                                       it
                                       took
                                       both
                                       of
                                       you
                                       to
                                       kill
                                       it
                                       ??
                                       it
                                       **mustve**
                                       been
                                       massive
                                       !!
                                       lol- @ricademus
                                         i
                                         didn't
                                         do
                                         anything
                                         you
                                         **mustve**
                                         drank
                                         tea
                                         !

## nevr

1. wen
   i
   wanna
   tlk
   u
   **nevr**
   around
   or
   b
   on
   sum
   funny
   shit- @rezilience
     lol
     oh
     ok
     .
     i
     **nevr**
     did
     it
     b4- **nevr**
       under
       estimate
       someone
       you
       never
       know
       their
       .
       true
       ablilities- roaming
         through
         the
         cupboard
         jar
         of
         pickles
         **nevr**
         opened
         since
         1983
         ,
         peanuts
         in
         a
         pile&elvis
         dn
         the
         aisle
         singing
         gallantly-
         "
         vernie
         "
         blindmelon- i
           dnt
           need
           no
           nigga
           ,
           **nevr**
           did
           .
           and
           i
           know
           now
           nvr
           give
           a
           men
           my
           all
           unless
           he
           deserves
           it
           .- my
             kids
             get
             the
             worlds
             best
             images
             taken
             of
             them
             .
             when
             they
             move
             out
             they'll
             have
             2
             live
             w/
             the
             fact
             that
             they'll
             **nevr**
             look
             as
             gd
             .- titties
               r
               **nevr**
               rlly
               a
               make-or-break
               thing
               4
               me
               .
               i
               **nevr**
               had
               sex
               wit
               a
               chick
               &
               said
               "
               oh
               ur
               titties
               r
               ugly
               ,
               i
               gotta
               go
               "
               .- @get2knowhero
                 i've
                 **nevr**
                 sd
                 tht- it
                   takes
                   alot2b
                   a
                   dad
                   ;
                   it
                   takes
                   even
                   more2b
                   a
                   dad2
                   a
                   teen
                   uve
                   **nevr**
                   met
                   b4
                   thank
                   u
                   2my
                   foster
                   dad
                   &
                   happy
                   father's
                   day2all
                   !
                   #mmh
                   @jimmywayne- @quotablebuffy
                     so
                     one
                     by
                     one
                     they
                     turn
                     from
                     me
                     ,
                     i
                     guess
                     my
                     friends
                     can't
                     face
                     the
                     cold
                     ,
                     but
                     y
                     i
                     froze
                     not
                     one
                     among
                     knows
                     ,
                     and
                     **nevr**
                     can
                     b
                     told- i
                       used
                       2
                       say
                       **nevr**
                       never
                       .
                       wld
                       luv
                       lk
                       ths
                       **nevr**
                       thought
                       in
                       a
                       million
                       years
                       wld
                       b
                       hre
                       .
                       **nevr**
                       wld
                       a
                       player
                       hang
                       his
                       jersey
                       up
                       &
                       leave
                       the
                       game- @lovelye157
                         iim
                         on
                         fiiree
                         !!
                         how
                         have
                         u
                         **nevr**
                         seen
                         talladega
                         nights- lmao
                           i
                           **nevr**
                           said
                           i
                           couldnt
                           come
                           @aciesupreme
                           .
                           but
                           were
                           in
                           mahatten
                           we
                           tlkin
                           ?- this
                             shit
                             **nevr**
                             ends
                             man
                             .- @ogochocinco
                               who-dey
                               !..
                               go
                               bengals
                               !
                               **nevr**
                               liked
                               football
                               ,
                               til
                               ths
                               season
                               .
                               always
                               rooted
                               ,
                               **nevr**
                               watched
                               .
                               do
                               now
                               .
                               tryin
                               2
                               get
                               in2
                               it
                               .
                               rules
                               ?- #betmessedupwhen
                                 they
                                 chose
                                 rocsis
                                 '
                                 stylist
                                 ..
                                 girl
                                 **nevr**
                                 can
                                 put
                                 her
                                 clothing
                                 togethr
                                 right- @prettynpink01
                                   nothin
                                   is
                                   **nevr**
                                   as
                                   bad
                                   as
                                   it
                                   seems
                                   when
                                   u
                                   evaluate
                                   the
                                   situation
                                   &
                                   go
                                   ovr
                                   ur
                                   options
                                   everything
                                   comes
                                   2
                                   light
                                   hope
                                   all
                                   is
                                   well- **nevr**
                                     new
                                     a
                                     love
                                     like
                                     this
                                     supaa
                                     happy
                                     hahhahaha
                                     tellem
                                     y
                                     u
                                     mad
                                     ..
                                     stupid
                                     ass- ok
                                       im
                                       unfollowin
                                       mad
                                       heads
                                       right
                                       now
                                       .
                                       ppl
                                       dat
                                       **nevr**
                                       get
                                       at
                                       me
                                       so
                                       if
                                       u
                                       still
                                       anna
                                       be
                                       followed
                                       just
                                       say
                                       hi
                                       =)- i
                                         swear
                                         celebrities
                                         days
                                         r
                                         **nevr**
                                         overr
                                         nigga
                                         bout
                                         to
                                         party
                                         at
                                         4am
                                         n
                                         here
                                         n
                                         brooklyn
                                         dat
                                         shyt
                                         is
                                         over

## neva

1. **neva**
   worry
   about
   other
   ppl's
   opinions
   ..
   cause
   most
   of
   the
   time
   its
   from
   hate
   or
   lack
   of
   knowledge
   ..
   #studzlesson- man
     these
     turkeys
     just
     crossed
     the
     street
     an
     mugged
     me
     like
     i
     was
     in
     the
     wrong
     ..
     keep
     on
     ..
     **neva**
     had
     wild
     turkey
     salad
     befo
     !- i
       aint
       **neva**
       put
       it
       down
       like
       this
       boyy
       !!- @sundaedip4ya
         you
         **neva**
         hit
         me
         up
         !!- tryna
           b
           nice
           for
           a
           change
           !!..
           but
           **neva**
           again
           will
           i
           b
           nice
           to
           danny
           ray
           fountain
           ass
           !!- **neva**
             let
             nobody
             break
             yu
             &
             da
             person
             yu
             love
             apart
             !.,
             #fact- @lolliekinns
               i
               miss
               you
               too
               ,
               lol
               i
               sent
               u
               a
               message
               but
               u
               **neva**
               repied- @cupcake\_diva91
                 **neva**
                 say
                 **neva**- @boss\_motivation
                   **neva**
                   i
                   be
                   keepin
                   my
                   eyes
                   open
                   ..- @cyoshimi
                     since
                     when
                     does
                     your
                     ass
                     stay
                     in
                     whackass
                     sacramento
                     ?
                     i
                     coulda
                     then
                     burnt
                     a
                     blunt
                     w/
                     you
                     .
                     but
                     you
                     **neva**
                     hitt
                     me
                     w/
                     that
                     addy
                     .
                     haha
                     .- he
                       can
                       kick
                       it
                       to
                       yu
                       hoes
                       lik
                       he
                       dont
                       love
                       me
                       but
                       u
                       n
                       i
                       both
                       kno
                       he
                       love
                       me
                       but
                       wen
                       im
                       gone
                       he
                       gone
                       wisg
                       he
                       **neva**
                       thugged
                       me
                       #truetweet- @geebayme
                         man
                         fuck
                         you
                         #b4l
                         ain't
                         **neva**
                         stupid
                         yall
                         may
                         think
                         we
                         stupid
                         but
                         we
                         always
                         in
                         control- @chan3lissweet
                           **neva**
                           dat
                           like
                           em
                           since
                           mchale
                           ,
                           bird
                           ,
                           parish
                           ,
                           walton
                           etc
                           etc
                           da
                           ugliest
                           team
                           eva
                           but
                           has
                           mo
                           banners
                           n
                           rings
                           then
                           da
                           fakers
                           lol- @sparkdabeast
                             **neva**
                             said
                             it
                             was
                             still
                             down
                             and
                             im
                             chillin
                             wats
                             good
                             witcha- @the\_real\_dame
                               u
                               aint
                               **neva**
                               lied
                               cuz
                               i'm
                               gonna
                               catch
                               a
                               case
                               if
                               a
                               nicca
                               piss
                               on
                               me
                               i'm
                               so
                               sincere
                               ..- &
                                 i
                                 wish
                                 we
                                 **neva**
                                 loved
                                 it
                                 ..- valuable
                                   information
                                   :
                                   **neva**
                                   get
                                   in
                                   tha
                                   shower
                                   and
                                   then
                                   get
                                   dressed
                                   in
                                   a
                                   room
                                   with
                                   a
                                   air
                                   conditioner- omfg
                                     ,
                                     i
                                     cant
                                     **neva**
                                     like
                                     somebody
                                     more
                                     than
                                     aa
                                     couple
                                     of
                                     months
                                     i
                                     loose
                                     interst
                                     ughh
                                     !
                                     i
                                     hate
                                     this
                                     shxt- i
                                       sent
                                       it
                                       ..
                                       its
                                       krazii
                                       tho
                                       smh
                                       @msreddbon3
                                       wana
                                       tlk
                                       slackness
                                       but
                                       **neva**
                                       business- don't
                                         stress
                                         '
                                         em
                                         ;
                                         next
                                         '
                                         em
                                         &&'
                                         d
                                         pretend
                                         u
                                         **neva**
                                         met
                                         '
                                         em

## eva

1. @co\_yola\_abm
   lol
   well
   anyway
   i
   think
   yur
   kuzzin
   cheatin
   on
   me
   ..
   soo
   is
   he
   ??
   lls
   dont
   lie
   **eva**- @thepretty\_one
     u
     beta
     not
     be
     ;;
     lol
     iifu
     **eva**
     r
     jus
     let
     me
     no
     iiqhtt- if
       u
       **eva**
       wonder
       if
       im
       a
       freak
       or
       not
       stop
       wonder
       babe
       cus
       i
       am
       :)- bout
         to
         eat
         a
         burrito
         from
         chipotle
         !
         1st
         time
         **eva**
         gettin
         a
         burrito- fi
           a
           fluffy
           girl
           stacious
           stiff
           as
           **eva**- my
             sis
             jus
             told
             me
             "
             bari
             you've
             come
             way
             2
             far
             2
             **eva**
             look
             baq
             "- who
               **eva**
               unfollow
               me
               could
               eat
               a
               dick- @model\_ashh
                 when
                 do
                 we
                 **eva**
                 chill
                 thou
                 ash
                 .
                 umm
                 only
                 thanksgivin
                 n
                 fourth
                 of
                 july- @shabazzsd
                   lmfao
                   its
                   real
                   sad
                   she
                   been
                   her
                   since
                   8
                   and
                   been
                   sleep
                   **eva**
                   since
                   wat
                   13
                   year
                   old
                   sleeps
                   that
                   long- longest
                     nap
                     **eva**
                     !!- i
                       neva
                       seen
                       da
                       being
                       of
                       crooklyn
                       ..
                       **eva**
                       !
                       dat
                       shit
                       is
                       funny
                       i
                       needa
                       follow
                       @spikelee
                       .- lmaort
                         @grandthefrondo
                         :
                         he
                         asked
                         me
                         have
                         i
                         **eva**
                         had
                         sex
                         wit
                         a
                         white
                         man
                         ..
                         i
                         was
                         thinking
                         bout
                         asking
                         him
                         the
                         same
                         thing
                         #penitentiarynicca- breakfast
                           at
                           my
                           favorite
                           diner
                           ,
                           strawberry
                           picking
                           with
                           my
                           nieces
                           ,
                           a
                           wade
                           in
                           lake
                           minnewaska
                           ,
                           soft
                           shell
                           crabs
                           and
                           a
                           tan
                           .
                           best
                           bday
                           **eva**
                           .- #nowplaying
                             lil
                             kim
                             hardcore
                             album
                             baddest
                             shit
                             she
                             **eva**
                             did
                             #imjustsaying- @whoanellyx3
                               thas
                               fine
                               2day
                               isn't
                               tha
                               party
                               2mro
                               is
                               relle
                               buh
                               wen
                               **eva**
                               yu
                               ready- @evamula
                                 nah
                                 **eva**
                                 u
                                 go
                                 to
                                 bed
                                 ..
                                 u
                                 actually
                                 got
                                 skool
                                 tomoro
                                 ..
                                 let
                                 us
                                 grown
                                 folks
                                 tweet
                                 lol- @pdotmmr
                                   you
                                   my
                                   nigga
                                   i'm
                                   sorry
                                   for
                                   ya
                                   loss
                                   he
                                   was
                                   my
                                   nigg
                                   to
                                   jus
                                   hold
                                   ya
                                   head
                                   and
                                   everythin
                                   will
                                   be
                                   ok
                                   and
                                   who
                                   **eva**
                                   did
                                   this
                                   will
                                   get
                                   theirs- yu
                                     **eva**
                                     had
                                     a
                                     female
                                     right
                                     where
                                     yu
                                     want
                                     her
                                     .- best
                                       game
                                       yet
                                       .!
                                       n
                                       not
                                       bcus
                                       of
                                       d
                                       fightn
                                       ish
                                       bt
                                       because
                                       dats
                                       d
                                       hardest
                                       i
                                       have
                                       **eva**
[truncated: 39,573,562 more chars]
