# Supplementary material for: Diffusion of Lexical Change in Social Media
Source: PLoS One. 2014 Nov 19;9(11):e113114. doi: 10.1371/journal.pone.0113114 (PMC4237389; doi:10.1371/journal.pone.0113114)
Supplement: File S1 — Appendix S1-S3, Table S1 and Software S1. Appendix S1. Term list. List of all words considered in our main analysis. Appendix S2. Term examples. Examples for each term considered in our analysis. Appendix S3. Data Procedures. Description of the procedures used for data processing, including Twitter data acquisition, geocoding, content filtering, word filtering, and text processing. Table S1. Term annotations. Tab-separated file describing annotations of each term as entities, foreign-language, or acceptable for analysis. Software S1. Preprocessing software. Source code for data preprocessing. (ZIP) [file pone.0113114.s001.zip › supp_for_upload/Appendix_S3_Data_Procedures.pdf]

# S1: Data Procedures for "Diffusion of Lexical Change in Social Media"

We perform several preprocessing steps to prepare the raw Twitter feed for analysis, described in this document: (1) a message preprocessing pipeline, and (2) a selection procedure for the words to analyze.

# Messages

Our initial dataset is of Twitter Gardenhose/Decahose messages from August 2009 through September 2012, containing approximately 17 billion tweets. 721 million were found to have a geotag, and 171 million were located in the United States. After MSA and content filtering, 107 million messages (from 2.7 million unique user accounts) remained for the analysis. The preprocessing software is included as supplementary information file *preprocessing_software.zip*.

**Geotags** The Twitter API's structured data includes a field for latitude and longitude coordinates from users who have enabled geo-location; typically, these come from messages authored on mobile phones. Besides that field, there are also informal geotags in the *user.location* field, from clients that insert coordinates as a string; for example, *ÜT: 40.043883,-88.275849* is a geotag from the ÜberTwitter client. These informal geotags are more common in earlier data, and are the only source of coordinates before Twitter added official support for coordinate geotags in late 2009. A regular expression extracts this type of coordinates; there were about twice as many messages with informal coordinates as messages with official API coordinates. We use both types of messages.

**Location** We use only messages from the continental USA, locating the latitude and longitude coordinates to a county or county-equivalent, according to the U.S. Census Bureau's 2010 TIGER/Line Shapefiles. (`http://www.census.gov/geo/maps-data/data/tiger-line.html`). The United States Office of Management and Budget defines a set of Metropolitan Statistical Areas (MSAs), which are not legal administrative divisions, but rather, geographical regions centered around a single urban core [1]; every MSA is defined as a set of counties. We consider the 200 most populous MSAs in the lower 48 U.S. states. The most populous MSA is centered on New York City (population 19 million); the 200th most populous is Fargo, North Dakota (population 200,000). We retain messages whose location belongs to one of these MSAs. According to the 2010 census, the 200 largest MSAs include 76% of all US residents in the lower 48 states; however, we find that these MSAs contain 89% of all Twitter messages sent from within the lower 48 states, which coheres with recent work showing that geotagged Tweets are more likely to come from urban areas [2].

For each MSA, demographic attributes are computed from the 2010 U.S. Census. The following demographic attributes are included: log population, log median income, % residents in urbanized areas, media age, % renters, % African American; % Hispanic. We did not consider % European American because it has a strong negative correlation with % African American, $r = -0.71$; we did not consider % Asian American because it is much smaller, with a median value of 2.8%. Mean and standard deviations of all demographic attributes are shown in Table 2 of the main text.

**Content and Follower Filtering** Several additional processing steps were then performed to remove marketing-oriented and spam accounts. We remove all messages written by users who have more than 1000 followers, or who follow more than 1000 people. This helps to eliminate automated accounts, particularly content polluters [3]. We remove all messages that are retweets—either marked as such in the API's structured data, or any message containing the word *RT* (in either lowercase or uppercase). While retweeting could be a useful linguistic signal in its own right, we prefer to focus on original text. Finally, any message containing a URL is removed; this acts as a filter to remove automated and

marketing-oriented content, which is typically designed to draw the reader to a page elsewhere on the web. Of course, these filters also eliminate some legitimate messages, but since there is no shortage of data, we prefer to focus on a subsample that is more likely to contain original, non-automated content.

**Time** Each Twitter message includes a timestamp. We aggregate messages into seven-day intervals, which facilitates computation and removes any day-of-week effects. Each interval starts on Monday at UTC 0800, corresponding to 12am PST and 4am EDT.

# Words

To select the set of words to analyze, we begin with the 100,000 most frequent terms, excluding hashtags and usernames. We further require that each term must be used more than more than twenty times in ten different metropolitan areas. We compute the variance of the word's log probability over time ($\nu_{w,t}$ in Equation 2 of the main text, estimated in a standalone step, as described there), and require that the variance be greater than three. This cutoff was chosen so that roughly 5,000 words would be selected; we end up with 4,854 words. From this subset, we manually eliminate all named entities and non-English words. This determination is ambiguous because some strings can reference both names and words (e.g. *homer*, a dictionary word that often references the character *Homer Simpson*) or multiple languages (e.g. *y*, which can mean *and* in Spanish, and *why* in informal English). For each term, we randomly select twenty example messages and manually determine from context whether the usage is as an English word. We retain terms that are used as English non-name words in at least 80% of the examples.

The final word set contains 2,603 words. Our annotation decisions for all 4,854 words can be seen in our supplementary information file, *name_annotations.tsv*, and the selected words can be seen in *wordlist_table.pdf*. The usage examples we inspected are available in *word_examples_for_annotation_in_cluster_order.html*.

The overall results of our analysis are broadly similar when we do not perform manual word filtering, but this filtering enables us to focus on changes in (English) language rather than in the popularity of entities or in the overall multilingual composition of American Twitter users.

All text was tokenized using the *Twokenize.java* program, which can be downloaded at `http://www.ark.cs.cmu.edu/TweetNLP/`. Twokenize is designed to be robust to social media phenomena that confuse other tokenizers, such as emoticons [4, 5]. Repetition of the same character two or more times was normalized to just two (e.g. *sooooo* $\rightarrow$ *soo*). No other preprocessing (e.g., stemming) was performed.

# References

1. Office of Management and Budget (USA) (2010) 2010 standards for delineating metropolitan and micropolitan statistical areas. Federal Register 75.

2. Hecht B, Stephens M (2014) A tale of cities: Urban biases in volunteered geographic information. In: Proceedings of the International Workshop on Web and Social Media (ICWSM).

3. Lee K, Eoff BD, Caverlee J (2011) Seven months with the devils: A long-term study of content polluters on twitter. In: Proceedings of the International Workshop on Web and Social Media (ICWSM).

4. O'Connor B, Krieger M, Ahn D (2010) Tweetmotif: Exploratory search and topic summarization for twitter. In: Proceedings of the International Workshop on Web and Social Media (ICWSM).

5. Owoputi O, O'Connor B, Dyer C, Gimpel K, Schneider N, et al. (2013) Improved part-of-speech tagging for online conversational text with word clusters. In: Proceedings of the North American Association for Computational Linguistics (NAACL).
